# Deubiquitinases as novel therapeutic targets for diseases

**DOI:** 10.1002/mco2.70036

**Published:** 2024-12-13

**Authors:** Yali Xian, Jing Ye, Yu Tang, Nan Zhang, Cheng Peng, Wei Huang, Gu He

**Affiliations:** ^1^ Department of Dermatology & Venerology State Key Laboratory of Biotherapy West China Hospital Sichuan University Chengdu China; ^2^ State Key Laboratory of Southwestern Chinese Medicine Resources School of Pharmacy Chengdu University of Traditional Chinese Medicine Chengdu China

**Keywords:** deubiquitinating enzymes (dubs), deubiquitination, human diseases, signaling pathway regulation, targeted therapy

## Abstract

Deubiquitinating enzymes (DUBs) regulate substrate ubiquitination by removing ubiquitin or cleaving within ubiquitin chains, thereby maintaining cellular homeostasis. Approximately 100 DUBs in humans counteract E3 ubiquitin ligases, finely balancing ubiquitination and deubiquitination processes to maintain cellular proteostasis and respond to various stimuli and stresses. Given their role in modulating ubiquitination levels of various substrates, DUBs are increasingly linked to human health and disease. Here, we review the DUB family, highlighting their distinctive structural characteristics and chain‐type specificities. We show that DUB family members regulate key signaling pathways, such as NF‐κB, PI3K/Akt/mTOR, and MAPK, and play crucial roles in tumorigenesis and other diseases (neurodegenerative disorders, cardiovascular diseases, inflammatory disorders, and developmental diseases), making them promising therapeutic targets Our review also discusses the challenges in developing DUB inhibitors and underscores the critical role of the DUBs in cellular signaling and cancer. This comprehensive analysis enhances our understanding of the complex biological functions of the DUBs and underscores their therapeutic potential.

## Graphical abstract

Deubiquitinating enzymes (DUBs) are crucial for cellular homeostasis, modulating ubiquitination by removing ubiquitin or cleaving ubiquitin chains. With about 100 types in humans, DUBs balance the activity of E3 ubiquitin ligases, ensuring proteostasis and responses to stress. They regulate key pathways such as NF‐κB, PI3K/Akt/mTOR, and MAPK, influencing tumorigenesis and various diseases, including cancer, neurodegenerative diseases, cardiovascular diseases, inflammation, and developmental disorders. Highlighted for their structural and functional diversity, DUBs represent promising therapeutic targets. This overview addresses their roles in cellular signaling, cancer, and the challenges in developing effective DUB inhibitors.

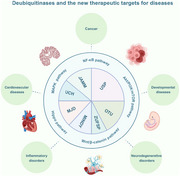

## 1 INTRODUCTION

Posttranslational modification (PTM) of proteins is a covalent alteration that occurs in the backbone or side chains of proteins following translation. This process can significantly influence the structure, function, and stability of proteins. Common PTMs include phosphorylation, acetylation, ubiquitination, SUMOylation, glycosylation, palmitoylation, and methylation. PTMs are integral to nearly all cellular life processes.^1^ Ubiquitination, a highly regulated PTM, involves the covalent attachment of ubiquitin—a small protein comprising 76 amino acid residues—to substrate proteins.^2^ This process is orchestrated by a cascade of enzymes: ubiquitin‐activating enzyme E1 first activates ubiquitin molecules; the activated ubiquitin is then transferred to ubiquitin‐conjugating enzyme E2; finally, ubiquitin ligase E3 facilitates the transfer of ubiquitin from E2 to the target protein, completing the ubiquitination process.^3^ Ubiquitination regulates protein–protein interactions, localization, and enzyme activity, and is involved in various biological processes such as DNA damage repair and cell cycle progression.^4^ The removal of ubiquitin from protein substrates, termed deubiquitination, is mediated by deubiquitinating enzymes (DUBs). DUBs reverse ubiquitination by recognizing and binding to substrates, cleaving the covalent bonds between substrates and ubiquitin, and releasing ubiquitin molecules, thereby protecting proteins from proteasome‐dependent degradation.^5^

**FIGURE 1:**
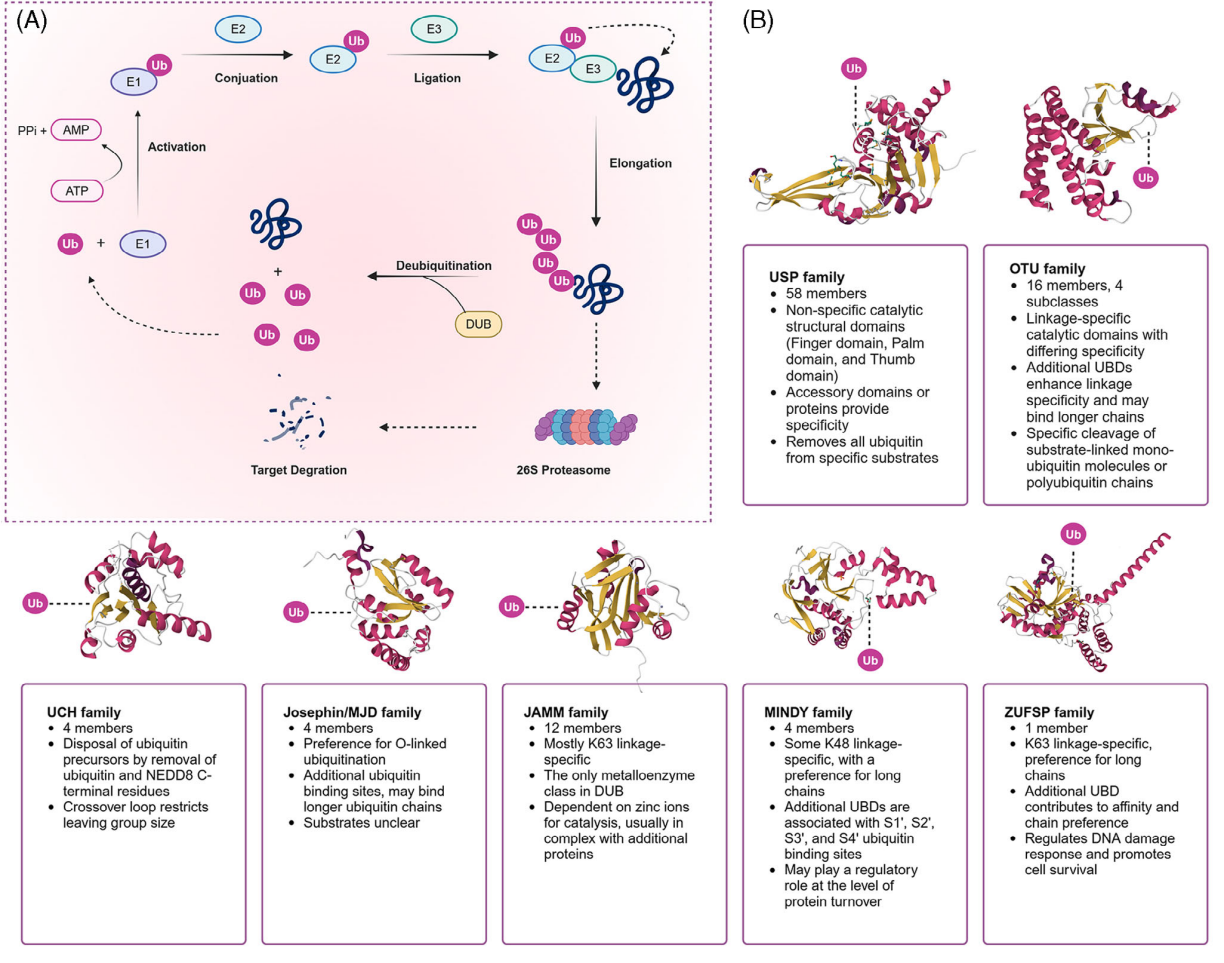
Ubiquitination and deubiquitination processes and the DUB family. (A) Schematic representation of the ubiquitination and deubiquitination processes. The ubiquitination process initiates with the activation of ubiquitin (Ub) by the E1 enzyme, which requires ATP and results in the formation of a ubiquitin‐AMP intermediate. Ubiquitin is subsequently transferred to the E2 enzyme, which then conjugates ubiquitin to the substrate protein via the E3 enzyme, forming a ubiquitin–protein conjugate. The ubiquitinated protein is targeted for degradation by the 26S proteasome. Deubiquitination, mediated by DUBs, involves the removal of ubiquitin from the substrate, thereby regulating protein stability and recycling ubiquitin. (B)The DUB family is divided into seven subfamilies, each possessing unique features and specific ubiquitin binding sites (Ub‐links). These subfamilies exhibit chain‐specific recognition and cleavage of various ubiquitin chains, with particular specificity for the Lys48 and Lys63 types of ubiquitin chains.

To date, approximately 100 DUBs encoded by the human genome have been identified, classified into seven subfamilies: ubiquitin‐specific proteases (USP), ubiquitin C‐terminal hydrolases (UCH), ovarian tumor proteases (OTU), Machado‐Josephin domain proteases (MJD), motif interacting with ubiquitin‐containing novel DUB family (MINDY), JAB1/MPN/Mov34 metalloenzymes (JAMM, the only metal‐dependent DUB family with metal ions as catalytic centers), and the recently discovered zinc finger‐containing ubiquitin peptidases^6^, ^7^, ^8^ (Figure 1A).

The USP family is one of the largest and most functionally diverse families of DUBs, comprising over 50 members in the human genome. These USP family members can recognize ubiquitin chains linked by K48, K63, and ME1, performing various critical functions within cells, ranging from protein degradation and signal transduction to cell cycle regulation and DNA repair, thereby modulating the stability and function of target proteins. As the second‐largest family of DUBs, OTUs possess specific OTU domains, and their members exhibit diversity in ubiquitin chain editing and substrate specificity. Unlike the USP family, which indiscriminately cleaves most types of ubiquitin chains, OTU family members may exhibit lower specificity for ubiquitinated proteins themselves but regulate the abundance of selected ubiquitin chain types under specific physiological conditions.^9^ Members of the UCH family are primarily responsible for removing the C‐terminal residues of ubiquitin and NEDD8, playing a crucial role in processing ubiquitin precursors and are essential in the intracellular ubiquitin cycle. The MJD family is known for its association with specific neurodegenerative diseases and its unique enzymatic characteristics, with these proteases tending to recognize O‐linked ubiquitination. The JAMM family is characterized by its metalloprotease activity, using a zinc‐dependent metalloprotease mechanism to recognize and cleave K63‐linked polyubiquitin chains. The MINDY family can interact with specific motifs within ubiquitin chains and tends to recognize and hydrolyze K48‐linked polyubiquitin from the distal end. ZUFSP is characterized as the prototype of a novel class of DUBs due to its unique protease fold and is the sole mammalian member, specifically binding and cleaving Lys63‐linked polyubiquitin (Figure 1B).

In recent years, DUB sub‐families have attracted significant research interest due to their unique deubiquitination preferences and crucial roles in various biological processes. These enzymes have been implicated in a wide array of human diseases, such as cancer, cardiovascular diseases, inflammatory disorders, and neurodegenerative conditions.^10^, ^11^, ^12^, ^13^, ^14^ The growing body of evidence linking DUBs to these diseases underscores their significance in human health and disease. As research has progressed, the critical role of the DUBs in oncology has also gained significant attention, leading to the gradual development of small molecule modulators targeting DUB proteins. Therefore, this review aims to offer a thorough examination of the fundamental aspects of the DUB family and to summarize recent significant research findings. We focus on the structural determinants that govern substrate and chain‐type specificity among DUB family members. And we explore the roles of DUB proteins in regulating key signaling cascades, such as the NF‐κB and MAPK pathways. Finally, we discuss their dual roles as tumor suppressors and oncogenes in various cancer types, and their regulatory functions in other diseases, including neurodegenerative disorders, cardiovascular diseases, inflammatory disorders, and developmental diseases. This comprehensive review aims to provide valuable insights into the development of selective DUB inhibitors and their potential clinical applications in targeted therapy and immunomodulation, thereby contributing to future research directions in this field.

## 2 STRUCTURE AND FUNCTION OF DUBs

### 2.1 Structure of DUBs

**FIGURE 2:**
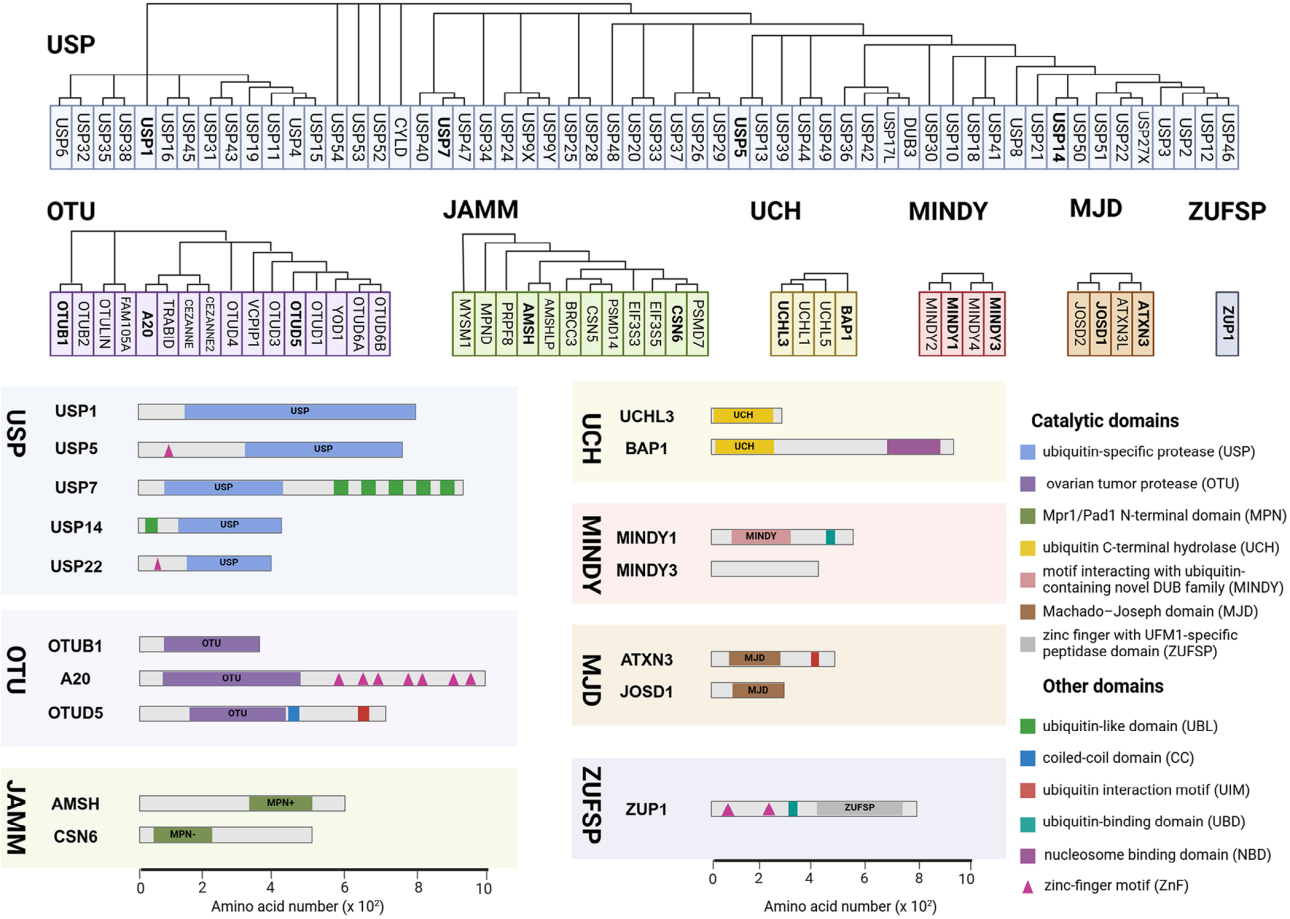
Phylogenetic tree of each DUB subfamily and schematic diagrams of various representative protein domains. The phylogenetic tree of each DUB subfamily is drawn based on the protein sequence information of its members. And schematic diagrams of the structures of representative members in each subfamily are shown.

DUBs generally consist of one or more binding domains for substrate recognition, such as zinc‐finger (ZnF) motifs, UBD, ubiquitin‐binding domains (UBD), ubiquitin‐like domains (UBL), coiled‐coil domains, and ubiquitin interaction motifs (UIM), which work in conjunction with indispensable catalytic domains to precisely control hydrolysis. The catalytic domains contain the classical catalytic triad necessary for the hydrolysis of ubiquitin chains, including cysteine (Cys), histidine (His), and aspartic acid (Asp) or glutamic acid residues.^15^ Other domains work in concert to catalyze and precisely regulate hydrolysis while influencing cellular localization and functional diversity.^16^, ^17^, ^18^ Most identified DUB families belong to Cys proteases, except for the JAMM family, which is classified as zinc metalloproteases. DUBs with Cys protease activity typically exhibit a catalytic pocket composed of Cys, His, and acidic residues, while JAMM family DUBs are characterized by their zinc‐dependent metalloprotease activity, coordinating zinc ions with aspartate, His, and serine residues to facilitate water molecule recruitment and initiate isopeptide bond hydrolysis (Figure 2).

Due to their conserved structure and complex functional mechanisms, USP family members are widely present in eukaryotes, exhibiting a high degree of evolutionary conservation. The core of USP family members is the USP domain, characterized by a highly conserved catalytic core, which typically consists of nine tandem ubiquitin‐like folds (Ubs) and two large insertion sequences. The three‐dimensional structure of the USP domain exhibits a unique α/β fold, which includes multiple β sheets and α helices, providing a stable framework that allows USP family members to efficiently recognize and hydrolyze ubiquitin or ubiquitin chains. USP1 consists of 785 amino acids and features a conserved USP domain typical of the DUB family, which includes the catalytic residues (Cys90, His593, and Asp751).^19^ USP5 consists of a ZnF domain and a USP domain with a catalytic core composed of a Cys box and a His box and two ubiquitin‐associated motifs.^19^ USP7 is a well‐studied USP protein among nearly 100 DUBs that is characterized by a structure consisting of 1102 amino acids, including a catalytic core domain flanked by an N‐terminal TRAF‐like domain and five C‐terminal UBL domains.^20^ The catalytic core domain, formed by residues Cys223, His464, and Asp481, is primarily responsible for cleaving the isopeptide bond between ubiquitin and substrate proteins.^21^ The UBL domains of USP7 contribute to interactions with various proteins, particularly in substrate recognition and specificity, while the unique TRAF‐like domain aids in substrate binding and initiating conformational changes necessary for catalytic activity.^22^ USP14 is composed of 494 amino acids and features two distinct domains: an N‐terminal UBL domain that regulates proteasome activity and a C‐terminal USP domain responsible for its DUB activity.^23^ USP22 encodes a peptide with 525 amino acids and has a molecular weight of approximately 60 kDa. Its structure comprises an N‐terminal ZnF domain and a C‐terminal catalytic domain. Unlike other DUBs, the ZnF domain in USP22 does not directly bind to ubiquitin but instead interacts with other proteins to form a tightly locked deubiquitinase module, which deubiquitinates target proteins to alter their expression.^24^

The OTU domain is the core of OTU family members, characterized by a conserved protein structure consisting of five beta strands flanked by alpha helical domains of varying sizes. This structural arrangement provides a stable framework for OTU family members to efficiently recognize and cleave ubiquitin or ubiquitin chains.^25^, ^26^ The catalytic mechanism of OTUs involves the initial recognition and binding to ubiquitin substrates or ubiquitin chains. The sulfur atom of the Cys residue at the catalytic center attacks the carbonyl carbon atom of the isopeptide bond between ubiquitin and substrate proteins or within the ubiquitin chain. The adjacent His residue regulates the p*K*_a_ of the catalytic Cys, while Asp or asparagine stabilizes and polarizes the catalytic histidine, forming a catalytic intermediate. This intermediate is subsequently dissociated through the participation of water molecules, leading to the hydrolysis of bonds between ubiquitin and substrate protein or within the ubiquitin chain. The cleaved ubiquitin and substrate protein (or the remaining part of the ubiquitin chain) are then dissociated from the enzyme, completing the deubiquitination process.^9^, ^27^, ^28^ The unique crystal structure of OTUB1 provides it with two distinct ubiquitin‐binding sites—a distal site and a proximal site containing approximately 45 N‐terminal residues of OTUB1.^29^ Studies have shown that the activity of the OTUB1 enzyme is closely related to the regulation of E2 enzymes. OTUB1 not only exhibits classical DUB activity by directly removing ubiquitin molecules attached to substrates, but also possesses nonclassical DUB activity by binding to E2 enzymes to inhibit the ubiquitination process.^30^, ^31^ Unlike other DUBs, A20 exhibits both DUB activity and E3 ubiquitin ligase activity. The N‐terminal OTU domain endows A20 with DUB activity, while the C‐terminal ZnF domain confers E3 ligase activity.^32^ OTUD5 consists of 571 amino acids and contains two conserved structural domains: the OTU domain and the UIM domain.^24^

The MPN (Mpr1/Pad1 N‐terminal) domain is a notable feature of the JAMM family, characterized by a crystal structure that includes an eight‐stranded beta sheet and two alpha helices. MPN domain proteins can be further classified into two subfamilies: the MPN+ family, which exhibits heteropeptidase activity, and the MPN– family, which lacks catalytic activity. The MPN+ family contains a zinc‐coordinated JAMM motif, while the MPN– family often functions as a scaffold in various JAMM multisubunit complexes.^33^ All MJD family members share a common Josephin domain that consists of approximately 180 amino acids. Ataxin‐3 (ATXN3) and ATXN3‐like protein (ATXN3L) features an N‐terminal Josephin domain and a flexible C‐terminal domain with UIMs. Although JOSD1 and JOSD2 contain only a single Josephin domain, this domain is highly conserved across all eukaryotes, indicating the essential role of MJD family members despite their largely unexplored biological functions.^34^ The conserved UCH catalytic domain with classical α‐β‐α folding is a characteristic shared by all UCH family members. UCHL1 is composed of 223 amino acids and encompasses a UCH catalytic domain with short N‐terminal and C‐terminal extensions.^35^ BAP1, consisting of 729 amino acids, includes the UCH catalytic domain and the C‐terminal nucleosome binding domain.^35^, ^36^

In summary, the conserved catalytic triad and additional functional domains endow DUB family members with precise substrate specificity and regulatory capabilities, underscoring their multifaceted roles in cellular processes. This foundational understanding of DUB domains sets the stage for exploring their broader biological implications and therapeutic potential.

### 2.2 Catalytic mechanisms of DUBs in recognition and cleavage

The catalytic domains of DUBs recognize and bind modifications through at least one ubiquitin‐binding site, namely, the S1 site.^37^ DUBs can either directly bind the substrate to remove the ubiquitin attached to it, or recognize and cleave the ubiquitin chain itself. Cys protease DUBs typically contain a catalytic triad composed of Cys, His, and an acidic residue. The catalytic process involves the deprotonated Cys residue attacking the isopeptide bond to form a tetrahedral intermediate, releasing the proximal ubiquitin, followed by hydrolysis to form a second tetrahedral intermediate, ultimately releasing the distal ubiquitin and regenerating the apo‐enzyme. Zinc‐dependent metalloprotease DUBs (JAMM family) have an active site comprising a zinc atom coordinated by conserved catalytic residues and a water molecule. The catalytic process involves a nucleophilic attack by the activated water molecule, forming a tetrahedral intermediate, cleaving the isopeptide bond, and simultaneously releasing the two ubiquitin parts. The arrangement and nature of ubiquitin binding sites in DUBs determine their ability to cleave polyubiquitin chains either exo‐ (distal or proximal) or endo‐cleavage.

Most USP and UCH subfamily members exhibit nonspecific interactions with substrates, while other subfamilies such as OTU, JAMM, Josephine, and MINDY demonstrate chain specificity, particularly the OTU family.^38^ For instance, OTUB1 demonstrates a preference for Lys48‐linked chains, which typically tag proteins for degradation via the 26S proteasome. In contrast, OTUD1 preferentially cleaves Lys63‐linked chains, which are associated with nondegradative functions such as DNA repair, signal transduction, and endocytosis.^17^ Other OTU family members, such as OTUD7B, show specificity for Lys11‐linked chains.^39^, ^40^ OTULIN, on the other hand, specifically targets Met1‐linked linear chains, which play a critical role in the activation of the NF‐κB signaling pathway.^41^ TRABID exhibits specificity for Lys29‐ and Lys33‐linked chains, which may have distinct functions in regulating specific cellular signaling processes.^42^The OTU family exhibits linkage specificity or debranching characteristics by binding ubiquitin chains at specific S1′ and S2′ sites. The study of DUB cleavage mechanisms indicates that OTUs cleave ubiquitin chains by interacting with the distal and proximal parts of the ubiquitin chain at the S1 and S1′ sites, respectively. The S1′ site of OTUs binds to the proximal ubiquitin, allowing only one connection point of the proximal ubiquitin to enter the catalytic center. Therefore, the S1′ site is the determining factor for chain‐specific cleavage. Many S1′ sites are located on the catalytic domain of OTUs. Additional Ub binding sites such as S2, S2′, or auxiliary UBDs enhance the functional capacity of DUBs.^9^ For example, OTUD2 can specifically recognize and cleave longer Lys11‐linked chains, a specificity enhanced by the S2 site within the OTU domain.^9^

In summary, the arrangement of ubiquitin binding sites (S1, S1′, S2, S2′) determines their ability to perform either exo‐ or endo‐cleavage, contributing to their substrate specificity and catalytic efficiency. Further research on catalytic mechanisms of DUBs is needed to understand their intracellular functions and may provide new targets for drug development, with significant scientific and clinical implications.

### 2.3 Regulation of DUB enzymatic activity

The activity of DUBs influenced not only by their inherent structural features but also by various intracellular signaling and regulatory mechanisms. These include PTMs, protein–protein interactions, conformational changes, and cellular environmental factors. Such regulatory mechanisms ensure that DUB function at the appropriate time and location, thereby preventing potential cellular damage due to uncontrolled activity. Dysregulation of DUB activity is closely associated with the pathogenesis of various diseases, particularly cancer, neurodegenerative diseases, and inflammatory conditions.

#### 2.3.1 Posttranslational modifications

PTMs are among the primary mechanisms regulating the activity of DUBs. These modifications can profoundly impact the function, stability, substrate affinity, localization, and interaction capabilities of proteins. DUBs may undergo various types of PTMs, including but not limited to phosphorylation, ubiquitination, and acetylation.

Phosphorylation is a common PTM that regulates the activity and substrate specificity of DUBs. CYLD's activity is regulated by a phosphorylation switch outside its catalytic USP domain, specifically at Ser568 and Ser418, enhancing its deubiquitinating function toward Lys63‐linked polyubiquitin.^43^ CDK1 phosphorylates and activates USP29, enhancing its deubiquitinase activity toward TWIST1.^44^ Phosphorylation of OTUD5 at the Ser177 site activates its deubiquitination activity and confers specificity for particular substrates, which is crucial for the cellular response to DNA damage.^45^ Similarly, phosphorylation of OTUD3 enhances its binding affinity to the transcription factor YY1, thereby stabilizing YY1 and promoting colorectal cancer (CRC) progression.^46^ Notably, phosphorylation can also alter the specificity of DUBs for ubiquitin chain hydrolysis. OTUD4, typically a Lys48 chain‐specific DUB, exhibits selectivity toward the Lys63 bond in cell extracts, a specificity conferred by OTUD4 phosphorylation.^47^

Members of the OTU family can also serve as substrates for ubiquitination, which can regulate their stability, affinity, or interactions with other proteins, thereby affecting their activity. For example, USP30 is ubiquitinated by Parkin that promotes the its degradation through the proteasome pathway, thereby enhancing mitophagy by removing the inhibitory effect of USP30 on this process.^48^ USP35 can be ubiquitinated by CHIP/HSP90, but its unique homodimer structure is essential for auto‐deubiquitination to counteract this process.^49^ The ubiquitinase TRIM54 can bind and ubiquitinate YOD1, regulating inflammatory responses in muscle stem cells.^50^

Acetylation is another key PTM that affects the substrate recognition and catalytic activity of DUB enzymes by adding acetyl groups. Under normal conditions, the acetylation state of OTUD3 enables it to effectively cleave Lys63‐linked ubiquitin chains on MAVS, preventing overactivation of innate immune responses and providing antiviral defense. However, during viral infection, OTUD3 may be deacetylated and inactivated by SIRT1, leading to MAVS aggregation and an enhanced immune response.^51^

In addition to PTMs, changes in the redox state are also crucial for regulating the activity of DUBs As Cys proteases, OTU enzymes contain key active Cys residues that can be oxidized by reactive oxygen species (ROS), thereby affecting enzyme activity. This oxidative process is usually reversible, but under oxidative stress conditions, the activity of OTU enzymes may be significantly inhibited.^52^

#### 2.3.2 Protein interactions

Protein–protein interactions are a crucial mechanism for regulating the activity of DUBs. DUBs can form complexes with other proteins, thereby altering their substrate specificity, catalytic efficiency, cellular localization, and response to cellular signals. Certain proteins can bind to OTU enzymes, enhancing their activity. For example, USP22 associates with Ataxin‐7 (ATXN7/Sgf73), Ataxin‐7 like protein 3 (ATXN7L3/Sgf11), and ENY2 (ENY2/Sus1) to create a tightly locked tetrameric deubiquitinase module (DUBm) within the SAGA complex, performing deubiquitination on target proteins.^53^ WAC can directly bind with VCPIP1 to activate its deubiquitination activity and form complexes with VCPIP1 and p97, participating in Golgi membrane fusion.^54^

In addition to directly recognizing ubiquitin chains, some DUBs can regulate enzyme specificity by interacting with other proteins through specific scaffolds. For instance, OTUB1 can bind to FOXM1 via the protein scaffold LINC00857, forming a stable interaction complex that enhances its specificity and inhibits the degradation of FOXM.^55^ OTULIN is the only deubiquitinase in the OTU family specific for linearly linked ubiquitin chains. OTULIN interacts with the linear ubiquitin chain assembly complex (LUBAC) through a conserved PUB interaction motif, thereby regulating NF‐κB signaling, which is crucial for controlling inflammatory responses and immune regulation.^56^

Conversely, protein interactions can also inhibit enzyme activity. Early studies have shown that some DUBs form inhibitory complexes with other proteins, thereby reducing their activity. For example, the inhibitory complex formed by OTUB1 with charged E2∼Ub thioesters and free ubiquitin can inhibit OTUB1's DUB activity.^57^ However, there are currently few reports on how other proteins interact with OTU enzymes to inhibit their activity. The inhibitory effect of these interactions provides a potential pathway for developing new DUB inhibitors. These protein interactions not only reveal the complexity of DUBs in ubiquitin signaling regulation but also emphasize their diversity and importance in cellular physiology.

#### 2.3.3 Allosteric regulation

Allosteric regulation is another mechanism that modulates the activity of the DUB, involving changes in the three‐dimensional structure of proteins induced by molecular chaperones, intracellular small molecules, or other proteins. Activation of USP members mediated by WD40 repeat‐containing proteins provides one of the most notable examples of DUB allosteric regulation, where activate several USPs. USP1, USP12, and USP46 are allosterically activated by USP1 associated factor 1 (UAF1, also known as WDR48), while USP12 and USP46 are further costimulated by another WD40 repeat protein, WDR20^58^. Complex crystal structures of USP46 and USP12 reveal how UAF1 interacts with the USP finger subdomain and how WDR20 binds beneath the palm subdomain.^59^ The binding of these regulators occurs far from the catalytic center, with allosteric activation resulting from the stabilization of flexible elements and multiple subtle structural changes that translate to the active site.^60^ USP1 forms similar interactions with UAF1 and is likely regulated by similar mechanisms, albeit with additional regulatory mechanisms due to its larger insertions in the USP domain.^61^ The binding of free ubiquitin to OTUB1 triggers conformational changes in the OTU domain and forms a ubiquitin‐binding helix at the N‐terminus, thereby promoting an increase in the affinity of OTUB1 for UBC13∼Ub, which is crucial for OTUB1 to inhibit UBC13 and other E2 enzymes.^29^ OTUD7B is the only DUB known to have specificity for Lys11‐linked polyubiquitin. OTUD7B undergoes conformational rearrangement when interacting with Lys11‐linked ubiquitin substrates, which involves the instantaneous formation and disappearance of the S1′ site, helping it correctly recognize ubiquitin sites and endow them with locking specificity.^40^

In summary, the regulation of DUB deubiquitinase activity is multifaceted, involving PTMs, protein–protein interactions, and allosteric mechanisms. These regulatory pathways ensure precise temporal and spatial control of DUB activity, which is essential for maintaining cellular homeostasis and preventing pathogenesis. Disruptions in these regulatory mechanisms are closely associated with a variety of diseases, highlighting their importance in cellular physiology. Understanding these processes provides crucial insights into ubiquitin signaling and offers potential avenues for therapeutic intervention.

## 3 SIGNALING PATHWAYS REGULATED BY DUBs IN CANCER

DUBs regulate protein stability and activity while significantly influencing cell signaling processes, including cell growth, differentiation, apoptosis, and stress responses. In cancer, DUB family members affect multiple tumor‐related signaling pathways, such as NF‐κB, PI3K/Akt/mTOR, Wnt/β‐catenin, Hippo, and TGF‐β, by precisely regulating key molecules like transcription factors and signaling proteins. These actions are closely linked to tumor initiation and progression, highlighting the pivotal role of DUBs in tumorigenesis and tumor progression. This section examines the role of DUBs in regulating key cellular signaling cascades, aiming to enhance our understanding of tumor biology and explore their potential as drug targets.

### 3.1 NF‐κB signaling pathway

**FIGURE 3:**
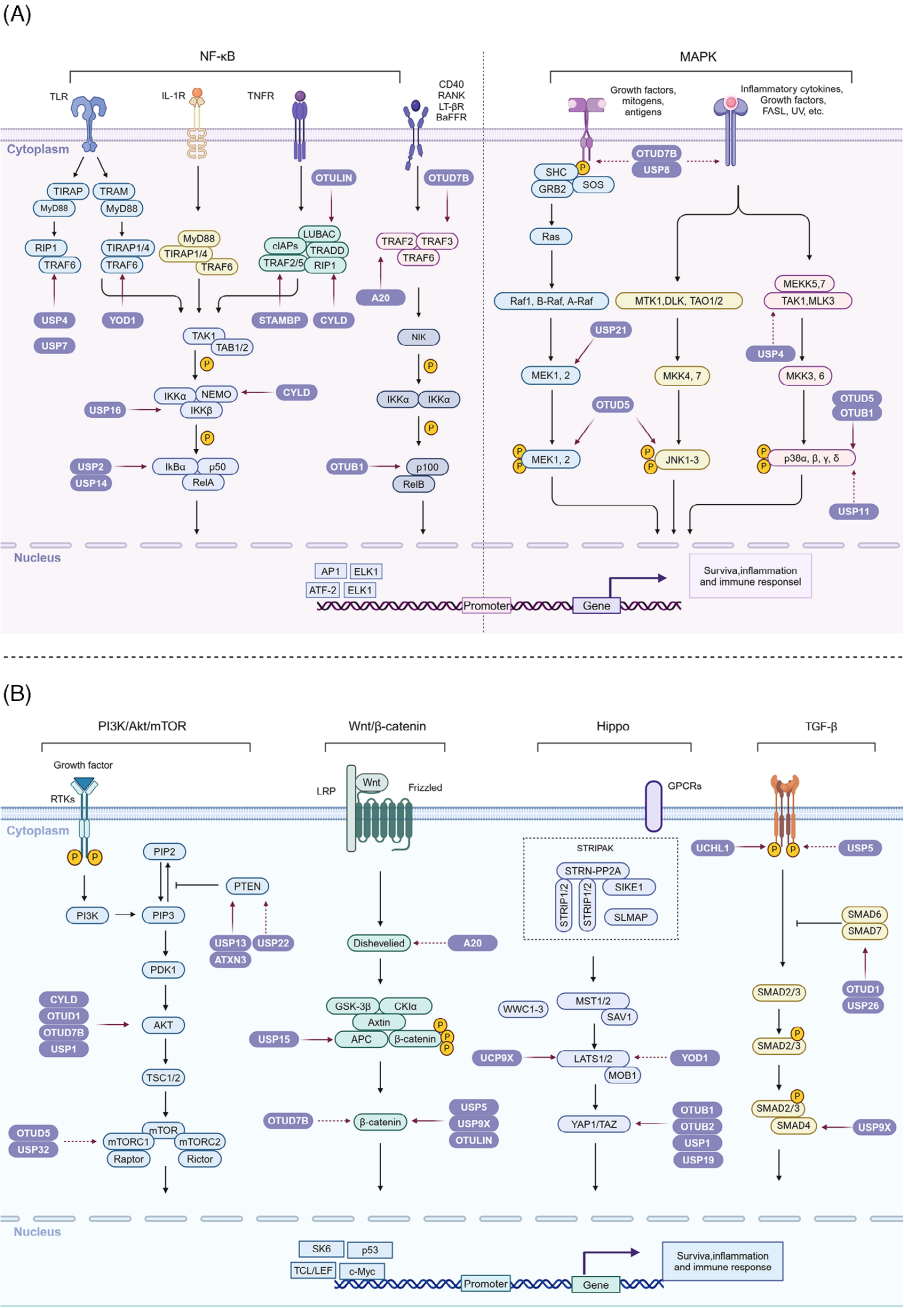
Regulatory roles of the DUB family in signaling pathways. (A) The left panel illustrates the NF‐κB signaling pathway, while the right panel depicts the MAPK signaling pathway. In the NF‐κB pathway, DUB family members regulate key components such as TRAF6, IKK, and NF‐κB subunits (p50, RelA) either directly or indirectly. In the MAPK pathway, DUBs modulate components like MEK1/2, ERK1/2, and p38 MAPK, thereby influencing downstream gene expression and cellular responses. (B) The panels from left to right demonstrate the involvement of the DUB family in regulating the PI3K/Akt/mTOR, Wnt/β‐catenin, Hippo, and TGF‐β signaling pathways. Solid lines represent the direct regulation of key components within these pathways by DUB enzymes, whereas dashed lines indicate the indirect regulation through modulation of other substrates, which subsequently affect the key components and signal transduction within these pathways.

The NF‐κB signaling pathway is fundamental for regulating immune responses and inflammation. Maintaining its precise control is crucial to ensure appropriate cellular responses and to prevent diseases such as chronic inflammation and cancer.^62^ DUBs are critical modulators within this pathway, targeting various key proteins to fine‐tune the signaling processes. Here, we will delve into the comprehensive and intricate regulation of the NF‐κB pathway by different DUBs, focusing on their specific roles and mechanisms of action (Figure 3A).

The NF‐κB family includes transcription factors such as p105/p50, p100/p52, p65 (RelA), RelB, and c‐Rel, activated primarily through the canonical and noncanonical pathways. The classical pathway responds to proinflammatory signals like TNF‐α and LPS, activating the IκB kinase (IKK) complex, composed of IKKα, IKKβ, and NEMO. This activation leads to phosphorylation, ubiquitination, and proteasomal degradation of IκB proteins, releasing NF‐κB dimers to translocate to the nucleus and initiate transcription of target genes involved in inflammation and immune response.^63^, ^64^

#### 3.1.1 IκBα and IKK complex regulation

DUBs play pivotal roles in regulating the NF‐κB pathway by modulating the activity and stability of IκBα and the IKK complex. USP2, for instance, is critical in the TNF‐α‐induced NF‐κB signaling cascade. It facilitates the phosphorylation of IκBα, leading to its ubiquitination and degradation, which in turn promotes the nuclear translocation of NF‐κB dimers. This process is essential for the transcription of NF‐κB‐dependent genes, such as IL‐8, showcasing USP2's integral role in inflammatory responses.^65^

USP14 significantly influences IκBα dynamics. Overexpression of USP14 enhances the phosphorylation and ubiquitination of IκBα, followed by its proteasomal degradation. This promotes the release and activation of NF‐κB, subsequently leading to the production of proinflammatory cytokines like TNF‐α and IL‐8.^66^ USP14's function is modulated further by serine phosphorylation in response to LPS, illustrating a complex regulatory mechanism that adjusts the NF‐κB pathway intensity and duration in response to external stimuli.^67^

USP16 has been found to interact with both IKKα and IKKβ, specifically deubiquitinating them at Lys33‐linked chains. This deubiquitination process facilitates IKKβ‐mediated phosphorylation of p105, thereby promoting NF‐κB activation independent of direct IκBα or p65 phosphorylation.^68^ USP16's regulation is particularly crucial in autoimmune responses and inflammatory diseases, such as inflammatory bowel disease (IBD) and colon cancer, underscoring the diverse mechanistic roles of DUBs in NF‐κB signaling control.

The LUBAC adds Met1‐linked linear polyubiquitin chains to specific target proteins, playing a critical role in NF‐κB activation. OTULIN specifically targets these linear chains, efficiently removing them and thus downregulating NF‐κB signaling.^69^ This action is essential for preventing excessive and potentially pathological NF‐κB activation, showcasing OTULIN's role in maintaining immune homeostasis and inhibiting inappropriate inflammatory responses.

#### 3.1.2 TRAF family proteins

TRAF proteins, including TRAF2, TRAF3, and TRAF6, are central hubs in NF‐κB signaling, receiving regulation by various DUBs. USP4 regulates NF‐κB signaling negatively by targeting TRAF6, removing Lys63‐linked polyubiquitin chains and inhibiting its function.^70^ This removal prevents inappropriate NF‐κB activation, maintaining a balance in inflammatory responses. YOD1 competes with p62 to bind TRAF6 in unstimulated cells, preventing TRAF6's premature recruitment to signaling complexes. Upon IL‐1β stimulation, YOD1 is released, facilitating TRAF6 autoubiquitination and subsequent NF‐κB component recruitment, ensuring timely and controlled NF‐κB pathway activation.^71^

USP7, also known as HAUSP, is another DUB that associates with TRAF6, inhibiting its polyubiquitination and subsequent signaling capabilities.^72^ This interaction highlights USP7's role in immune regulation and its impact on bone remodeling through the inhibition of osteoclast formation, driven by NF‐κB signaling modulation.

CYLD is extensively characterized for its role as a negative regulator in the NF‐κB pathway. It deubiquitinates multiple key signaling molecules, such as NEMO, RIP1, TRAF2, TRAF6, TRAF7, TRADD, and RIPK1, by removing polyubiquitin chains. These actions prevent their degradation or inappropriate activation, acting as a critical checkpoint in NF‐κB signaling and ensuring cellular homeostasis.^73^, ^74^, ^75^, ^76^

STAMBPL1, by stabilizing TRAF2 through deubiquitination at K63, promotes NF‐κB signaling by enhancing the nuclear translocation of p65. This stabilization is critical for maintaining the integrity of downstream signaling cascades associated with inflammatory and immune responses.^77^

#### 3.1.3 NIK and noncanonical pathway regulation

In the noncanonical pathway, DUBs like OTUD7B are crucial for regulating NIK stability. OTUD7B specifically deubiquitinates TRAF3, stabilizing it and ensuring the continuous degradation of NIK. This regulation prevents aberrant p100 processing into p52, thereby tightly controlling noncanonical NF‐κB pathway activation and ensuring appropriate immune responses.^78^, ^79^

A20 (TNFAIP3) emerges as a pivotal regulator across both NF‐κB pathways. It employs a complex catalytic mechanism to disassemble polyubiquitin chains, first removing Lys63‐linked chains and then tagging proteins with Lys48‐linked chains for degradation. In the NF‐κB pathway, A20 deubiquitinates key proteins, including TRAF6, NEMO, and RIP1, thereby impeding their activation.^80^ A20's action extends to TRAF2, disrupting its signal transducing functions and providing a broad‐spectrum regulation of NF‐κB signaling, essential for resolving inflammation and immune responses.^81^

The regulation of the NF‐κB signaling pathway by DUBs involves a sophisticated network of ubiquitination and deubiquitination processes essential for maintaining cellular and physiological homeostasis. By fine‐tuning the ubiquitination status of key signaling molecules such as IκBα, IKK complexes, TRAF proteins, NIK, and components of the LUBAC complex, DUBs orchestrate precise control over NF‐κB activity. This regulation is vital for proper immune responses, preventing inappropriate inflammation, and providing therapeutic targets for diseases characterized by chronic inflammation and deregulated NF‐κB signaling. The detailed mechanistic understanding of DUB‐mediated regulation offers valuable insights into potential interventions for modulating NF‐κB pathways in various disease contexts, highlighting the therapeutic potential of targeting specific DUBs to achieve desired outcomes in immune and inflammatory conditions.

### 3.2 MAPK signaling pathway

The MAPK signaling pathway is essential for transmitting signals from extracellular stimuli, impacting cancer cell behavior, including proliferation, survival, and drug resistance. This pathway involves a cascade of phosphorylation events triggered by membrane receptor engagement, leading to MAPKKK and MAPKK activation, and culminating in MAPK regulation of gene expression. This complex signaling network is modulated by DUBs, which influence various MAPK pathway branches, including ERK, JNK, and p38 MAPK^82^ (Figure 3A).

The ERK pathway is central to promoting cell proliferation and survival in response to growth factors. This pathway is tightly regulated by various DUBs to ensure proper signal transduction. For instance, USP21 is crucial for maintaining the stability of MAPK/ERK kinase (MEK) 2, a core component of the ERK pathway. By deubiquitinating MEK2, USP21 ensures sustained activation of ERK, which is vital for the progression of hepatocellular carcinoma (HCC).^83^ This stabilization mechanism underscores the role of DUBs in prolonging the active state of pivotal signaling proteins.

Another key regulator is USP4, which stabilizes transforming growth factor‐beta (TGF‐β)‐activated kinase 1 (TAK1), thus influencing the ERK pathway indirectly. TAK1 activation leads to downstream signaling cascades including the ERK pathway, highlighting the interconnectedness of MAPK pathways and the broad regulatory impact of DUBs like USP4.^84^

Moreover, USP8 and OTUD7B (Cezanne‐1) modulate the EGFR signaling axis, a significant upstream activator of the ERK pathway.^85^, ^86^ By deubiquitinating and stabilizing EGFR, these DUBs amplify the transmission of proliferative signals through the ERK cascade, showcasing their role in enhancing growth factor‐mediated signaling.

The JNK pathway responds primarily to stress stimuli and is pivotal in controlling apoptosis and cellular differentiation. OTUD1 emerges as a critical player in this pathway by stabilizing apoptosis signal‐regulating kinase 1 (ASK1), an upstream MAPK kinase kinase.^87^ OTUD1's action ensures ASK1's activity, thereby promoting the activation of JNK signaling in response to stress signals. Interestingly, OTUD1 achieves this stabilization independently of its deubiquitinase function, suggesting a unique regulatory mechanism that extends beyond conventional deubiquitination.

Furthermore, OTUD5 exhibits a broad influence by inhibiting the phosphorylation of ERK1/2, p38, and JNK, thereby providing a counterbalance within the MAPK signaling network.^88^ This inhibition is crucial for finely tuning cellular responses to avoid excessive apoptosis or uncontrolled cell proliferation that could result from aberrant JNK activation.

The p38 MAPK pathway is activated in response to stress and inflammatory cytokines, playing a critical role in apoptosis, cell differentiation, and immune responses. OTUB1 acts as a key modulator in this pathway by inhibiting the sustained activation of LPS‐induced p38 MAPK signaling.^89^ This action serves as a preventive measure against chronic inflammation and associated pathologies, highlighting OTUB1's role in maintaining cellular homeostasis under stress conditions.

USP11 contributes to the regulation of the p38 pathway in a PPP1CA‐dependent manner. PPP1CA, one of the catalytic subunits of protein phosphatase 1, influences MAPK signaling and is protected from proteasome‐mediated degradation by USP11. This protection allows for enhanced activity of the p38 pathway, particularly in tumorigenesis processes seen in CRC cells. By controlling PPP1CA levels, USP11 indirectly modulates p38 MAPK signaling, underscoring the complex interplay between DUBs and MAPK pathways.^90^

The regulation of MAPK pathways by DUBs is crucial for normal cellular function. Dysregulation can lead to conditions such as cancer, neurodegenerative diseases, and chronic inflammation. The complexity of DUB regulation is highlighted by their overlapping roles and interactions within MAPK signaling, converging on key molecules and pathways. This intricate network ensures properly scaled and timed cellular responses, preventing aberrant signaling. Understanding the specific roles of DUBs in MAPK pathways could reveal new therapeutic strategies for treating diseases linked to dysregulated MAPK signaling.

### 3.3 PI3K/Akt/mTOR signaling pathway

The PI3K/Akt/mTOR signaling pathway is crucial for regulating cell growth, proliferation, and survival. Dysregulation of this pathway is often implicated in various cancers and other diseases.^91^ DUBs play significant roles in modulating this pathway by influencing the stability and activity of key signaling proteins. This section explores the complex regulation of the PI3K/Akt/mTOR pathway by DUBs, focusing on their mechanisms and impact on critical components (Figure 3B).

The PI3K pathway is activated when extracellular growth factors bind to receptor tyrosine kinases (RTKs) on cell surfaces. This binding activates PI3K, which converts PIP2 into PIP3. PIP3 recruits Akt (protein kinase B) to the cell membrane, where Akt is activated by phosphoinositide‐dependent kinase‐1 (PDK1). Activated Akt phosphorylates various downstream targets, including the mechanistic target of rapamycin (mTOR), a central regulator of cell growth and metabolism. mTOR functions in two distinct complexes, mTORC1 and mTORC2, each playing unique roles in cellular metabolism and growth.^92^

Central to the regulation of the PI3K/Akt pathway is PTEN, which dephosphorylates PIP3 back to PIP2, antagonizing PI3K signaling. PTEN's tumor suppressor function is tightly regulated by ubiquitination, with several DUBs influencing its stability and activity. USP22‐mediated upregulation of SIRT1 leads to PTEN deacetylation, thus activating the PI3K/Akt/mTOR pathway, an effect that can be reversed by SIRT1 knockdown.^93^

Additional interactions include USP13, which directly binds and deubiquitinates PTEN, stabilizing it and suppressing tumorigenesis in PTEN‐positive cancer cells. The loss of USP13 results in enhanced Akt phosphorylation and tumor growth.^94^ Similarly, members of the Josephin family, including ATXN3, ATXN3L, and JOSD1, increase the expression of PTEN and its competing endogenous RNA PTENP1, indicating a transcriptional regulatory mechanism.^95^

Akt is a central node in the PI3K/Akt/mTOR pathway, and its activity is tightly controlled by phosphorylation and ubiquitination status. CYLD, a well‐characterized DUB, removes ubiquitin moieties from Akt, thereby preventing its activation in the absence of growth factors. Upon stimulation, CYLD dissociates from Akt, allowing E3 ligases to ubiquitinate and activate Akt.^96^

Similarly, OTUD1 and OTUD7B remove K63‐linked ubiquitination from Akt under different conditions, promoting its membrane recruitment and phosphorylation, thereby activating the pathway.^97^, ^98^ Interestingly, USP1 also interacts with Akt but primarily inhibits its activity by suppressing phosphorylation at T308, while complete Akt inhibition involves the complex formation with PHLPP1, which dephosphorylates S473.^99^ This coordination ensures precise control over Akt signaling during nutrient deprivation.

mTOR functions in two distinct complexes, mTORC1 and mTORC2, each regulating different aspects of cell physiology. A critical interaction involving OTUB1 shows that it stabilizes RACK1, thus preventing abnormal activation of the PI3K/Akt pathway. OTUD5 stabilizes RNF186, which promotes the degradation of the mTORC1 feedback inhibitor sestrin2, thereby activating mTOR signaling. Additionally, OTUD5 modulates the protein abundance of DEPTOR, an inhibitory subunit of both mTORC1 and mTORC2.^100^

Another intriguing regulator is USP32, whose knockout results in hyperubiquitination of the Ragulator complex subunit LAMTOR1. This modification impairs its interaction with vacuolar H‐ATPase, reducing Ragulator function and ultimately limiting mTORC1 recruitment. Consequently, mTOR kinase localization to lysosomes diminishes, leading to decreased mTORC1 activity and increased autophagy.^101^

The complexity of DUBs in regulating the PI3K/Akt/mTOR pathway highlights their potential as therapeutic targets. In summary, DUBs exert multifaceted regulatory effects on the PI3K/Akt/mTOR signaling pathway. Their interactions with key components like PTEN, Akt, and mTOR complexes crucially influence the pathway's activity, adding layers of control in cellular signaling networks. Understanding these intricate dynamics provides insights into potential therapeutic interventions for diseases characterized by aberrant PI3K/Akt/mTOR signaling.

### 3.4 Wnt/β‐catenin signaling pathway

The Wnt/β‐catenin signaling pathway is crucial for embryonic development and the maintenance of adult tissue homeostasis. Dysregulation of this pathway is implicated in numerous cancers.^102^ The pathway is initiated when Wnt proteins bind to the Frizzled receptor and the LRP5/6 coreceptor on the cell surface. This interaction inhibits the destruction complex, which is composed of Axin, Adenomatous Polyposis Coli (APC), Glycogen Synthase Kinase‐3β (GSK‐3β), and Casein Kinase 1 (CK1). The inhibition of this complex leads to the stabilization and accumulation of β‐catenin in the cytoplasm. Subsequently, β‐catenin translocates to the nucleus, where it forms a complex with T‐cell factor/lymphoid enhancer factor (TCF/LEF) transcription factors, driving the expression of target genes that promote cell proliferation and survival^103^, ^104^ (Figure 3B).

Central to the regulation of the Wnt/β‐catenin pathway is the control of β‐catenin levels and activity. This regulation is achieved through a balance between ubiquitination and deubiquitination processes, where DUBs play pivotal roles. One of the primary regulatory nodes of the Wnt/β‐catenin pathway is the stabilization of β‐catenin. In the absence of Wnt signaling, β‐catenin is targeted for ubiquitination and proteasomal degradation by the destruction complex. Upon Wnt stimulation, this complex is inhibited, allowing β‐catenin to accumulate in the cytoplasm and subsequently translocate to the nucleus. DUBs such as USP5 and USP9X directly interact with β‐catenin to remove inhibitory ubiquitin chains, thereby stabilizing β‐catenin. USP5 deubiquitinates β‐catenin, preventing its degradation and facilitating its nuclear accumulation. Elevated expression of USP5 is associated with larger tumor sizes and poorer differentiation in non‐small cell lung cancer (NSCLC), underscoring its role in tumor progression.^105^ Similarly, USP9X stabilizes β‐catenin by removing K48‐linked polyubiquitin chains, promoting β‐catenin's nuclear translocation and activation of downstream oncogenic targets such as c‐Myc and cyclin D1. Higher levels of USP9X correlate with worse prognosis in high‐grade gliomas.^106^, ^107^

The destruction complex itself is another critical target for DUB regulation. Components of this complex, such as Axin and APC, are essential for β‐catenin degradation. USP15, for instance, targets β‐catenin through the APC complex, stabilizing the destruction complex and facilitating the degradation of β‐catenin.^108^ This action highlights USP15's role as a tumor suppressor by preventing the overactivation of Wnt signaling and subsequent tumor formation.

In addition, cellular stress conditions, such as DNA damage, can hijack the normal regulation of β‐catenin. OTULIN, for example, is phosphorylated at Tyr56 following DNA damage, enhancing its association with β‐catenin.^109^ This stabilized binding prevents β‐catenin degradation and enhances its nuclear accumulation to maintain cell survival pathways under stress conditions. Moreover, OTUD7B exemplifies a nonenzymatic regulatory mechanism in the Wnt/β‐catenin pathway. Instead of deubiquitinating β‐catenin directly, OTUD7B interacts with LEF1, promoting its nuclear localization and enhancing its interaction with β‐catenin.^110^ This interaction underscores the importance of spatial dynamics in the regulation of β‐catenin's transcriptional activity.

Conversely, certain DUBs act to suppress Wnt/β‐catenin signaling. A20 functions in this capacity by modulating the ubiquitination state of RIPK4, a kinase involved in Wnt signaling.^111^ By destabilizing RIPK4, A20 diminishes downstream signaling, providing a negative feedback mechanism to prevent excessive activation of the Wnt pathway.

Given the centrality of β‐catenin regulation in the Wnt pathway, DUBs represent potent therapeutic targets. Targeting specific DUBs could provide a means to modulate β‐catenin levels and activity, offering a strategy to intervene in cancers driven by aberrant Wnt signaling. In sum, the regulation of the Wnt/β‐catenin signaling pathway by DUBs is multifaceted and context‐dependent. DUBs influence various aspects of the pathway, from stabilizing β‐catenin to modulating the activity of destruction complex components and interacting with key transcription factors. Understanding these intricate regulatory mechanisms provides valuable insights into potential therapeutic targets for diseases characterized by dysregulated Wnt signaling, particularly cancer. Future research focused on the development of DUB inhibitors or modulators holds promise for more effective cancer treatments.

### 3.5 Hippo signaling pathway

The Hippo signaling pathway is a critical regulator of cell proliferation, apoptosis, and organ size, with its core components, including the MST1/2 kinases, LATS1/2 kinases, and the transcription coactivators YAP/TAZ. DUBs are essential modulators within this pathway, influencing the stability and activity of its key components^112^ (Figure 3B).

Central to Hippo signaling is the regulation of YAP/TAZ, which activate genes linked to cell growth and survival upon nuclear translocation. DUBs like OTUB1 and OTUB2 prevent YAP degradation, thus sustaining its activity. OTUB1 specifically removes K48‐linked ubiquitin chains from YAP, facilitating its nuclear accumulation.^113^ Additionally, poly‐SUMOylated OTUB2 enhances YAP/TAZ signaling, highlighting the importance of posttranslational modifications.^114^

USP1 and USP19 further exemplify the regulation of YAP/TAZ stability. USP1 focuses on suppressing K11‐linked polyubiquitination of TAZ, linked to the progression of HCC, while USP19 targets K48‐ and K11‐linked chains on YAP, maintaining its activity in cancer.^115^, ^116^

The activity of LATS1/2 kinases, pivotal for phosphorylating and inhibiting YAP/TAZ, is also modulated by DUBs. YOD1 indirectly enhances YAP/TAZ activity by stabilizing ITCH, an E3 ligase, consequently reducing LATS‐mediated phosphorylation of YAP/TAZ.^117^ This demonstrates how DUBs can regulate upstream components to control pathway outputs. USP9X plays a dual role by interacting with both YAP/TAZ and LATS2. Its absence leads to heightened YAP activity and oncogenesis, highlighting its complex involvement in both promoting and inhibiting Hippo pathway activity depending on context.^118^, ^119^

Beyond direct protein stabilization, DUBs like USP47 influence wider cellular processes by modulating proteins such as NLRP3, impacting inflammation and immunity.^120^ This action potentially affects Hippo pathway signaling indirectly, demonstrating the extensive reach of DUB activity across cell regulatory networks.

The dynamic interplay between DUBs and the Hippo signaling pathway is crucial for maintaining cellular homeostasis. By precision‐tuning ubiquitination processes, DUBs offer potential pathways for targeted therapeutic strategies. Continued investigation into DUB mechanisms within Hippo signaling will further illuminate their broader implications in health and disease management.

### 3.6 TGF‐β signaling pathway

The TGF‐β signaling pathway plays a crucial role in regulating a wide array of cellular processes, including proliferation, differentiation, apoptosis, and extracellular matrix production. Dysregulation of this pathway is implicated in various pathologies, particularly cancer.^121^ Central to the TGF‐β signaling cascade is the activation of receptor serine/threonine kinases, which then phosphorylate receptor‐associated Smads (R‐Smads). These R‐Smads form complexes with Co‐Smads and translocate to the nucleus to regulate the transcription of target genes. DUBs are central to maintaining the balance of this pathway by modulating the ubiquitination status of key signaling components^122^ (Figure 3B).

At the heart of TGF‐β signaling are the type I and type II receptors (TGF‐βRI and TGF‐βRII), which are activated upon ligand binding. The stability and availability of these receptors are crucial for proper signal transduction. UCHL1 has been shown to protect TGF‐βRI from degradation, enhancing the overall signaling strength and downstream impact. By preventing the ubiquitination of TGF‐βRI, UCHL1 facilitates sustained receptor presence on the cell surface, promoting continuous signaling cascades that are vital for processes such as tissue repair and immune response.^123^

Similarly, USP15 plays a significant role in stabilizing TGF‐βRI by deubiquitinating it within the SMAD7–SMURF2 complex. This action prevents receptor degradation and ensures ongoing signaling.^124^ This stabilization is crucial for numerous physiological responses, including cellular proliferation and differentiation. The involvement of USP15 highlights how DUBs can maintain receptor integrity, thereby modulating signal intensity and duration.

Central to TGF‐β signaling are the SMAD proteins, which translocate to the nucleus to regulate gene expression once activated by receptor‐mediated phosphorylation. DUBs exert significant control over these proteins by modulating their ubiquitination status, thus influencing signaling efficiency. OTUD1 has a dual regulatory effect by modulating the ubiquitination of both SMAD3 and SMAD7. It enhances the formation of the SMAD3/SMAD4 complex by removing ubiquitin chains from SMAD3, facilitating its nuclear translocation and transcriptional activity.^125^ Concurrently, OTUD1 stabilizes SMAD7, a negative regulator of the pathway, by cleaving its ubiquitin chains.^126^ This dual action enables OTUD1 to fine‐tune the pathway, promoting or inhibiting signal propagation as needed, thus contributing to cellular homeostasis.

USP26 also impacts SMAD7's stability. By limiting its degradation, USP26 contributes to the attenuation of TGF‐β signaling, providing a check against excessive pathway activation.^127^ This regulatory feedback loop involving USP26 ensures that cells can swiftly respond to changes in signaling demands, maintaining equilibrium in cellular responses.

The influence of DUBs extends beyond immediate pathway components to affect broader developmental and pathological outcomes. USP9X, for example, deubiquitinates SMAD4, a critical mediator in the TGF‐β pathway.^128^ This regulation is essential during embryonic development, particularly in mesoderm formation. The dysfunction of USP9X leads to impaired neurodevelopment and associated disorders, emphasizing its role in balancing signaling during critical development phases.^129^

Understanding how DUBs influence the TGF‐β pathway provides insights into their potential as therapeutic targets. Given their role in fine‐tuning critical pathway components, targeting specific DUBs offers a strategy for modulating TGF‐β signaling in diseases characterized by its dysregulation. The dual roles of DUBs in promoting and inhibiting signaling suggest that selective modulation can restore balance in disorders such as cancer and fibrosis.

These pathways significantly influence various biological processes such as cell proliferation, differentiation, and apoptosis, further revealing the complex role and potential therapeutic value of the DUB family in tumor biology. Therefore, studying the specific functions and regulatory mechanisms of the DUB family in these pathways can enhance our understanding of cancer pathogenesis and may provide a theoretical basis for developing new anticancer strategies.

## 4 THE ROLE OF DUBs IN CANCER

In the field of cancer research, the abnormal expression of DUB family members is closely associated with various common cancers. The function of DUBs in cancer is complex, as they can promote tumor development in certain contexts while exhibiting inhibitory effects in others. This duality makes DUBs potential targets for cancer treatment, particularly in the development of therapeutic strategies targeting specific DUBs. This chapter explores the multifaceted roles of DUBs in tumor initiation, progression, cell death, DNA damage repair, drug resistance, energy metabolism, immune response, and tumor microenvironment (TME) regulation (Table 1).

**TABLE 1:** The roles of DUBs in cancers.

DUB	Effect	Target	Consequences	References
USP1	Activation	CDK5, ULK1, EZH2, CHK1, FANCD2, FANCI	Promote proliferation, metastasis, cancer cell stemness, and regulate DDR	^130^, ^131^, ^132^, ^133^, ^134^, ^135^
USP2	Activation	TGFBR1, PD‐L1, MDM2	Promote proliferation, metastasis and immune evasion, evade apoptosis	^136^, ^137^, ^138^
USP4	Activation	CypA, TAK1, TGFR‐1, β‐catenin	Promote proliferation and metastasis, decrease sensitivity to chemotherapy	^84^, ^139^, ^140^, ^141^
USP5	Activation	c‐Maf, β‐catenin, TUFM, LSH, Beclin1, PD‐L1, HIF2α	Regulate cell death and immune escape, promote proliferation and metastasis	^142^, ^143^, ^144^, ^145^, ^146^, ^147^, ^148^
USP7	Activation	c‐Abl, ARF, YY1, Mdm2, PD‐L1, M1/M2	Antagonize p53‐involved tumor suppression regulations, promote tumor metastasis, decrease drug sensitivity, regulate DDR	^149^, ^150^, ^151^, ^152^, ^153^, ^154^
CYLD	Inhibition	NoxO1, Dvl, p53, c‐Jun, c‐Fox	Suppress tumorigenesis and metastasis, promote apoptosis, regulate DDR	^155^, ^156^, ^157^, ^158^
USP8	Activation	MT1, SLC7A11, SQSTM1, TβRII	Regulate cell death, promote proliferation and metastasis, inhibit immunotherapy	^159^, ^160^, ^161^
USP9X	Activation	Ets‐1, MCL1, YAP1, XIAP, SMAD4	Promote proliferation and metastasis, evade apoptosis.	^119^, ^128^, ^162^, ^163^, ^164^
USP9X	Inhibition	FBW7, CLASPIN	Maintain DNA replication fork stability and inhibit proliferation	^165^, ^166^
USP10	Inhibition	KLF4	Promote the expression of tumor suppressor genes, inhibit cancer occurrence and metastasis	^167^
USP11	Activation	ERα, HIF‐1α, H2A, H2B, γH2AX	Influence apoptosis, participate in DDR and promote cell survival	^168^, ^169^, ^170^, ^171^
USP11	Inhibition	XPC	Maintain NER capacity, participate in DDR	^172^
USP12	Activation	HMGB1, AR, Mdm2	Regulate autophagy, promote cell survival	^173^, ^174^, ^175^
USP13	Activation	c‐Myc, MCL1, RAP80, ACLY, OGDH	Promote proliferation and metabolism, chemotherapy resistance	^176^, ^177^, ^178^, ^179^
USP14	Activation	Dvl, Vimentin, Aurora B, AR, PFKL	Promote proliferation and metastasis, prevent apoptosis and regulate DDR	^180^, ^181^, ^182^, ^183^, ^184^
USP15	Activation	P65, Mdm2, BARD1, TβR‐I, TOP2α	Promote metastasis and inhibit antitumor immunoresponses	^124^, ^185^, ^186^, ^187^, ^188^
USP15	Inhibition	Keap1, p53, IRS‐2	Inhibit tumorigenesis and decrease chemo‐resistance	^189^, ^190^, ^191^
USP17	Activation	CDC25A, Geminin, Slug, Twist, Snail, HAS2, SMAD4, BRD4	Promote tumorigenesis and metastasis, prevent apoptosis	^192^, ^193^, ^194^, ^195^, ^196^, ^197^, ^198^
USP18	Activation	ZEB1, RARα, BCL2L1, KRAS	Promote tumorigenesis and metastasis, prevent apoptosis	^199^, ^200^, ^201^, ^202^
USP20	Activation	β‐catenin, SNAI2	Promote tumorigenesis	^203^, ^204^
USP20	Inhibition	Claspin, TRAF6, Tax	Inhibit tumorigenesis	^205^
USP22	Activation	CCNB1, CCND1, c‐Myc, FBP1, H2A, EGFR, KDM1A, SIRT1, PD‐L1, PPARγ	Promote proliferation, metastasis and cancer stemness, drug resistance, reduce sensitivity to immune therapy, regulate γH2AX‐mediated DDR	^93^, ^206^, ^207^, ^208^, ^209^, ^210^, ^211^, ^212^, ^213^, ^214^
USP22	Inhibition	PU.1	Antidifferentiation	^215^
USP25	Activation	HIF‐1α	Promote metabolic reprogramming and survival	^216^
USP26	Activation	AR, Snail	Promote proliferation and metastasis	^217^, ^218^
USP26	Inhibition	SMAD7	Inhibit metastasis	^127^
USP27	Activation	Cyclin E	Promote cell cycle progression and tumorigenesis	^219^
USP28	Activation	c‐Myc, Fbw7, LIN28A, LSD1, c‐Jun	stabilize oncogenic factors, promote tumorigenesis and metastasis	^220^, ^221^, ^222^, ^223^
USP28	Inhibition	p53	Induce cell cycle arrest in cancer cells	^224^
USP29	Activation	Snail1, Claspin	Promote tumorigenesis and metastasis	^225^, ^226^
USP29	Inhibition	P53	Induces apoptosis	^227^
USP30	Activation	DRP1, TOM20	Promote migration, evade apoptosis	^228^, ^229^
USP35	Activation	ABHD17C, FPN	Promote proliferation, migration, and invasion, reduce chemosensitivity	^230^, ^231^
USP35	Inhibition	ABIN‐2	Inhibit cell proliferation and NF‐κB activation	^232^
USP36	Activation	c‐Myc, DHX33, H2B, ERα	Promote tumorigenesis and proliferation	^233^, ^234^, ^235^, ^236^
USP37	Activation	c‐Myc, RARA, Gli1, Snail, 14‐3‐3γ	Promote proliferation and metastasis	^237^, ^238^, ^239^, ^240^, ^241^
USP37	Inhibition	p27	Inhibit proliferation	^242^
USP38	Activation	KLF5, LSD1	Promote proliferation and enhance treatment resistance	^243^, ^244^
USP39	Activation	ZEB1, β‐catenin	Promote proliferation and metastasis	^245^, ^246^
USP48		TRAF2, Gli1	Reduce E‐cadherin‐mediated adherens junctions, promote proliferation and metastasis	^247^, ^248^
OTUB1	Activation	RAS, Snail, β‐Catenin, RhoA/p53, CHK1, C‐Maf, SLC7A11, OXPHOS, TGFBI, NDUFS2, PD‐L1	Promote cell proliferation, metastasis, and invasion, modulate DDR, promote immune escape, and enhance therapeutic resistance	^249^, ^250^, ^251^, ^252^, ^253^, ^254^, ^255^
OTUB1	Inhibition	CNN6, P27, p53	Affect tumor cell cycle arrest, inhibit tumor cell proliferation, survival, and migration	^256^, ^257^, ^258^
OTUB2	Activation	U2AF2, PJA1, p65, YAP/TAZ, γH2AX, PKM2	Regulate signal transduction and promote cell proliferation, migration, and invasion	^259^, ^260^, ^261^, ^262^, ^263^, ^264^
OTUB2	Inhibition	STAT1, RNF8, SNX29P2	Regulate glycolysis and DDR, inhibit proliferation	^265^, ^266^, ^267^
OTUD1	Activation	BALF1, KEAP1, Nrf2, YAP, FGL1	Activate the NF‐κB signaling pathway to promote proliferation, migration, and antiapoptosis	^268^, ^269^, ^270^, ^271^
OTUD1	Inhibition	SMAD7, KLF4, FHL1, AIF, MCL1, PTEN, P16, YAP1, IREB2, Bim	Trigger apoptosis, inhibit proliferation, migration and invasion, enhance drug sensitivity	^126^, ^272^, ^273^, ^274^, ^275^, ^276^, ^277^, ^278^, ^279^, ^280^
OTUD2	Activation	CDK1, NEDD4, YAP	Promote proliferation and migration, affect TME	^281^, ^282^, ^283^
OTUD2	Inhibition	TRIM33	Inhibit growth, invasion, and metastasis	^284^
OTUD4	Activation	CDK1, Snail1, IRTKS	Promote proliferation, migration, and invasion	^285^, ^286^, ^287^
OTUD4	Inhibition	RBM47, GSDME, CD73, ATM/CHK2/P53	Induce cell death, inhibit DDR and enhance chemosensitivity	^288^, ^289^, ^290^, ^291^
OTUD5	Activation	YAP, SLC38A1, RNF186	Promote proliferation, invasion, and migration	^292^, ^293^, ^294^
OTUD5	Inhibition	PTEN, TRIM25FACT, cGAS/STING	Suppress proliferation, invasion, and migration	^294^, ^295^, ^296^, ^297^, ^298^
OTUD7B	Activation	ERα, AKT/VEGF, N1ICD, APC/C, PIK3C3	Activate autophagy, promote proliferation, metastasis, and invasion	^299^, ^300^, ^301^, ^302^, ^303^
OTUD7B	Inhibition	Smac	Inhibit proliferation and migration	^304^
A20	Activation	HSP70, Snail1, Akt, PFKL, PD‐L1	Protects against cell death and promotes invasion and metastasis	^305^, ^306^, ^307^, ^308^, ^309^
A20	Inhibition	Twist1, ERK, mTORC2	Inhibit proliferation and migration	^310^, ^311^, ^312^
OTULIN	Activation	β‐catenin	Increases cancer cell resistance to genotoxic drug treatments	^109^
OTULIN	Inhibition	mTOR, FADD	Prevents liver cancer from occurring	^313^, ^314^
UCHL1	Activation	HIF‐1α, TβRI, SMAD2	Promote metastasis	^123^, ^315^
UCHL1	Inhibition	NOXA, p53	Promote apoptosis and enhance chemosensitivity	^316^, ^317^
UCHL3	Activation	TDP1, AhR	Drug resistance	^318^, ^319^
BAP1	Activation	KLF5, MCRS1	Maintain gene stability and promote proliferation and migration	^320^, ^321^
BAP1	Inhibition	H2A, γ‐tubulin, MAFF	Maintain chromosomal stability, induce cell apoptosis	^322^, ^323^, ^324^
PMSD14	Activation	E2F1, ALK2, RAP80	Promote proliferation and migration and enhance treatment resistance	^325^, ^326^, ^327^

Abbreviations: DDR, DNA damage repair; NER, nucleotide excision repair; TME, tumor microenvironment.

### 4.1 DUBs and tumor development

**FIGURE 4:**
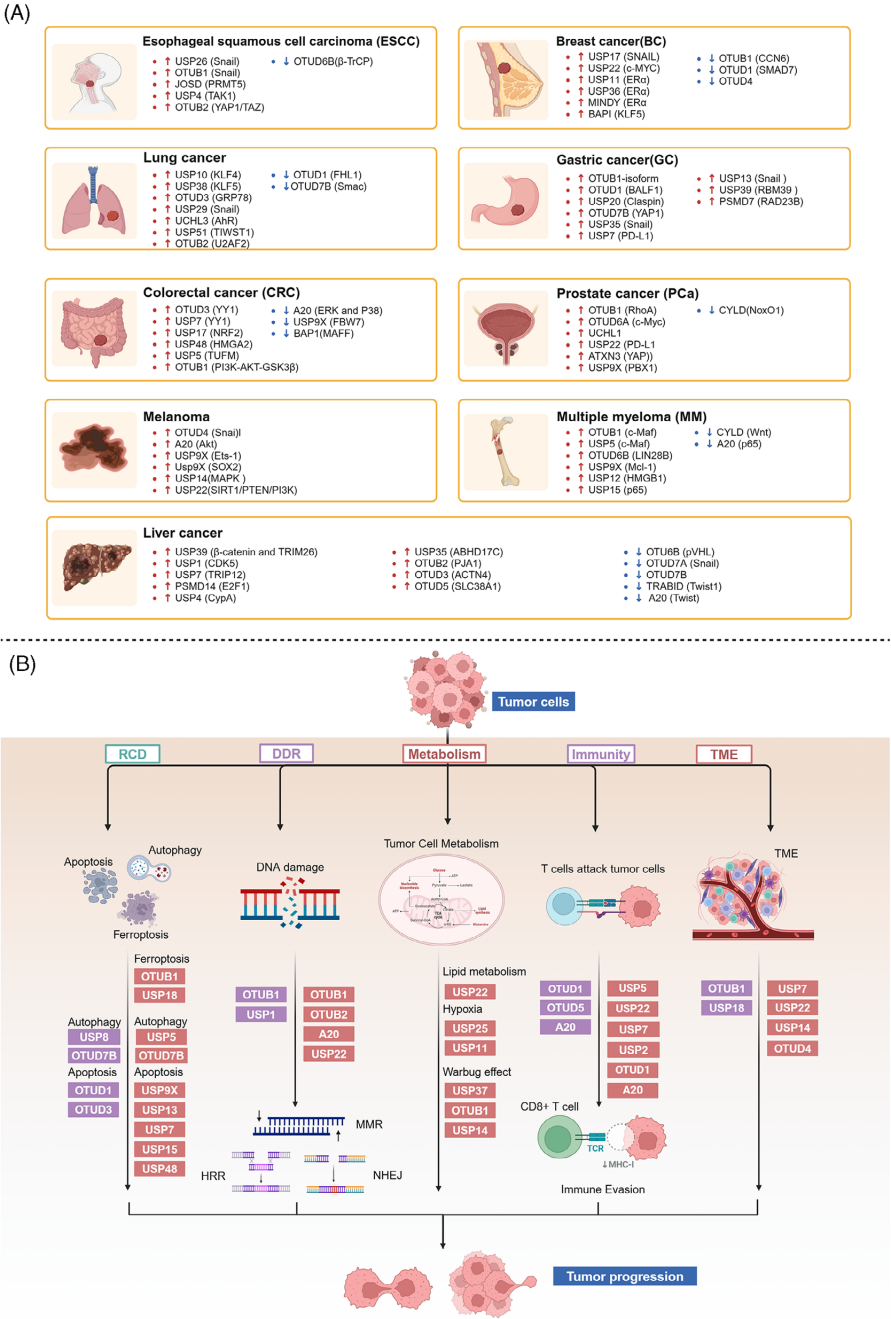
Dual roles of the DUB family in cancer: tumor suppressor and tumor promoter function. (A) This panel provides an overview of how the DUB family inhibits or promotes the development of various common cancers, including breast, liver, and lung cancers. Each type of cancer is described with specific DUB family members and their roles. For instance, in breast cancer (BC), OTUD3 stabilizes PTEN, thereby inhibiting tumor growth, while OTUB1 stabilizes HIF‐1α, promoting tumor progression. (B) This panel presents a schematic illustration of how the DUB family affects cancer progression through the dual regulation of tumor cell death, DDR, energy metabolism, immunity, and the TME. Purple boxes represent DUBs that act as tumor suppressors, while orange boxes represent DUBs that act as oncogenes. For example, in the DDR, OTUB1 and OTUB4 are involved in the repair of DNA damage, influencing HRR and NHEJ in tumor cells.

Increasing evidence suggests that DUBs play a significant role in tumor initiation and progression, especially in common cancers such as breast cancer, liver cancer, lung cancer, and CRC. The impact of DUBs is not singular or fixed; rather, they influence key processes such as tumor cell growth, proliferation, migration, and invasion through their deubiquitination activity. This effect is primarily achieved by regulating the stability of key proteins and their interactions with known ubiquitinases (Figure 4A).

#### 4.1.1 Breast cancer

Breast cancer, a diverse and multifaceted malignancy, stands as a leading cause of cancer‐related mortality among women worldwide.^328^ DUBs have emerged as critical regulators in the oncogenic processes of breast cancer through various mechanisms, including protein stabilization, signal transduction, and modulation of apoptosis. This section will focus on several representative DUBs that highlight their significant roles in breast cancer development and progression.

Notably, USP17 influences breast cancer metastasis through its role in epithelial–mesenchymal transition (EMT). EMT is essential for cancer cell migration and invasion, with USP17 stabilizing the transcription factor SNAIL1 by deubiquitination. This stabilization promotes EMT, thereby enhancing the metastatic potential of breast cancer cells, making USP17 a promising target to impede cancer dissemination.^329^

Similarly, USP22 significantly impacts cell proliferation and tumor growth by regulating the oncogene c‐Myc. c‐Myc drives the expression of genes involved in cell cycle progression and proliferation, and USP22 stabilizes c‐Myc through deubiquitination, leading to increased tumorigenic activity.^208^ Targeting USP22 could therefore destabilize c‐Myc, representing a strategic approach to inhibit breast cancer growth.

In ERα‐positive breast cancer, USP11 is crucial. ERα, a hormone receptor driving cell proliferation and survival in response to estrogen, is stabilized by USP11. High levels of USP11 correlate with poor prognosis as it enhances ERα signaling pathways, promoting tumor growth. Disrupting USP11 could offer therapeutic benefits by impairing ERα‐driven cancer progression.^168^

Building on the modulation of ERα signaling, MINDY1 and USP36 also play essential roles. MINDY1 prevents the degradation of ERα by inhibiting its polyubiquitination, leading to growth inhibition when depleted.^330^ On the other hand, USP36 enhances ERα stability by reducing its ubiquitination, contributing to resistance against hormone therapies like tamoxifen.^236^ These DUBs are vital for maintaining ERα function, and their inhibition could help overcome endocrine resistance.

Moreover, OTUB1 and OTUD1 act as tumor suppressors in breast cancer. OTUB1 stabilizes CCN6, reducing cell proliferation and invasiveness.^256^ Meanwhile, OTUD1 inhibits metastasis by deubiquitinating SMAD7, countering the prometastatic effects of TGF‐β signaling.^126^ These enzymes play crucial roles in maintaining cellular integrity and preventing cancer spread, highlighting their therapeutic potential.

In summary, DUBs play multifaceted roles in breast cancer progression and therapy resistance. By stabilizing key regulatory proteins and modulating signaling pathways, DUBs significantly influence the tumorigenic processes. Understanding the specific mechanisms by which these DUBs operate offers valuable insights into potential therapeutic targets for breast cancer treatment. By targeting specific DUBs, it may be possible to disrupt critical pathways involved in breast cancer progression, providing new avenues for effective treatments.

#### 4.1.2 Lung cancer

Lung cancer, a leading cause of cancer‐related mortality worldwide, presents significant challenges due to its aggressive nature and complex biological mechanisms.^331^ DUBs play crucial roles in lung cancer by modulating protein stability and influencing key signaling pathways, thereby contributing to tumor progression, metastasis, and resistance to therapy.

One of the critical aspects of DUB function in lung cancer involves the regulation of KLF family members, which are key transcription factors involved in cellular differentiation and proliferation. USP10 stabilizes KLF4 by preventing its degradation, thus promoting cell growth and survival. Higher levels of USP10 and consequently KLF4 are associated with enhanced tumorigenesis and poorer prognosis in lung cancer patients, highlighting the therapeutic potential of targeting USP10^167^. Similarly, USP38 enhances the stability of KLF5 through deubiquitination, with PIAS1‐mediated SUMOylation augmenting this effect. KLF5 promotes cell proliferation and survival, and its stabilization by USP38 underscores its importance in lung adenocarcinoma progression.^243^ This interaction provides valuable insights into potential therapeutic interventions aimed at disrupting these pathways.

Another significant DUB, OTUD3, plays a role in lung cancer growth and metastasis by stabilizing the glucose regulatory protein GRP78. GRP78 is essential for cancer cell survival under stress conditions, indicating OTUD3's involvement in promoting tumor resilience and progression. This effect is modulated by the E3 ubiquitin ligase CHIP, which negatively regulates the GRP78 stabilization process, suggesting a potential therapeutic balance between DUB and ubiquitin ligase activities.^332^

Additionally, USP29 enhances lung adenocarcinoma cell tumorigenesis by stabilizing Snail1, a transcription factor critical for maintaining aggressive cancer phenotypes. Chemotherapy‐induced oxidative stress increases USP29 expression, which in turn enhances Snail1 stability and contributes to drug resistance and poor prognosis.^225^ Targeting USP29 could, therefore, offer a novel therapeutic approach to counteract chemotherapy resistance and tumor progression in lung cancer.

In contrast, OTUD1 acts as a tumor suppressor in lung cancer. It stabilizes FHL1, thereby inhibiting lung adenocarcinoma cell proliferation, migration, and invasion.^273^ The tumor‐suppressive effect of OTUD1 highlights its potential as a therapeutic target to restrain lung cancer progression by preventing the spread and growth of malignant cells.

In summary, DUBs play diverse and significant roles in regulating lung cancer development, progression, and therapy resistance through their effects on protein stability and signaling pathways. These enzymes demonstrate intricate regulatory mechanisms involved in lung carcinogenesis. Understanding these functions provides valuable insights into potential therapeutic strategies aimed at disrupting key pathways in lung cancer. Future research is essential to fully elucidate the mechanisms of these DUBs and to develop targeted therapies that enhance the clinical management of lung cancer.

#### 4.1.3 Liver cancer

Liver cancer, predominantly hepatocellular carcinoma (HCC), arises from chronic conditions such as hepatitis virus infection, alcoholic liver disease, and nonalcoholic fatty liver disease, leading to significant health issues and high mortality rates.^333^ HCC, as the most prevalent form of liver cancer, constitutes a major portion of cases and is known for its aggressive nature and complex pathogenesis. This section focuses on the crucial roles of DUBs in HCC, elucidating how these enzymes contribute to cancer progression through their regulatory functions in ubiquitination processes.

USP39 prominently promotes HCC cell proliferation and migration by deubiquitinating β‐catenin, a critical factor in the Wnt/β‐catenin signaling pathway. The stabilization of β‐catenin enhances its contribution to HCC progression.^246^ Furthermore, USP39 influences the pre‐mRNA splicing and maturation of TRIM26, which, though expressed at low levels in HCC tissues, inhibits cell proliferation and migration by mediating the ubiquitination and degradation of ZEB1.^245^ This antagonistic relationship between USP39 and TRIM26 in regulating ZEB1 stability underlines their combined impact on HCC development.

In the realm of mitochondrial function, USP1 plays a significant role by enhancing the phosphorylation of Drp1 at Ser616 through the deubiquitination and stabilization of cyclin‐dependent kinase 5 (CDK5), normally targeted for degradation by the E3 ligase NEDD4L.^130^ This USP1/CDK5 axis is notably activated in HCC tissues and is correlated with poorer patient outcomes, indicating its contribution to tumorigenesis.

USP7 is another vital regulator in HCC, promoting cell growth by stabilizing thyroid hormone receptor‐interacting protein 12. This stabilization results in the perpetual ubiquitination of p14(ARF), thereby facilitating HCC cell proliferation and survival. Clinically, USP7 overexpression is associated with more aggressive tumor characteristics, such as larger size, poor differentiation, elevated alpha‐fetoprotein levels, and microvascular invasion, reinforcing its key role in HCC malignancy.^150^ In addition, PSMD14 contributes to HCC progression by stabilizing E2F1 through direct binding and deubiquitination. This stabilization activates E2F1's downstream prosurvival signals, including the upregulation of survivin and FOXM1, which collectively enhance the tumor growth capacity of HCC cells.^325^

The OTU family members, particularly OTUD7B and OTUD7A, exhibit inhibitory effects on HCC. OTUD7B reduces liver cancer cell adhesion and migration, potentially via the induction of the EMT process, thereby hindering the invasion of cancer cells.^334^ OTUD7A works through Snail1 to suppress the proliferation, migration, and invasion of liver cancer cells, underscoring their tumor‐suppressive roles.^335^

Overall, DUBs are integral to HCC progression regulation by influencing cell cycle, survival, mitochondrial dynamics, and oncogenic signaling pathways. They achieve this through intricate protein stability and signaling modulation mechanisms, highlighting their potential as therapeutic targets in HCC. The distinct, and occasionally antagonistic, roles of DUBs offer profound insights into the molecular complexities of HCC.

#### 4.1.4 Colorectal cancer

CRC ranks among the most prevalent malignant tumors globally, with its incidence largely driven by genetic factors, dietary habits, lifestyle choices, and aging. The profound impact of CRC on global health necessitates a detailed exploration of its molecular mechanisms.^336^ This section delves into the crucial roles of DUBs in CRC, highlighting how these enzymes modulate key regulatory pathways and contribute to cancer progression and therapeutic resistance.

OTUD3 and USP7 are key players in promoting CRC by regulating the transcription factor YY1. OTUD3 enhances CRC cell growth by deubiquitinating and stabilizing YY1. Phosphorylation of OTUD3 significantly augments its ability to stabilize YY1, and high levels of YY1, which are frequently observed in CRC, suggest its pivotal role in CRC cell proliferation and metastasis.^46^ Similarly, USP7 stabilizes YY1 by interfering with its K63‐linked ubiquitination and subsequent proteasome‐mediated degradation. Stabilized YY1 activates TRIAP1 and inactivates LC3B, thereby promoting CRC cell growth and metastasis,^151^ highlighting a synergistic effect of OTUD3 and USP7 on YY1 stabilization in CRC progression.

Additionally, USP17 promotes chemotherapy resistance in CRC through the NRF2–ARE pathway. USP17 enhances NRF2 stability and its transcriptional activity by decreasing K48‐linked ubiquitination of NRF2. Overexpression of USP17 induces NRF2‐dependent resistance to chemotherapy in colon cancer cell lines, marking the dual role of USP17 in both tumor progression and drug resistance.^337^

On the other hand, certain DUBs such as A20 and USP9X function as tumor suppressors in CRC. A20 inhibits CRC progression by reducing the activation of ERK and P38 signaling pathways and by mitigating TNFα‐induced chemokine production, thus controlling inflammation and oncogenic signaling.^311^ This tumor suppressive role of A20 highlights its importance in maintaining cellular homeostasis and inhibiting cancer progression.

USP9X stabilizes the tumor suppressor FBW7, which targets oncoproteins like c‐MYC for degradation. USP9X antagonizes the ubiquitination of FBW7, and its deletion leads to the destabilization of FBW7, resulting in increased levels of c‐MYC and enhanced tumor proliferation. The regulatory axis of USP9X–FBW7 is crucial for maintaining low levels of oncogenic c‐MYC, thereby suppressing CRC progression.^165^

In summary, DUBs exhibit diverse and critical roles in CRC by regulating key proteins involved in cell growth, survival, metastasis, and chemoresistance. The intricate regulatory mechanisms of DUBs in CRC highlight the complexity of ubiquitination processes in cancer biology and the need for continued research to fully harness their therapeutic potential.

#### 4.1.5 Squamous cell carcinoma

Squamous cell carcinoma (SCC) encompasses various forms of epithelial malignancies arising in different anatomical regions, including the skin, oral cavity, esophagus, cervix, and vagina. This heterogeneity underscores the complexity of SCC and the necessity for detailed molecular analyses to understand its pathogenesis.^338^ In this context, DUBs have emerged as significant regulators in the development and progression of SCC, influencing processes such as cell proliferation, migration, invasion, and chemoresistance.

In esophageal squamous cell carcinoma (ESCC), DUBs such as USP26 and OTUB1 are significant regulators of the EMT, a process crucial for cancer metastasis driven by the transcription factor Snail. USP26 stabilizes Snail by deubiquitination, enhancing its role in promoting cell migration and invasion. High Snail expression is associated with poor prognosis and increased metastatic potential in ESCC.^218^ Similarly, OTUB1 stabilizes Snail, further facilitating the invasive characteristics of ESCC cells and underscoring its pivotal role in EMT and cancer progression.^250^

JOSD2 is another important DUB in ESCC, markedly upregulated in cancer cells compared with normal esophageal cells. JOSD2 knockdown significantly reduces cell proliferation, colony formation, drug resistance, and migration. Conversely, JOSD2 overexpression enhances these malignant traits, highlighting its critical role in ESCC progression.^339^ Its regulatory effect on key pathways emphasizes JOSD2 as a potential therapeutic target.

OTUB1 also promotes malignant behaviors such as tumor cell proliferation, migration, and invasion in oral squamous cell carcinoma (OSCC) and head and neck squamous cell carcinoma (HNSCC), demonstrating its universal importance in various types of SCC.^340^, ^341^ Furthermore, YOD1 can inhibit the activation of the ERK/β‐catenin pathway by targeting the ubiquitination of TRIM33, thereby inhibiting the growth, invasion, and metastasis of HNSCC.^284^

In cervical squamous cell carcinoma (CSCC), SIRT7 is crucial for promoting cell proliferation and autophagy through its interaction with USP39. SIRT7 deacetylates and stabilizes USP39, which is upregulated in CSCC tissues. Silencing USP39 results in inhibited cell growth, emphasizing its tumor‐promoting role.^342^ The SIRT7–USP39 interaction reveals a unique regulatory mechanism that drives CSCC progression, making it a potential target for therapeutic strategies.

In conclusion, DUBs are integral to SCC progression by regulating crucial proteins involved in EMT, cell proliferation, and survival. Understanding these mechanisms offers valuable insights into targeted therapies for inhibiting SCC progression and metastasis, underscoring the complexity and potential therapeutic value of DUBs in SCC.

#### 4.1.6 Other cancers

This section elaborates on the functions and potential pathological mechanisms of the OTU family in other cancer types. While some studies have highlighted the significant roles of OTU family members in breast cancer, lung cancer, liver cancer, CRC, and SCC, OTU proteins also play key roles in other cancers such as prostate cancer, multiple myeloma (MM), and melanoma. These studies expand our understanding of the OTU family's functions and provide new targets for treating these deadly cancers.

In MM, DUBs such as USP9X and OTUB1 are pivotal. USP9X prevents the degradation of Mcl‐1 by removing Lys48‐linked poly‐ubiquitin chains, thus enhancing cell survival and correlating with poor clinical outcomes.^163^ Similarly, OTUB1 enhances the stability and transcriptional activity of c‐Maf, further promoting MM cell survival and worsening patient prognosis.^254^

Prostate cancer progression is significantly affected by DUBs like CYLD, OTUB1, and OTUD6A. CYLD destabilizes NoxO1 through promoting ubiquitination, thereby reducing ROS generation and inhibiting tumor growth.^155^ OTUB1 influences disease progression by regulating RhoA activation and p53 expression, while OTUD6A promotes tumor development by stabilizing the oncogenic protein c‐Myc.^252^, ^343^

Melanoma progression involves DUBs such as OTUD4, A20, and USP9X. OTUD4 contributes to chemotherapy resistance, whereas A20 facilitates melanoma progression via the Akt pathway.^286^, ^307^ USP9X enhances the tumorigenic potential of metastatic melanoma by preventing proteasomal degradation of Ets‐1 through deubiquitination, a function reversible through USP9X knockdown or inhibition.^162^

In gastric cancer (GC), DUBs like USP20, OTUB1, and OTUD1 play significant roles. Silencing USP20 reduces Claspin protein levels, which impact GC cell growth.^205^ Both OTUB1 and OTUD1 promote GC progression by stabilizing proteins involved in proliferation and antiapoptotic pathways.^344^ Particularly, OTUD1 deubiquitinates BALF1, increasing Bcl‐2 stability, which in turn enhances cancer cell proliferation and migration^268^

DUBs play dual roles as oncogenes and tumor suppressors in various cancers, highlighting their biochemical versatility and complexity. Despite extensive research, the precise molecular mechanisms and context‐specific functions of many OTU members remain unclear. Future studies should focus on uncovering these context‐dependent roles, their interactions with other signaling pathways, and potential cotargeting opportunities. Additionally, understanding the structural basis for their dual functions could aid in developing selective inhibitors or activators, leading to innovative, precise cancer therapies and overcoming current treatment challenges.

### 4.2 DUBs and tumor cell death

In exploring cancer therapies, the regulation of cell death mechanisms is particularly crucial. The DUBs plays a central role in regulating various cell death pathways. Although apoptosis is the primary mechanism of tumor cell death, increasing research indicates that nonapoptotic forms of cell death, such as ferroptosis, autophagy, and necroptosis, also represent emerging regulatory pathways that inhibit tumor progression. In‐depth studies of these mechanisms not only help understand the survival strategies of tumor cells but may also reveal new therapeutic target (Figure 4B).

#### 4.2.1 Apoptosis

Apoptosis is a programmed cell death mechanism characterized by the orderly breakdown of cellular components, which prevents inflammatory responses. Dysregulation of apoptosis is a hallmark of cancer, enabling tumor cells to evade death and proliferate. DUBs such as USP9X, USP13, and OTUD1 are particularly important in regulating apoptosis through the modulation of Mcl‐1, a prosurvival member of the Bcl‐2 protein family.

Mcl‐1 is integral to the mitochondrial pathway of apoptosis, where it inhibits proapoptotic proteins like Bax and Bak. USP9X prevents Mcl‐1 degradation by removing poly‐ubiquitin chains, enhancing cell survival and contributing to radioresistance in cancer cells.^345^ Similarly, USP13 interacts with and stabilizes Mcl‐1, and its inhibition reduces Mcl‐1 levels, increasing the sensitivity of tumor cells to BH3 mimetic inhibitors.^177^ OTUD1 also impacts Mcl‐1 stability and indirectly affects apoptosis by regulating the nuclear localization of apoptosis‐inducing factor (AIF).^274^

DUBs also modulate apoptosis through the MDM2/p53 pathway, a critical regulatory axis in cell death and tumor suppression. USP7 stabilizes MDM2, leading to p53 degradation and reduced apoptosis. Inhibition of USP7 promotes MDM2 degradation, activates p53 signaling, and induces apoptosis.^152^ USP15 similarly stabilizes MDM2, thus negatively impacting p53 function and T cell activation, suggesting that USP15 inhibition could both enhance tumor cell apoptosis and boost antitumor immunity.^186^ OTUD3 stabilizes p53 by competing with MDM2, promoting p53‐mediated apoptotic signaling in breast cancer cells.^346^ Furthermore, USP48 enhances MDM2 stability by a deubiquitination‐independent mechanism, thereby promoting p53 degradation and apoptosis avoidance.^346^

#### 4.2.2 Nonapoptotic cell death

Nonapoptotic cell death pathways, such as ferroptosis and autophagy, also play significant roles in cancer biology. Ferroptosis is an iron‐dependent form of cell death driven by lipid peroxidation. USP8 regulates ferroptosis by stabilizing O‐GlcNAc transferase, which is essential for cystine import and glutathione biosynthesis. Inhibition of USP8 leads to increased ROS and promotes ferroptosis, thereby suppressing HCC growth.^160^ OTUB1 also affects ferroptosis by regulating the stability of SLC7A11, a key player in cystine import and redox balance.^255^

Autophagy, a cellular degradation process, helps in the removal of damaged organelles and proteins, thus playing a dual role in cancer. USP5 facilitates autophagy by stabilizing Beclin 1 and promoting p53 instability. The inhibition of USP5 not only suppresses autophagy but also imposes a p53‐dependent senescence burden, thereby blocking tumor growth.^146^ USP8 directly interacts with and deubiquitinates SQSTM1, inhibiting its degradation, and controlling autophagic flux.^161^ OTUD7B regulates PIK3C3, either stabilizing it to activate autophagy or promoting its degradation to inhibit autophagy, indicating its context‐dependent roles.^303^, ^347^

Beyond apoptosis, ferroptosis, and autophagy, DUBs like OTUD4 also influence other forms of cell death, such as pyroptosis. OTUD4 promotes cell pyroptosis by stabilizing GSDME, a critical executor of this inflammatory form of cell death, and enhances radiosensitivity.^289^

The regulation of tumor cell death by DUBs is complex and multifaceted, encompassing a range of apoptotic and nonapoptotic pathways. Although significant advances have been made, the precise molecular mechanisms and context‐specific roles of many DUBs remain incompletely understood. Future research should focus on unraveling these mechanisms, exploring DUB interactions with other signaling pathways.

### 4.3 DUBs in DNA damage repair and treatment resistance

DNA damage repair is an essential cellular mechanism that ensures genomic integrity and stability. Dysregulation of these repair pathways, especially those involved in addressing DNA double‐strand breaks (DSBs), is a hallmark of cancer and implicates multiple proteins, heavily relying on ubiquitination processes.^348^ DUBs are pivotal in regulating protein ubiquitination and managing DNA damage repair, thus impacting tumor growth and progression. Furthermore, resistance to drug therapy, radiotherapy, and chemotherapy presents a significant challenge in cancer treatment. This section will explore DNA damage repair mechanisms and their complex relationship with treatment resistance (Figure 4B).

USP1 is critical in the Fanconi anemia (FA) pathway for repairing DNA crosslinks. It deubiquitinates FANCD2 and FANCI, ensuring their function in the S phase of the cell cycle for effective DNA repair.^135^, ^349^ USP1 also deubiquitinates proliferating cell nuclear antigen, influencing the translesion DNA synthesis repair pathway, and regulates checkpoint kinase 1 (CHK1) activity, impacting DDR and cellular differentiation.^133^, ^350^

OTUB2 is pivotal in DSB repair, affecting the choice between homologous recombination (HR) and nonhomologous end joining (NHEJ). By inhibiting RNF8‐mediated ubiquitination, OTUB2 limits NHEJ and promotes HR through the activation of YAP/TAZ, maintaining genomic stability during DSB repair.^263^ USP22 also plays a role in DSB repair by deubiquitinating H2B‐K120, necessary for chromatin remodeling. The absence of USP22 impairs H2AX phosphorylation and reduces NHEJ efficiency, underscoring its importance in genomic stability.^210^

In mismatch repair (MMR) and nucleotide excision repair (NER), OTUB1 stabilizes CHK1 through deubiquitination, enhancing repair and radiation resistance.^253^ Additionally, it inhibits the ubiquitination of MMR protein MSH2, regulating DNA damage signaling and resistance to genotoxic agents.^351^ OTUB1 also stabilizes and activates p53, further supporting DDR by promoting DNA repair pathways.^351^

USP22 and A20 are crucial for chromatin remodeling and DDR protein recruitment. USP22 deubiquitinates H2B‐K120, facilitating the recruitment of repair proteins.^210^ In contrast, A20 binds and inhibits RNF168, disrupting its interaction with H2A and preventing the accumulation of repair proteins like 53BP1, impacting DDR and enhancing cancer cell resistance to treatments.^352^

In conclusion, DUBs are integral to the regulation of diverse DNA damage repair pathways. USP1, OTUB1, OTUB2, USP22, and A20 exhibit varied mechanisms through which they influence DNA repair processes. Understanding these context‐specific roles emphasizes the therapeutic potential of targeting DUBs in cancer treatment and underscores the need for ongoing research to reveal their precise regulatory functions and interactions within DDR pathways.

### 4.4 DUBs and tumor cell metabolism

The metabolic characteristics of tumor cells often differ significantly from those of normal cells, with the most notable phenomenon being the Warburg effect. This effect describes how tumor cells preferentially generate energy through glycolysis rather than the more efficient oxidative phosphorylation, even under sufficient oxygen conditions.^353^ Additionally, tumor cells regulate lipid and amino acid metabolism to meet their needs for energy, biosynthetic precursors, and maintenance of redox balance. By exploring the diverse roles of DUB family proteins in tumor cell energy metabolism, we can better understand their complex roles in tumor development and provide a scientific basis for developing new therapeutic strategies (Figure 4B).

DUBs significantly impact the glycolytic pathway, a primary metabolic route in tumor cells, especially under the Warburg effect where cells favor glycolysis over oxidative phosphorylation even in the presence of oxygen. USP37, for instance, has been shown to regulate glycolysis by targeting c‐Myc. In H1299 cells, USP37 knockdown leads to decreased glucose consumption and lactate production, which can be reversed by re‐expressing c‐Myc. This indicates that USP37 enhances the Warburg effect through c‐Myc stabilization.^237^ Similarly, OTUB1 promotes aerobic glycolysis by preventing MYC degradation, thereby facilitating breast cancer cell proliferation via increased expression of hexokinase 2 (HK2).^354^ USP14 also supports glycolytic metabolism in OSCC by deubiquitinating and stabilizing phosphofructokinase‐1 liver type (PFKL), which boosts tumor cell proliferation and migration.^184^ In contrast, A20 reduces PFKL stability, thereby inhibiting glycolysis and liver cancer cell growth.^308^

In terms of lipid metabolism, USP22 plays a crucial role by directly interacting with and deubiquitinating peroxisome proliferator‐activated receptor gamma (PPARγ). This deubiquitination stabilizes PPARγ, which is vital for de novo fatty acid synthesis. High levels of USP22 expression in HCC correlate with the expression of PPARγ, ATP‐citrate lyase (ACLY), and acetyl‐CoA carboxylase, and are associated with poor prognosis, underscoring its role in lipid metabolism and tumor progression.^214^

Hypoxia, a common feature of the TME, induces specific metabolic adaptations mediated by hypoxia‐inducible factor 1‐alpha (HIF‐1α). DUBs such as USP25 and USP11 regulate HIF‐1α stability, thereby influencing hypoxia‐adapted metabolic pathways. USP25 enhances HIF‐1α transcriptional activity by deubiquitinating it under hypoxic conditions, promoting glycolysis and enabling the survival of pancreatic ductal adenocarcinoma (PDAC) cells in hypoxic regions.^216^ Similarly, USP11 stabilizes HIF‐1α, driving glycolytic pathways involving PDK1 and lactate dehydrogenase A, contributing to HCC proliferation and metastasis.^169^

DUBs play multifaceted roles in the metabolic reprogramming of tumor cells by stabilizing critical metabolic regulators. This supports tumor cell proliferation, migration, and survival, making DUBs potential targets for cancer therapy. Understanding these regulatory mechanisms offers insights into novel therapeutic strategies aimed at disrupting tumor metabolism to hinder cancer growth and progression.

### 4.5 DUBs and tumor immunity

Tumor cells develop a multitude of complex mechanisms to escape immune surveillance and suppress antitumor immune responses, thereby fostering an environment favorable for their growth and survival. Programmed death‐ligand 1 (PD‐L1) is a critical immune checkpoint protein that, when overexpressed on tumor cells, binds to PD‐1 receptors on T cells, resulting in immune evasion.^355^ Achieving a detailed understanding of how DUBs regulate PD‐L1 is essential for the development of effective cancer immunotherapies (Figure 4B).

PD‐L1 stability is intricately regulated by several DUBs, which directly influence its expression levels on tumor cells. For instance, USP5 stabilizes PD‐L1 by removing its ubiquitin tags, thereby preventing its degradation. Elevated levels of USP5 in NSCLC are associated with increased PD‐L1 expression and poor patient outcomes. This highlights USP5's role in promoting immune evasion by stabilizing PD‐L1, which inhibits T cell activity.^147^

Similarly, USP22 is another DUB that stabilizes PD‐L1 by direct deubiquitination. USP22 operates through the USP22–CSN5–PD‐L1 axis, enhancing PD‐L1 levels and hindering T cell cytotoxicity, which in turn promotes tumorigenesis.^213^ Additionally, USP7 has been shown to enhance PD‐L1 stability directly. The inhibition of USP7 disrupts the PD‐L1/PD‐1 interaction, increasing the susceptibility of cancer cells to T cell‐mediated killing both in vitro and in vivo.^153^

USP2 also facilitates immune evasion by stabilizing PD‐L1. Depletion of USP2 induces PD‐L1 degradation, thereby enhancing antitumor immunity. Combining USP2 depletion with anti‐PD‐1 therapy exhibits a synergistic antitumor effect, underscoring USP2's role in immune escape and its potential as a therapeutic target.^137^

In lung cancer, the deletion of A20 activates the TBK1–STAT1–PD‐L1 axis, promoting tumor cell evasion from CD8+ T cell‐mediated surveillance.^356^ In melanoma, increased A20 expression enhances STAT3 activity and PD‐L1 expression, thereby impairing CD8+ T cell functions.^357^

Beyond PD‐L1, OTUD1 displays dual roles in tumor immunity. It enhances antitumor immunity by regulating iron transport through the OTUD1–IREB2–TFRC axis, yet it also contributes to CRC immune escape via the TAM/TNFα–OTUD1–FGL1 pathway within the liver microenvironment.^271^, ^279^ Additionally, OTUD5 deubiquitinates and stabilizes STING, playing a role in radiation‐mediated antitumor immunity.^298^

In conclusion, DUBs significantly modulate tumor immunity by regulating the stability of key immune regulatory proteins like PD‐L1. These interactions can either promote immune evasion or enhance immune surveillance, indicating the potential of targeting DUBs in cancer immunotherapy. Understanding the precise mechanisms through which DUBs influence PD‐L1 and other immune regulators will be crucial for advancing effective cancer treatments.

### 4.6 DUBs and TME

The TME plays a critical role in promoting tumor growth and immune evasion. This complex milieu consists of various cell types, including immune cells, fibroblasts, and endothelial cells, along with cytokines and other chemical signals.^353^ DUBs within the TME modulate the immune responses by regulating the stability and function of numerous proteins, directly impacting tumorigenesis and immune evasion mechanisms (Figure 4B).

USP7 is crucial for macrophage homeostasis, particularly in balancing M1 and M2 phenotypes. Its inhibition reprograms M2 macrophages, leading to increased CD8+ T cell proliferation and improved antitumor responses. In Lewis lung carcinoma models, USP7 inhibitor treatment reduced tumor growth and enhanced M1 macrophage and CD8+ T cell infiltration via the p38 MAPK pathway.^154^

In PDAC, USP22 deletion reshapes the TME by reducing myeloid cell infiltration while promoting T and NK cell infiltration, thereby enhancing the response to combination immunotherapy and suppressing metastasis in a T cell‐dependent manner.^358^ Similarly, USP14 stabilizes IDO1, promoting immune suppression in CRC. USP14 inhibition decreases IDO1 levels, increases CD8+ T cell infiltration, reverses immune tolerance, and sensitizes CRC cells to anti‐PD‐1 therapy.^359^

OTUD4 contributes to tumor immune escape by stabilizing CD73, thus inhibiting CD8+ T cell function and IFN‐γ production.^290^ Conversely, increased USP18 expression in tumor cells inhibits carcinogenesis, while decreased USP18 expression fosters tumor growth by reducing IFN‐γ synthesis and CTL survival in the TME.^360^

OTUB1 enhances antitumor immunity by modulating IL‐15 ubiquitination, which activates CD8+ T cells and matures NK cells.^361^ Additionally, OTUD6B expression in tumors correlates with macrophage infiltration and T cell activity, shaping the tumor immune microenvironment.^362^

TME is a highly complex and dynamic system involving various cell types and signaling pathways. DUBs play significant roles in key immune processes and cell types, thereby modulating immune responses and the infiltrative capacity of immune cells. Targeting DUBs presents a promising therapeutic approach, and continued research in this area will be instrumental in developing novel strategies to enhance antitumor immune responses and improve the efficacy of existing treatments.

## 5 DUBs AND OTHER DISEASES

**FIGURE 5:**
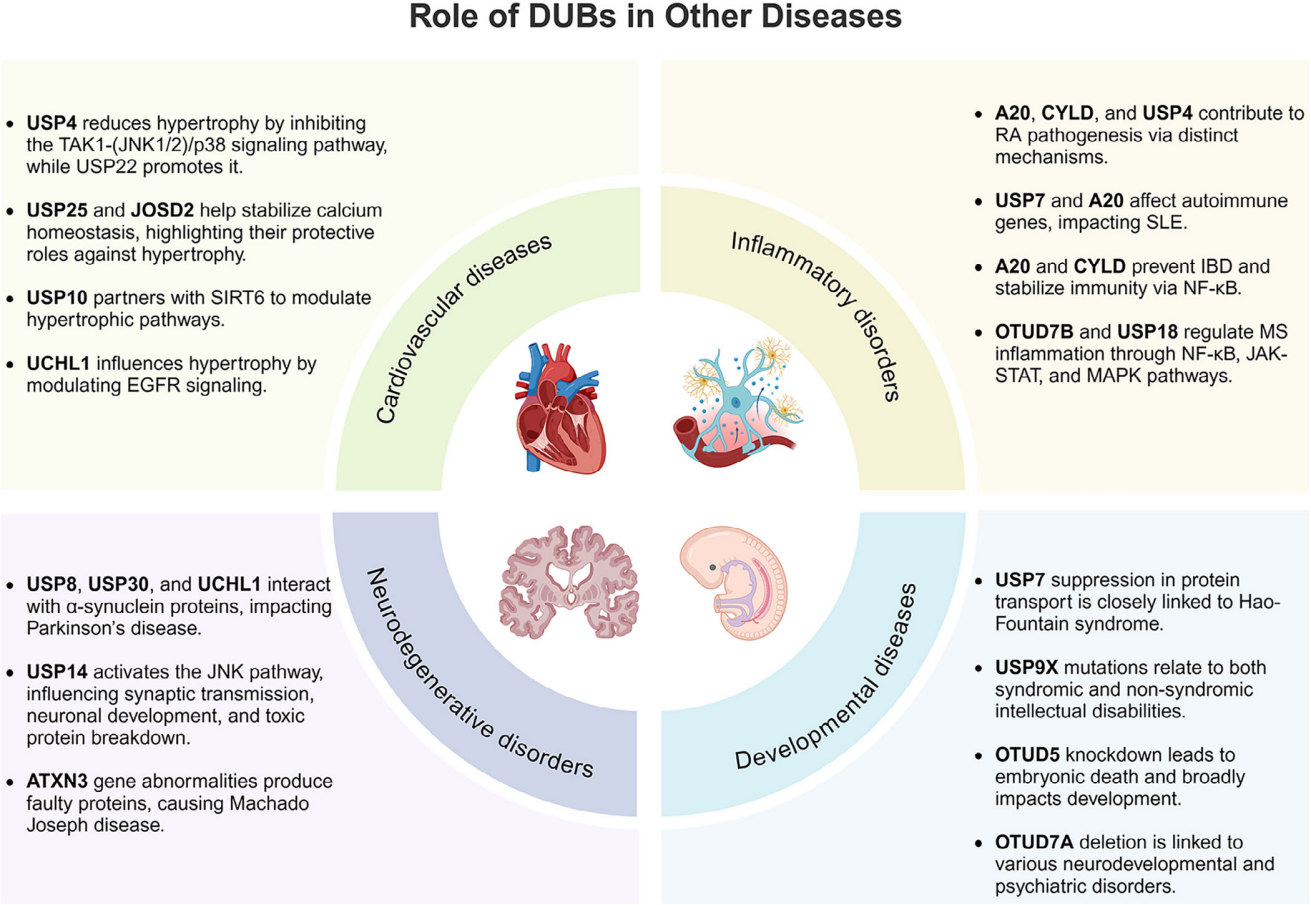
Role of DUBs in other diseases. This figure underscores the therapeutic potential of DUBs by illustrating their crucial roles in neurodegenerative, cardiovascular, inflammatory, and developmental diseases.

DUBs exhibit crucial, multifaceted roles in diseases beyond cancer, underscoring their potential as therapeutic targets. In neurodegenerative disorders such as Parkinson's and Huntington's diseases (PD and HD), DUBs are vital for modulating neuronal health and controlling neurotoxic aggregates, making them promising targets for therapeutic intervention. In cardiovascular diseases, DUBs regulate key processes like autophagy, metabolic pathways, and oxidative stress, influencing the development of cardiac hypertrophy and myocardial infarction (MI). They also play a significant role in inflammatory disorders by adjusting immune signaling pathways and maintaining the balance between pro‐ and anti‐inflammatory signals, impacting diseases like rheumatoid arthritis (RA) and multiple sclerosis (MS). Furthermore, DUBs are vital in developmental diseases where functional mutations can lead to severe neurodevelopmental problems, highlighting their importance in proper development. Gaining a deeper understanding of the diverse roles that DUBs play in diseases helps elucidate the mechanisms of disease development and paves the way for novel therapeutic approaches across a spectrum of conditions (Figure 5).

### 5.1 Role of DUBs in neurodegenerative disorders

DUBs play crucial roles in neurodegenerative disorders maintaining neuronal health and are increasingly recognized for their involvement in neurodegenerative disorders.^363^, ^364^ Neurodegenerative diseases are characterized by the progressive loss of specific neuron populations due the formation of neurotoxic aggregates in the brain, including PD, Alzheimer's disease (AD), and polyglutamine diseases.^365^ Recent evidence highlights the significant roles of DUBs in neurodegenerative disorders, making them potential therapeutic targets.

A polymorphic USP8 allele (USP8D442G) have been significantly found in Chinese PD patients, which could enhance interaction with α‐synuclein, leading to its accumulation and altered localization, highlighting this polymorphism as a potential therapeutic and diagnostic target for PD.^366^ HD, driven by polyglutamine‐expanded mutant huntingtin (mHTT), is ameliorated by the deubiquitinase Usp12, which protects against mHTT toxicity and enhances autophagy in neurons. Notably, Usp12's neuroprotective and autophagy‐inducing effects do not depend on its catalytic activity, highlighting its unique role in regulating neuronal proteostasis.^367^ USP14 is a DUB enzyme crucial for maintaining the nervous system's health and function by regulating the cellular pool of monomeric ubiquitin. In USP14‐deficient mice, developmental deficits are observed in neuromuscular junctions and motor neuron endplates, accompanied by activation of pathways involving mixed lineage kinase 3 and downstream targets like c‐jun N‐terminal kinase (JNK). USP14 plays a vital role in synaptic transmission and neuronal development, especially at the neuromuscular junctions.^368^ USP14 influences the clearance of ubiquitinated toxic proteins by inhibiting their proteasome‐mediated degradation, exemplified by the enhanced degradation of tau and ATXN3 upon inhibition of its DUB activity.^369^ USP14 inhibitor have been developed as a potential therapeutic agent to protect cells from neurodegenerative stressors cell by enhancing proteasome activity and promoting the degradation of proteotoxic proteins.^370^, ^371^ Furthermore, a broader role of USP14 in memory formation and neuroprotection against ischemic injury, highlighting it as a promising therapeutic target in neurodegenerative disorders.^372^, ^373^ A recent study reports that loss of USP30 enhances mitophagy and reduces neurodegeneration by counteracting α‐synuclein's effects, and a USP30 inhibitor MTX115325 shows potential as a disease‐modifying therapy for PD.^374^

ATXN3 plays significant roles in neurodegenerative disorders, particularly in spinocerebellar ataxia type 3 (SCA3), also known as Machado‐Joseph disease. This disease is triggered by CAG repeat expansions in the *ATXN3* gene, leading to a malformed ATXN3 protein with an extended polyglutamine tract,^375^ which results in toxic protein aggregates. Emerging therapies, including RNA‐based silencers and CRISPR/Cas9, target mutant ATXN3 expression, showing promise in reducing oxidative stress and mitochondrial dysfunction in SCA3 models.^376^, ^377^, ^378^ These results suggest that targeting mutated ATXN3 could offer a promising therapeutic strategy for SCA3, highlighting the need to develop effective ATXN3 inhibitors.

UCHL1, a key DUB enzyme abundant in the brain, plays a vital role in maintaining neuronal health by regulating ubiquitin homeostasis at synapses.^379^ Mutations and modifications in UCHL1 have been associated with human neurodegenerative disorders such as PD and AD, as well as brain injuries.^380^ UCHL1 acts as both a deubiquitinase and a ligase—its ligase activity, which extends Lys63–polyubiquitin chains on α‐synuclein, is enhanced by oligomerization but can be disrupted by disease‐associated mutations.^381^ Structural studies reveal that UCHL1 variants like I93 M increase PD's risk by disrupting activity, while S18Y is protective by reducing dimerization and ligase activity. Beyond α‐synuclein regulation, UCHL1 interacts with and stabilizes TrkB, a neurotrophin receptor critical for neuronal survival and synaptic plasticity, highlighting a mechanism where UCHL1 modulates BDNF–TrkB signaling by preventing TrkB degradation.^382^ These findings underscore the multifaceted role of UCHL1 in neuroprotection and its potential as a therapeutic target in neurodegenerative disorders.

In conclusion, DUBs are fundamental to preserving neuronal function through their regulation of protein homeostasis and mitigation of aggregate‐prone proteins via proteasome and autophagy pathways. Their contribution to neurodegenerative conditions underscores their potential as therapeutic targets, warranting further exploration in neuronal models to advance treatment strategies for these debilitating disorders.

### 5.2 Role of DUBs in cardiovascular diseases

DUBs are crucial in cardiovascular diseases as they prevent degradation of substrate proteins by the ubiquitin–proteasome system, thereby regulating autophagy, metabolism, and oxidative stress.^383^ They play significant roles in cardiac hypertrophy, MI, and diabetes‐related cardiac conditions, influencing disease onset and progression.

DUBs play pivotal roles in the development and regulation of cardiac hypertrophy through their involvement in various cellular signaling pathways. These enzymes influence key processes such as inflammation, apoptosis, autophagy, and calcium homeostasis, all of which contribute to the cardiac hypertrophic response. DUBs such as USP19, USP2, and USP4 have been identified as potential biomarkers and regulators in cardiac hypertrophy.^384^, ^385^, ^386^ USP4, for instance, exhibits an antimyocardial hypertrophy effect by inhibiting the TAK1–(JNK1/2)/p38 signaling pathway, while USP22 exacerbates hypertrophy by activating the same pathway.^387^ Moreover, USP14 fosters cardiac hypertrophy by enhancing GSK‐3β activation, suggesting its role as a modulator of hypertrophic signaling.^388^ The stabilization of calcium homeostasis by DUBs like USP25 and JOSD2 further underscores their protective roles against hypertrophy, as they regulate SERCA2a levels.^389^ USP10 regulates hypertrophic pathways via enhancing the stability and function of SIRT6, leading to downregulation of the Akt signaling pathway and attenuation of maladaptive cardiac hypertrophy.^390^ These insights highlight the potential of targeting DUBs for therapeutic interventions to treat cardiac hypertrophy and related cardiovascular diseases.

DUBs can be involved in the pathogenesis and progression of MI by modulating various cellular processes such as apoptosis, ferroptosis, and pyroptosis. USP47 is upregulated in MI, enhancing NF‐κB activity and promoting cardiomyocyte apoptosis during ischemia/reperfusion injury, thus its knockdown may serve as a therapeutic target by repressing MI progression.^391^ Conversely, USP49 activates DUSP1–JNK1/2 signaling to reduce apoptosis of cardiomyocytes, offering cardio‐protective effects against ischemia–reperfusion injury.^392^ Ferroptosis, another form of programmed cell death, is regulated by USP7 and OTUD5 during MI, with USP7 knockdown reducing ferroptosis via the p53 pathway, while OTUD5 prevents ferroptosis by stabilizing GPX4, thus both alleviating myocardial injury.^13^, ^393^ Moreover, USP11 and USP47 has been shown to worsen ischemia–reperfusion injury by enhancing pyroptosis through aggravating immune inflammation.^394^, ^395^ These findings underscore DUBs as potential therapeutic targets in MI management.

In diabetic heart disease, DUBs play a significant role in regulating mitochondrial function and cardiac health. Specifically, in cardiac dysfunction amelioration in type 2 diabetes mellitus (T2DM), the S‐sulfhydration of USP8 enhances its deubiquitination of parkin, which facilitates parkin's translocation into mitochondria and mitigates oxidative stress and mitochondrial apoptosis.^396^ Additionally, the USP10/Notch1 axis mediates the cardiac protective effects of follicle‐like protein 1 in T2DM with MI,^397^ highlighting USP10 as a potential therapeutic target for managing diabetic heart disease.

Taken together, many DUBs are crucial regulators of cardiovascular diseases, influencing key cellular processes such as inflammation, apoptosis, autophagy, and calcium homeostasis. These findings highlight the therapeutic potential of targeting DUBs to treat cardiovascular diseases, warranting further research into their specific roles and mechanisms.

### 5.3 Role of DUBs in inflammatory disorders

DUBs play crucial roles in inflammatory disorders by modulating ubiquitin signaling pathways, significantly impacting central and peripheral immune responses often disrupted in conditions such as RA, systemic lupus erythematosus (SLE), IBD, psoriasis, and MS.^398^ These enzymes regulate the balance between proinflammatory and anti‐inflammatory signals, and their dysregulation contributes to the progression of various inflammatory disorders, highlighting them as potential therapeutic targets for treatment.

RA is classified as a systemic poly‐articular chronic autoimmune joint disease that primarily affects the hands and feet, with pathological features including immune cell infiltration, hyperplasia of the synovial lining, pannus formation, and destruction of articular cartilage and bone.^399^ A20 plays a crucial role in negatively regulating NF‐κB signaling in response to inflammatory stimuli and has been implicated as a susceptibility gene for RA. Myeloid‐specific A20‐deficient mice spontaneously developed severe destructive polyarthritis, characterized by increased inflammatory cytokines, sustained NF‐κB activation, and enhanced osteoclastogenesis, indicating A20's critical, cell‐specific role in the disease.^400^ CYLD, another DUB, also impacts NF‐κB and is involved in the pathogenesis of synovial inflammation in RA.^401^ Moreover, USP4 interacts with RORγT in Th17 cells, enhancing IL‐17A production, making it a promising target for developing new treatments for T‐helper 17 (Th17)‐mediated autoimmune diseases like RA.^402^ Together, these DUBs highlight novel therapeutic targets and pathways in RA management.

SLE is a complex chronic autoimmune disease characterized by heightened B and T cell responses and a loss of tolerance to self‐antigens, leading to widespread organ damage.^403^ The regulation of autoimmune‐related genes through ubiquitination and deubiquitination processes is critical in SLE pathogenesis. In SLE patients, the DUB USP7 is a crucial regulator of the protein levels of the human IFNα‐2 receptor (IFNAR1). It stabilizes IFNAR1 by dismantling IFNAR1‐dependent polyubiquitin chains, thereby activating the IFNα pathway. USP7 is significantly overexpressed in SLE patients and shows a positive correlation with IFN scores, SLE disease activity index scores, and anti‐double‐stranded DNA, suggesting that USP7 may be associated with SLE disease activity through the stabilization of IFNAR1.^404^ Furthermore, it is reported that genetic variations in the A20 OTU domain are associated with an increased risk of SLE.^405^ Although A20 typically regulates NF‐κB, mutations in its OTU domain do not lead to enhanced NF‐κB signaling.^406^ Instead, they cause an upregulation of the PADI4 enzyme, which promotes protein citrullination and neutrophil extracellular trap (NET) formation, enhancing autoimmune responses. Individuals with A20 DUB polymorphisms in SLE patients exhibit increased NETs and autoantibodies to citrullinated epitopes, indicating that these genetic alterations increase susceptibility to SLE through these mechanisms.^407^ These insights suggest that modulating DUB can offer novel therapeutic strategies for managing SLE, aiming to restore immune balance and mitigate disease progression.

DUBs are integral to the pathogenesis and progression of IBD by modulating ubiquitin signaling pathways that regulate inflammatory processes. Prominent among these is A20, which tempers NF‐κB signaling—a key pathway in IBD development—by removing K63‐linked polyubiquitin chains from NF‐κB signaling factors like NEMO, RIPK1, and TRAF6, thus maintaining immune homeostasis.^32^ Mice lacking A20 exhibit severe intestinal inflammation, underlining its role in intestinal health.^408^ Similarly, CYLD, another DUB, suppresses NF‐κB activation by deubiquitinating TRAF2 and TRAF6, further preventing excessive inflammation.^76^

Some DUBs regulate the pathogenesis of psoriasis, a chronic immune‐mediated disorder characterized by skin, nail, and joint inflammation, which involves dysregulated cytokine signaling, activating transcription factors like STAT3, STAT1, and NF‐κB.^409^ For instance, USP4 and USP17 enhance Th17‐mediated gene expression and IL‐17 secretion by deubiquitinating RORγT, contributing to inflammatory pathways.^402^ USP4 also regulates TNFα‐induced NF‐κB activation by deubiquitinating TAK1,^410^ highlighting its potential as a therapeutic target. These findings underscore the critical involvement of DUBs in managing inflammatory pathways in psoriasis.

MS is a chronic autoimmune disease that causes inflammation, leading to demyelination and immune cell infiltration in the central nervous system, affecting the protective covering around nerves in the brain and spinal cord.^411^ DUBs regulate key signaling pathways such as NF‐κB, JAK–STAT, and MAPK to influence T cell differentiation, activation, and survival, thus affecting inflammation in MS.^412^ For instance, OTUD7B enhances T cell receptor (TCR) signaling and exacerbates experimental autoimmune encephalomyelitis (EAE) in a MS mouse model by deubiquitinating Zap70 and preventing Sts1/Sts2 binding, whereas its knockout diminishes T cell activation and Th1 cell differentiation by reducing TCR–CD28 signaling.^413^ USP18 interacts with TAK1 to reduce its ubiquitination and, by decreasing NF‐κB and NFAT activation, impairs IL‐2 production, thus inhibiting Th17 polarization and EAE pathogenesis, with USP18 deficiency leading to reduced Th17 differentiation.^414^ These insights underscore DUBs’ significant roles in MS pathophysiology, offering targets for therapeutic intervention.

In summary, DUBs are vital regulators of the inflammatory process, influencing immune signaling, cytokine production, and the pathogenesis of inflammatory disorders. Continued exploration of their roles could lead to breakthroughs in the treatment of inflammatory disorders, providing more targeted and effective therapies.

### 5.4 Role of DUBs in developmental diseases

Notably, emerging evidence points to a critical involvement of DUBs in developmental diseases, often resulting from mutations that disrupt their normal function. These mutations typically lead to severe developmental disorders, marked by early‐onset neurological deficits, highlighting the significance of DUBs in neurodevelopmental processes. Such disorders are frequently attributed to loss‐of‐function mutations, underscoring a crucial dependency on DUB activity for normal development.

Hao‐Fountain syndrome, a developmental disorder characterized by seizures, behavioral abnormalities, hypogonadism, and hypotonia, is caused by heterozygous mutations in the *USP7* gene.^415^ The pathogenesis of the syndrome is associated with USP7's roles in cellular protein trafficking, specifically through the regulation of the MAGE–L2–TRIM27 complex, which plays a critical part in retromer‐dependent endosomal recycling.^416^ This insight aligns with similar phenotypic features observed in related syndromes, indicating that aberrant endosomal sorting underlies the disorder. Mutations in the X‐linked deubiquitinase USP9X are associated with both syndromic and nonsyndromic intellectual disabilities.^417^, ^418^ Specifically, USP9X regulates TGF‐β signaling via SMAD4 deubiquitination, stabilizes the centriole replication factor STIL, and regulates cilia assembly, all of which have overlapping phenotypes with diseases like microcephaly and ciliopathies.^419^ Additionally, USP9X influences dendritic spine development through the stabilization of ankyrin‐G, with variants in ankyrin‐G linked to neurodevelopmental disorders. Notably, TGF‐β supports cortical spine development via USP9X‐dependent stabilization of ankyrin‐G, suggesting interconnected pathways in USP9X‐related neurodevelopmental coordination, warranting further investigation into these interactions and their gender‐specific manifestations.^420^

OTUD7A, a K11‐specific OTU deubiquitinase located in the 15q13.3 locus, is implicated in a range of neurodevelopmental and psychiatric disorders when deleted.^421^, ^422^ Studies indicate OTUD7A's crucial role in neurodevelopment, evidenced by its control over dendritic branching and its knockout leading to neurodevelopmental deficits and abnormal EEGs in mice.^423^, ^424^ Moreover, biallelic OTUD7A missense variants in individuals with epilepsy support its association with the condition.^425^ Despite these findings, the detailed molecular mechanisms, including its interaction with E3 ligases and substrates, remain to be elucidated, which could provide insights into distinct epilepsy forms. Moreover, bi‐allelic loss‐of‐function mutations in OTUD6B result in global developmental delay, feeding difficulties, structural brain abnormalities, and congenital heart disease.^426^ While OTUD6B is linked to protein translation and proteasome stability, its precise mechanistic role remains unclear, necessitating further research to understand its impact on differentiation processes.^427^ Hemizygous missense and deletion variants in OTUD5, an X‐linked OTU deubiquitinase that cleaves K48‐ and K63‐linked ubiquitin chains, cause a male‐specific congenital disorder characterized by central nervous system, craniofacial, cardiac, skeletal, and genitourinary anomalies.^428^ In mice, Otud5 knockout results in embryonic lethality, and OTUD5‐depleted human ESCs show neuroectodermal differentiation defects that can be rescued by wild‐type OTUD5.^428^ A patient variant affecting only K48‐linked ubiquitin cleavage fails to rescue differentiation, indicating the disorder stems from loss of OTUD5's activity on degradative K48‐chains. OTUD5 prevents the degradation of chromatin remodelers like ARID1A/B, HDAC2, and HCF1, essential for enhancer activation during differentiation.^427^ These findings highlight the critical role of K48‐ubiquitin chain cleavage in coordinating chromatin remodeling in early development, with further research needed to explore its broader embryogenic regulation.

Overall, DUBs are vital in modulating ubiquitin signaling necessary for embryonic and postnatal development, with their dysregulation associated with congenital disorders. Despite advancements, questions about their substrate specificity, regulatory mechanisms, and full physiological roles remain, often due to their study relying on incomplete protein variants. Advances in genomic sequencing now enable the identification of key DUBs as potential disease‐causing genes, suggesting that prioritizing these for research could reveal new developmental pathways and improve disease diagnosis and treatment, influencing advances in precision medicine.

## 6 TARGETING DUBs WITH SMALL‐MOLECULE INHIBITORS

In the field of cancer treatment research, DUBs have garnered significant attention due to their crucial role in regulating protein stability and cellular signaling. These enzymes influence the fate of various proteins, including tumor suppressor proteins and oncogenic proteins, by removing ubiquitin tags. Developing targeted cancer treatment strategies for DUBs not only allows for more precise intervention in critical pathways of tumor growth but also offers potentially more effective treatment options for cancer patients, thereby improving treatment efficacy and quality of life. However, developing effective inhibitors for DUBs present multiple challenges, including their high homology, complex three‐dimensional structures, dynamic conformational changes, and regulation by posttranslational modifications and protein interactions. Despite these challenges, researchers have successfully identified potential inhibitors targeting specific DUBs through computer‐aided design and virtual screening techniques (Table 2).

**TABLE 2:** DUB small‐molecule inhibitors.

Name	Structure	Target	Enzymatic activity and affinity parameters	Biological activity	Clinically related research	Mechanisms of action	Cancer type	References
1 m	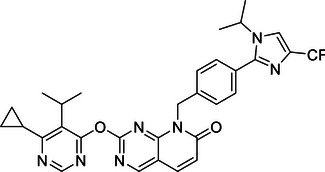	USP1	Enzymatic IC_50 _= 8.8 ± 1.4 nM	Cell type: NCI‐H1299 and MDA‐MB‐436 cells Effective dose: 100 nM	/	Regulate the cell cycle	Lung and BC	^429^
ML323	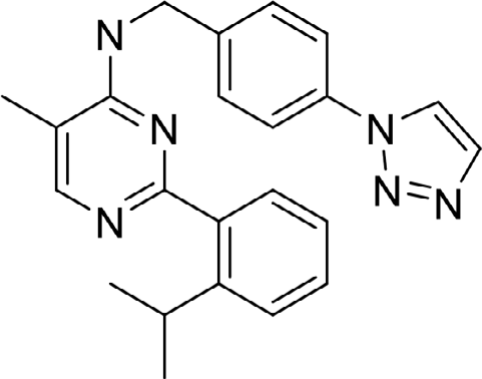	USP1	/	Cell type: Kyse30, Kyse450, Kyse510, and EC109 cells Effective dose: 20 µM	/	Induce apoptosis and DNA damage	Esophageal squamous cell carcinoma	^430^
Pimozide	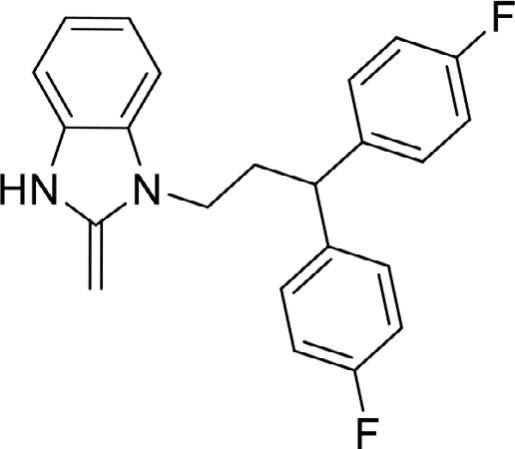	USP1	Ki = 0.5 µM	Cell type: H596 and NCI cells	Model: patients with amyotrophic lateral sclerosis Progress: phase II trial stage	Reversible, noncompetitive inhibition	Schizophrenia, Psychotic Disorders, Tourette Syndrome and Amyotrophic lateral sclerosis (ALS)	^431^
YCH2823	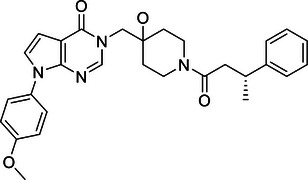	USP7	KD = 0.117 µM Enzymatic IC_50_ = 49.6 ± 2.9 nM	Cell type: LNCaP (IC_50_ = 0.15 nM), MV4‐11, (IC_50_ = 23.29 nM) MM.1S (IC_50_ = 14.24 nM) and CHP‐212 (IC_50_ = 1.69 nM)	/	Regulate the cell cycle	Prostatic cancer, acute myeloid leukemia, multiple myeloma and neuroblastoma	^432^
YCH3124	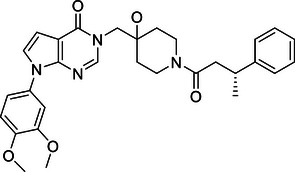	USP7	KD = 0.156 µM Enzymatic IC_50_ = 41.8 ± 3.6 nM	Cell type: LNCaP (IC_50_ = 3.6 nM) and CHP‐212 (IC_50_ = 9.9 nM)	/	Regulate the cell cycle	Prostatic cancer and neuroblastoma	^433^
Compound 7	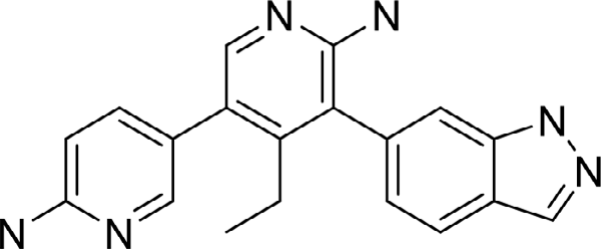	USP7	KD = 2.4 µM Enzymatic IC_50 _= 7.6 ± 0.1 µM	Cell type: HCT116 cells (IC_50_ = 81.4 ± 6.4 µM)	/	Regulate the cell cycle	Colon cancer	^434^
PROTAC 17	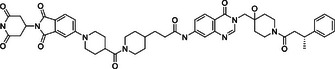	USP7	Degradation DC_50 _= 17 nM	Cell type: MM.1S cells Effective dose: 1 µM	/	Induce apoptosis	Multiple myeloma	^435^
Compound D1	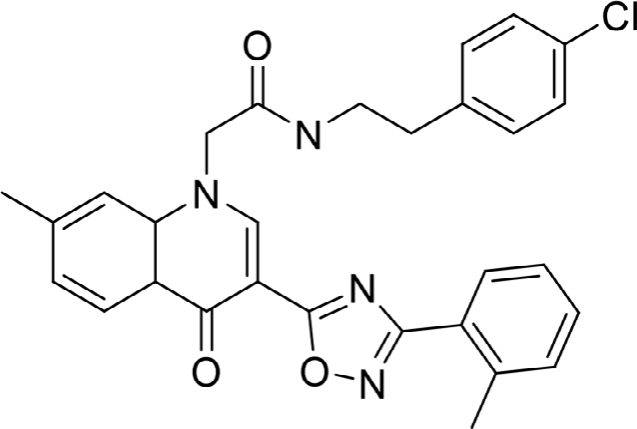	USP10	Enzymatic IC_50 _= 7.2 µM	Cell type: Bel‐7402 (IC_50_ = 2.1 µM) and HUH‐7 cells (IC_50_ = 3 µM)	/	Regulate the cell cycle and induce apoptosis	Hepatocellular carcinoma	^436^
Mitoxantrone	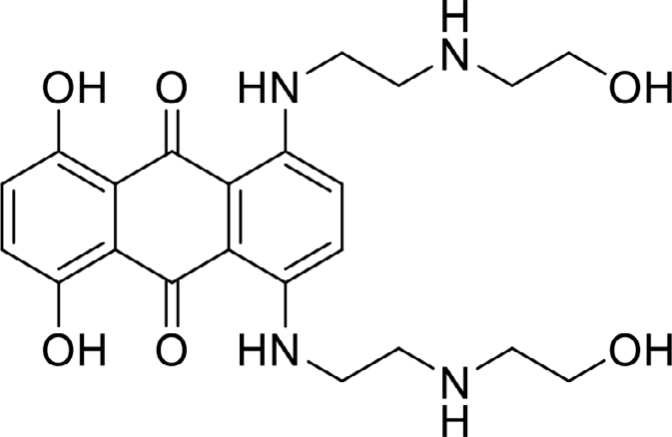	USP11	/	Cell type: Panc1 and PL5 cells (IC_50_ < 10 nM)	Model: participants with solid tumors Effective dose: 18 mg/m Progress: phase II trial stage	USP11 inhibition via unknown mechanism	Acute myeloid leukemia, neoplasms, BC, acute myelogenous leukemia, and lymphoblastic lymphoma	^437^
Spautin‐1	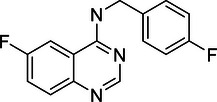	USP10 and USP14	/	Cell type: U87MG, LN229 and GL261 cells Effective dose: 5 µM	/	Disrupt the RAF–ERK pathway and glycolysis	Glioblastoma	^438^
BAY‐805	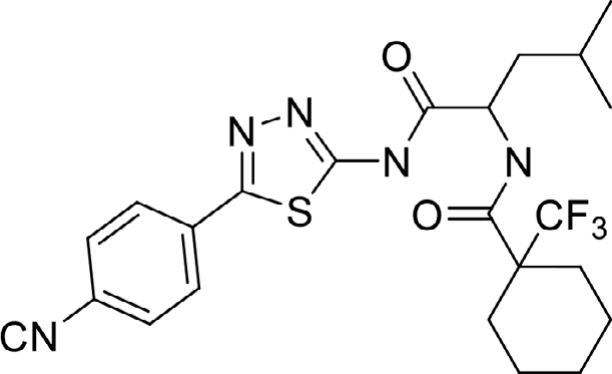	USP21	KD = 2.2 nM Enzymatic IC_50 _= 6 nM	/	/	Activate the NF‐κB pathway	/	^439^
Nifuroxazide	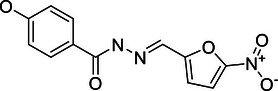	USP21	Degradation DC_50 _= 31.64 µM	Cell type: HepG2 cells Effective dose: 30 µM	/	Regulate cellular metabolism	Liver cancer	^440^
MOLHYB‐0436	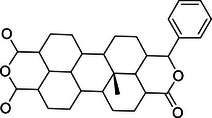	USP21	pKD = 6.023 Enzymatic pIC_50 _= 5.972	/	/	Disrupt the Wnt pathway.	Pancreatic cancer	^441^
Gentiopicroside	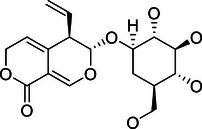	USP22	/	Cell type: MC38 and LLC1 cells Effective dose: 30 µM	Model: lung cancer xenograft mice model Effective dose: 4 mg	Enhances antitumor immunity	Colon cancer and lung cancer	^442^
CT1113	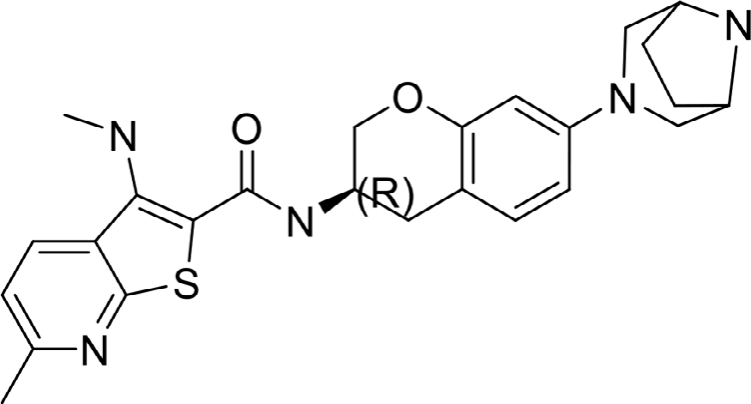	USP25 and USP28	USP25 KD = 11.1 µM USP28 KD = 12.2 µM USP25 Enzymatic IC_50 _= 26.1 nM USP28 Enzymatic IC_50 _= 3.9 nM	Cell type: HCY116, HGC27, and SMMC7721 cells Effective dose: 500 nM	Model: pancreatic and colon cancer CDX model Effective dose: 20 mg/kg	Reduce c‐MYC levels	Pancreatic and colon cancer	^443^
Compound 9p	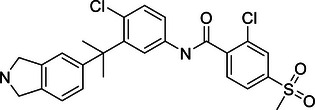	USP28	Enzymatic IC_50_ = 0.04 ± 0.02 µM	Cell type: HCT116 (IC_50_ = 38.11 ± 4.32 µM) and Ls174T (IC_50_ = 24.30 ± 2.73 µM) cells	/	Reduce c‐MYC levels	Colon cancer	^444^
P50429	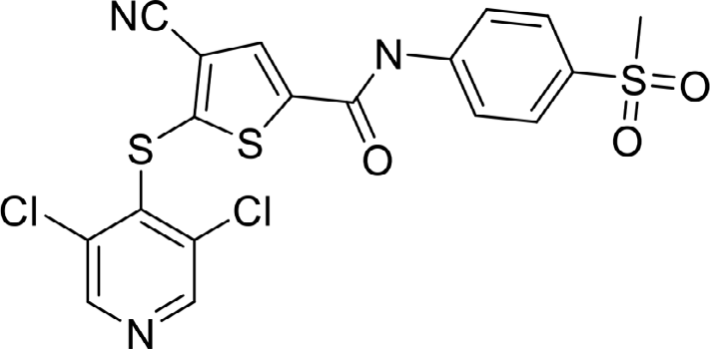	USP7 and USP47	USP7 Enzymatic IC_50 _= 5.3 ± 1 µM USP47 Enzymatic IC_50 _= 1.4 ± 0.2 µM	/	/	Unknown	/	^445^
Compound 17e	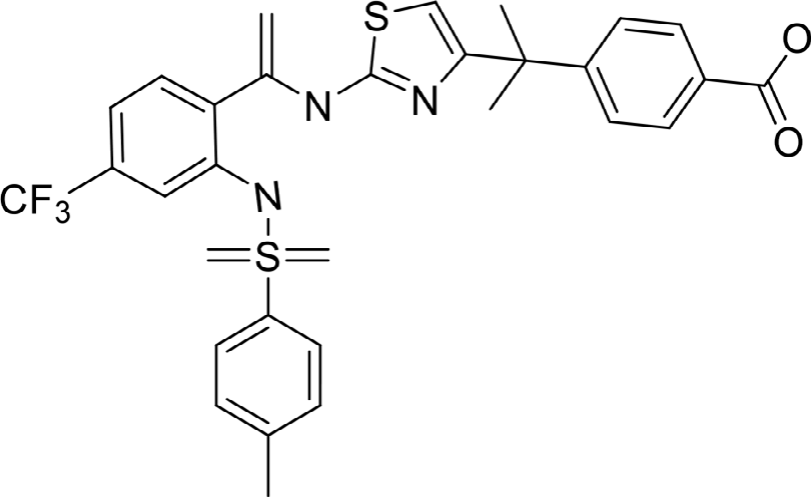	USP48	Enzymatic IC_50 _= 12.6 ± 1.24 µM	/	/	Unknown	/	^446^
Compound 61	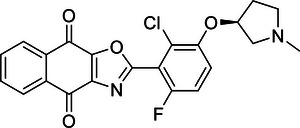	OTUB1	Enzymatic IC_50_ = 0.17 ± 0.02 nM	Cell type: H1975 (IC_50_ = 118 nM), EBC‐1 (IC_50_ = 172 nM), H1703 (IC_50_ = 145 nM), H23 (IC_50_ = 431 nM), A549 (IC_50_ = 1.0 µM) cells	Animal type: Mice Model: H1975 xenograft mouse model Effective dose: 5 mg/kg	Suppress the growth factor signaling	NSCLC	^447^
OTUB2‐IN‐1	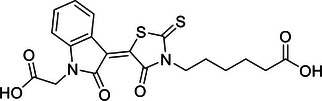	OTUB2	KD = 12 µM	Cell type: LL/2 tumor cells	Animal type: Mice Model: LL/2 tumor model, B16‐F10 tumor model, KLN205 tumor model	Regulate NF‐κB and Akt/mTOR pathways	Melanoma and squamous cell carcinoma	^448^
OTUDin3	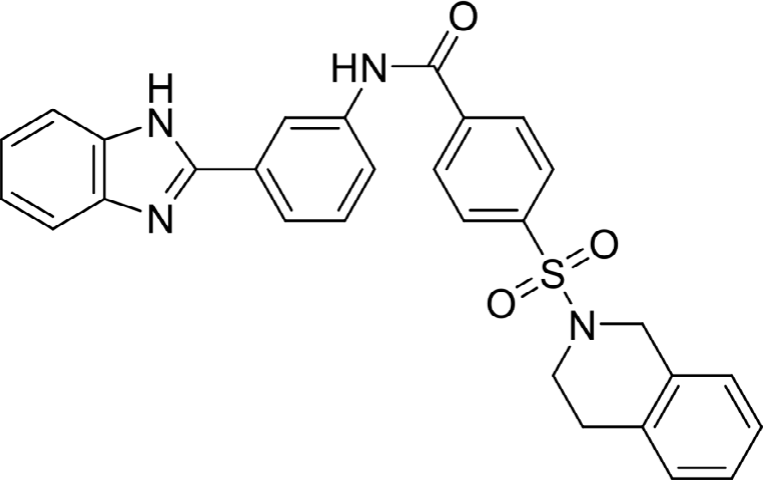	OTUD3	KD = 0.32 µM	Cell type: H1299 (IC_50_ = 8.1 µM), A549 (IC_50_ = 8.1 µM), H460 (IC_50_ = 8.1 µM), and H1650 (IC_50_ = 8.1 µM)	Animal type: Nude mice Model: H1299 xenograft model Effective dose: 10 mg/kg	Inhibit cell proliferation and induce apoptosis	NSCLC	^449^
Rolapitant	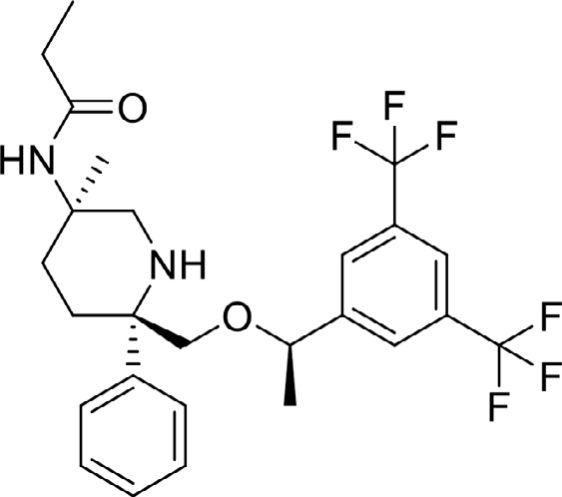	OTUD3	Affinity energy = −7.4 kcal/mol	Cell type: H1299 (IC_50_ = 13.9 µM), A549 (IC_50_ = 10.7 µM), H460 (IC_50_ = 11.3 µM), H1975 (IC_50_ = 12.05 µM), and BEAS‐2B (IC_50 _= 19.69 µM)	Animal type: Nude mice Model: A549 xenograft mode Effective dose: 50 mg/kg	Inhibit cell proliferation	Lung cancer	^450^
7Ai	/	OTUD7A	KD = 1.11 ± 0.62 µM	Cell type: A673 and SK‐N‐MC cells Effective dose: 10–20 µM	Animal type: Immunocompromised mice Model: Ewing sarcoma xenograft model Effective dose: 25 mg/kg	Substrate destabilization	Ewing's sarcoma and neuroblastoma	^451^
7Bi	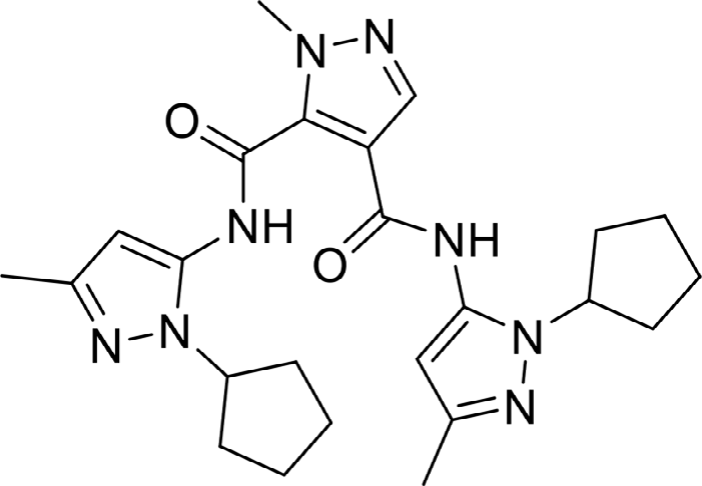	OTUD7B	Enzymatic IC_50_ = 40 µM	Cell type: A549 (IC_50_ = 2.5 µM)	/	Substrate destabilization	NSCLC and diffuse large B‐cell lymphoma	^452^
NSC112200	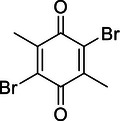	TRABID	Affinity energy = −20.28 kcal/mol Enzymatic IC_50 _= 3 µM	Cell type: LM2 and BT549 cells Effective dose: 10 µM	Animal type: C57BL/6 mice exhibited acute responses	Promote EZH2 protein degradation	CRC and TNBC	^453^, ^454^
CAS‐12290‐201	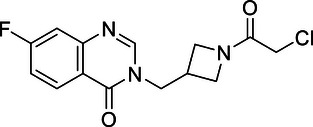	VCPIP1	Ki = 15.3 ± 4.6 µM Enzymatic IC_50 _= 70 nM	Cell type: / Animal type: /	/	Unknown	/	^455^
GK13S	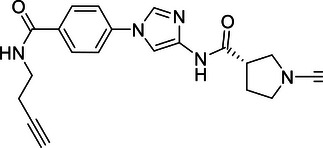	UCHL1	Kobs = 681 M^−1^ s^−1^	Cell type: HEK293 and U‐87 MG cells Effective dose: 50 nM	/	Unknown	Glioblastoma	^456^
VLX1570	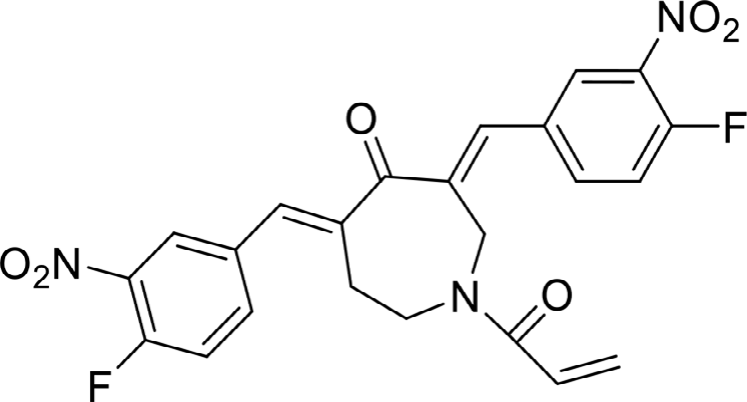	UCHL5 and USP14	UCHL1: KD = 14–18 µM USP14: KD = 1.5–18 µM	Cell type: HCT116 (IC_50_ = 50 nM) and KMS11 (IC_50_ = 43 ± 2 nM) cells	Model: WM‐xenografted mice. Effective dose: 4.4 mg/kg Model: Patients with relapsed and refractory MM Effective dose: 0.6 mg/kg Model: patients with relapsed and/or refractory multiple myeloma. Progress: phase II trial stage, prematurely ended.	Induction of apoptosis	Multiple myeloma	^457^, ^458^, ^459^, ^460^
iBAP	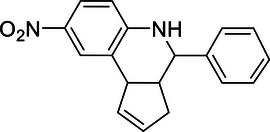	BAP1	Enzymatic IC_50_ = 253 nM	Cell type: HEK293T, THP1 and K562	Model: K562 (ASXL1–WT/Y591*) xenograft NSGS mice and patient‐derived tumor cells (ASXL1–WT/Q588*) NSGS mice Effective dose: 50 mg/kg	Downregulate leukemia‐associated genes	/	^461^
Capzimin	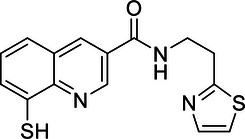	Rnp11	Enzymatic IC_50_ = 0.34 µM	Cell type: SR (GI_50_ = 0.67 µM), K562 (GI_50_ = 1 µM), NCI‐H460 (GI_50_ = 0.7 µM), and MCF7 (GI_50_ = 1 µM) cells	/	Induce apoptosis	BC and NSCLC	^462^
CSN5i‐3	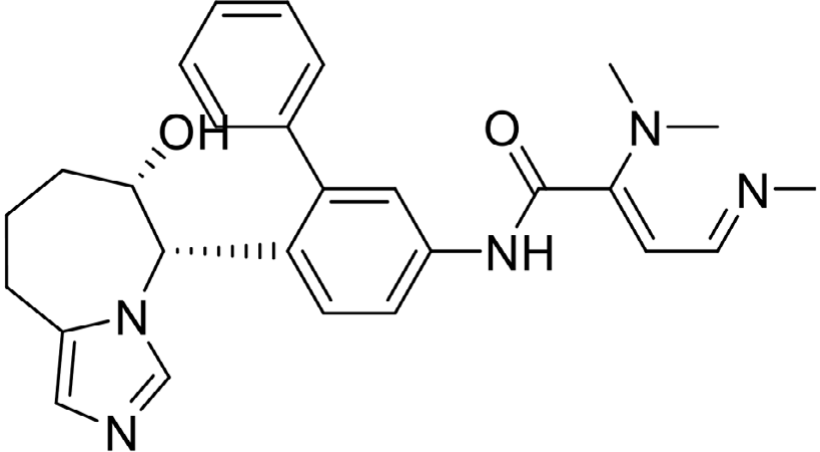	CSN5	Enzymatic IC_50_ = 0.0058 µM	Cell type: NCI‐H2030 and TE‐1 cells Effective dose: 1 µM	Model: Subcutaneous SU‐DHL‐1 xenografts in SCID‐bg mice Effective dose: 50 mg/kg	Regulate the cell cycle and induce apoptosis	Lymphoma	^463^

Abbreviations: BC, breast cancer; CDX, cell line‐derived xenograft; CRC, colorectal cancer; NSCLC, non‐small cell lung cancer; TNBC, triple negative breast cancer.

### 6.1 Targeting USPs

As research on the critical roles of the USP family in diseases increases, the discovery of small molecule drugs targeting the USP family has also been burgeoning. Existing studies have provided detailed reviews of the various small molecule inhibitors currently targeting the USP family.^24^, ^464^, ^465^ To provide the latest updates, this section will summarize some of the newly emerged USP inhibitors in the past 3 years, focusing on their development strategies and pharmacological activities (Table 2).

The development strategy for USP1 inhibitors involved designing and synthesizing a series of pyrido[2,3‐d]pyrimidin‐7(8H)‐one derivatives based on the structure of known inhibitors, with systematic exploration of their structure‐activity relationships. Compound 1m showed strong antiproliferative effects, arrested cancer cells in the S phase, acted as reversible and noncompetitive inhibitors, and enhanced the efficacy of the PARP inhibitor olaparib, demonstrating excellent oral pharmacokinetic properties in mice.^429^ ML323 is a USP1 inhibitor that shows specifically antagonizing the USP1‐UAF1 complex without affecting other DUBs. Recently, ML323 effectively suppressed the proliferation of HCC and ESCC cells, inhibited TGF‐β induced EMT in TNBC cells, and induced apoptosis and DNA damage in ESCC cells, demonstrating its potential as a viable anticancer treatment.^430^

Through a scaffold hopping strategy to enhance the cellular activity of FT671 (known USP7 inhibitor), resulting in the discovery of YCH2823 as a novel and potent USP7 inhibitors. YCH2823 demonstrated potent inhibition of USP7, inhibiting cancer cell growth, promoting G1 phase arrest and apoptosis, increasing p53 and p21 levels, and showing synergistic effects with mTOR inhibitors, making it a promising anticancer agent for both TP53 wild‐type and mutant tumors.^432^ Recently, a series of pyrrole[2,3‐d]pyrimidin‐4‐one derivatives were designed for USP7 inhibition, with the representative compound Z33 (YCH3124) showing high potency and selectivity. YCH3124 exhibited strong antiproliferative activity across various cancer cell lines, inhibited the USP7 pathway, led to the accumulation of p53 and p21, disrupted cell cycle progression at G1 phase, and induced significant apoptosis, highlighting its potential as a potent anticancer agent.^433^ Moreover, a series of 2‐aminopyridine derivatives were designed and synthesized based on structural modifications of the lead USP7 inhibitor, GNE6640. Compounds 7 showed the most potential, with IC_50_ values of 7.6 µM, promoting MDM2 degradation, p53 stabilization, and p21 expression, though they displayed moderate antiproliferative activities against HCT116 cells.^434^ Furthermore, the proteolysis targeting chimera (PROTAC) strategy was employed to discovery small molecule to degrade USP7 for inhibiting its function. To design PROTAC targeting USP7, known USP7 ligand molecule FT671 and XL188 were optimized by modifying the right‐hand side tail groups to enhance target binding affinity and lipophilicity. Among the synthesized compounds, PROTAC 17 exhibited the most potent USP7 inhibition with an IC_50_ value of 1.7 µM, aligning with docking results and cell viability profiles, indicating its potential efficacy.^435^

A novel compound D1 with quinolin‐4(1H)‐one scaffold was identified as a lead USP10 inhibitor due to its high potency and selectivity. D1 significantly inhibited HCC cell proliferation and clone formation by targeting the ubiquitin pathway, promoting YAP degradation, downregulating p53 and p21, arresting cells in S‐phase, and inducing apoptosis, demonstrating its potential for USP10‐positive HCC treatment.^436^ Spautin‐1 has been reported as an inhibitor of USP10 and USP13, significantly reducing the proliferation and migration of glioblastoma cells. Mechanistically, Spautin‐1 disrupts the RAF–ERK pathway and glycolysis, and decreases the proto‐oncogene SKP2, highlighting its potential to counteract key mechanisms involved in glioblastoma progression.^438^

Though high‐throughput screening and structure‐based optimization, BAY‐805 was developed as a highly potent and selective noncovalent USP21 inhibitor. BAY‐805 shows the low nanomolar affinity and robust target engagement, leading to strong NF‐κB activation, representing a valuable chemical probe for exploring USP21 biology.^439^ Utilizing predictive models to screen and identify potential compounds from the United States Food and Drug Administration (US FDA)‐approved drug library, nifuroxazide was identified as the most potent USP21 inhibitor with an IC_50_ of 14.9 µM. Nifuroxazide demonstrated efficacy in inhibiting USP21, modulating its substrate ACLY, increasing p‐AMPKα levels, and elevating miR‐4458, both in HepG2 cells and in vivo, highlighting its potential for treating metabolic disorders and cancer proliferation.^440^ Based on computer‐aided drug design methodologies, several potential ligands were designed to target the catalytic domain of USP21, as a result, MOLHYB‐0436 showed the best binding affinity and stability. MOLHYB‐0436 can disrupt the Wnt signaling pathway, which is activated by USP21 in PDAC, displaying promising pharmacokinetic and pharmacodynamic profiles, good bioavailability, and low toxicity, indicating its potential as a treatment for pancreatic cancer.^441^ Recently, identifying a traditional Chinese secoiridoid compound, gentiopicroside, was identified as a USP22 inhibitor by stably binding to the USP22 catalytic pocket. Gentiopicroside decreased Foxp3 expression and Treg immune suppressive activity, increased histone 2B monoubiquitination, and decreased PD‐L1 expression in cancer cells, enhancing antitumor immunity. In vivo studies showed that gentiopicroside significantly inhibited the growth of lung adenocarcinoma in mice and increased CD8+ T cell production of IFN‐γ and granzyme B.^442^

USP25 and USP28, which are highly homologous and associated with diseases like cancer and neurodegenerative disorders, have recently become focal points for the development of therapeutic inhibitors.^466^, ^467^ Through screening a synthetic compound library and further structure optimization, CT1113 was synthesized and developed as a promising USP inhibitor with potent activity against USP28 and USP25. CT1113 can reduce c‐MYC levels and inhibit USP28 and USP25 in various cancer cell lines, resulting in decreased expression of MYC–MAX target genes and increased ubiquitination of substrates like c‐MYC and Tankyrase, thus exhibiting a broad‐spectrum anticancer activity.^443^ Moreover, Vismodegib was identified as a lead structure for USP28 inhibition by screening a drug library, and further structure‐based optimization led to the discovery of a more potent derivative 9p. Compound 9p showed high selectivity and potency in inhibiting USP28, causing cytotoxicity in colorectal and lung cancer cells, and enhancing CRC cell sensitivity to Regorafenib. Compound 9p also downregulated c‐MYC via the ubiquitin–proteasome system, highlighting its potential anticancer effects through USP28 inhibition.^444^

Given the similar conserved catalytic domain structures in the USP family, different USP targeted inhibitors can also serve as a starting point for ligand design. For example, USP7 and USP47 are closely related in systematics and functionally perform some similar biological functions. The study of the biochemical and structural characteristics of USP47 reveals why the USP7 inhibitor derivative P50429 has targeting and inhibitory abilities toward USP47, and provides new insights for further design of USP47 specific inhibitors.^445^ To develop USP48 inhibitors, 14 commercially available USP inhibitors were screened and ML364 (known as a USP2 inhibitor) was identified as a candidate for further optimization. Using a ligand‐based approach, a series of ML364 analogs were synthesized, with compound 17e showing an IC_50_ of 12.6 µM in inhibiting USP48 activity. Further structural refinements are necessary to enhance the inhibition activity and specificity of these compounds.^446^

### 6.2 Targeting OTUs

Recent advancements in OTU deubiquitinase inhibitors have opened new avenues for targeted cancer therapy by modulating deubiquitination processes to suppress tumor growth. Studies have reported that various small‐molecule compounds, such as SJB3‐019A, OTUB2‐IN‐1, and OTUDin3, exhibit significant inhibitory effects on deubiquitinases along with notable antitumor activities. This section primarily explores the screening processes, structural optimization, and application potential of several representative OTU deubiquitinase inhibitors across different tumor models, underscoring the importance of further studies to enhance drug selectivity and safety.

Screening was conducted on 20 reported databases of DUB or DUB‐like protease inhibitors, and two USP1‐like inhibitors, SJB2‐043 and SJB3‐019A, were found to exhibit significant inhibitory effects on OTUB1 and USP8 at a concentration of 2.5 µM. To further explore the potential of these inhibitors, in‐depth structure‐activity relationship studies were conducted. Four analogues, compounds 60, 61, 62, and 63, were developed by modifying the 2‐phenyl ring of SJB2‐043 with single substitution, double substitution, and phenyl ring modification. Subsequently, protein thermal stability and enzyme activity experiments of compound 61 on OTUB1, as well as molecular docking mechanism analysis, showed that compound 61 has high selectivity and strong inhibitory effects on OTUB1 and USP8. This compound not only demonstrated strong inhibitory effects on OTUB1 and USP8 in vitro but also showed significant antiproliferative effects in H1975 and A549 NSCLC cell lines. Additionally, compound 61 effectively inhibited tumor growth in the H1975 xenograft mouse model after low‐dose intraperitoneal administration. However, despite the significant antitumor activity exhibited by compound 61, studies also found that the OTUB1/USP8 protein exhibits instability when bound to compound 61. This discovery suggests that further validation of the binding mode of inhibitors is needed in the future, and their efficacy and safety should be evaluated in other animal models. In particular, in‐depth studies of the pharmacokinetic and pharmacological properties of compound 61, as well as its mechanisms of action in different tumor types, will help optimize its structure, improving its stability and selectivity.^447^

As the first effective OTUB2 inhibitor, OTUB2‐IN‐1 forms hydrogen bonds between its two carboxyl groups and four amino acid residues (Thr45, Gly49, Ser223, and His224) in the OTUB2 catalytic pocket, significantly inhibiting the hydrolysis activity of the recombinant GST‐OTUB2 protein toward substrate Ub‐R110. In the mouse LL/2 tumor model, OTUB2‐IN‐1 treatment significantly inhibited tumor growth, prolonged mouse survival, and exhibited no significant toxicity. Subsequent animal model experiments and immunohistochemical analysis showed that OTUB2‐IN‐1 also demonstrated therapeutic potential in mouse B16‐F10 and KLN205 tumor models. Additionally, tumor infiltration of CD8 and GZMB CTLs was significantly increased in OTUB2‐IN‐1‐treated mouse tumors, while the expression of PD‐L1 was significantly reduced. This result indicates that OTUB2‐IN‐1 exerts its antitumor immune effect by degrading PD‐L1. In summary, OTUB2‐IN‐1 exhibits significant antitumor potential by precisely targeting DUBs to regulate tumor immune escape mechanisms.^448^

In the study of OTUD3, computer‐aided virtual screening and biological experiments identified two OTUD3 inhibitors, OTUDin3 and Rolapitant, which inhibit the deubiquitinase activity of OTUD3. Molecular docking analysis shows that OTUDin3 forms various interactions with OTUD3, including hydrophobic interactions at different sites, hydrogen bonding, and π–π stacking interactions. In vitro experiments demonstrated that OTUDin3 can inhibit the growth, migration, and invasion of H1299 cell lines and induce cell apoptosis by targeting OTUD3. Rolapitant significantly inhibited the proliferation of NSCLC cell lines such as A549, H1299, and H460. In the in vivo validation experiment, OTUDin3 significantly inhibited tumor growth in a nude mouse model carrying H1299 tumors, with no significant health issues or weight loss observed. Rolapitant showed significant effects in inhibiting tumor growth in nude mouse models carrying A549 tumors, especially when used in combination with targeted drugs. These research findings further confirm the effectiveness of strategies to influence tumor growth and cell death by regulating specific DUBs.^449^, ^450^

Additionally, effective inhibitors 7Ai and 7Bi targeting OTUD7A and OTUD7B were discovered using AtomNet virtual screening technology. First, a homologous model of the OTUD7A‐OTU domain was generated based on the available crystal structures of closely related OTUD7B‐OTU domains. Using the generated structures, 4 million compounds were screened, and based on their ability to reduce the abundance of EWS‐FLI1 protein in A673 and SK‐N‐MC cells, it was found that 7Ai can effectively block OTUD7A‐mediated deubiquitination of EWS‐FLI1 without affecting the binding of OTUD7A to EWS‐FLI1. Preliminary studies have shown that 7Ai treatment can significantly reduce the proliferation and migration ability of A673 cells. Although this study lacks formal in vivo experiments, this discovery provides more possibilities for exploring the combination therapy of 7Ai and active chemotherapy drugs in the future. Similarly, 10 candidate compounds for OTUD7B inhibitors were screened through AtomNet technology and biological experiments. Subsequently, the inhibitory effects of these compounds on Akt pS473 signaling in various NSCLC cell lines were investigated, and OTUD7B deubiquitinase activity was measured in vitro using K11‐linked dimeric ubiquitin (di‐ub) as a substrate. Finally, the study found that compound 7Bi specifically inhibits OTUD7B and inhibits the growth of various NSCLC cells, including A549, in vitro. The discovery of 7Ai and 7Bi inhibitors indicates that AtomNet has significant potential in discovering different inhibitors of closely related deubiquitinase members.^451^, ^452^

Although there is limited research on the role of TRABID in tumors, computer screening of crystal structures based on the A20 OTU domain, searching for compounds that may bind to TRABID catalytic sites, combined with preliminary biological experiments, identified NSC112200 and NSC267309 as TRABID inhibitors with IC_50_ values of approximately 3 µM. At a concentration of 20 µM, these two compounds not only inhibited the growth of colorectal tumor cell lines HCT‐116 and SW480 but also inhibited the growth of two abnormal cell lines, NIH3T3 and HEK293.^453^ Subsequently, Zhang et al.^454^ conducted a more in‐depth study on the mechanism of action of the inhibitor NSC112200. The results showed that NSC112200 could inhibit the deubiquitinase activity of ZRANB1, promote the degradation of EZH2 protein, and effectively kill TNBC cells at micromolar concentrations. However, the inhibitor exhibits certain toxicity in mice, so its structure needs further optimization.^454^ These studies indicate that NSC112200 and NSC267309 have significant antitumor potential as TRABID inhibitors. Further optimizing the structure of these inhibitors not only helps to improve their selectivity and safety but also provides important theoretical and experimental bases for the development of new‐generation TRABID inhibitors.

To accelerate the discovery of OTU family inhibitors, an efficient screening platform was established by designing a chemical library based on the chemical types of various DUB inhibitors and combining it with activity‐based protein mass spectrometry analysis. Through this platform, the inhibitor of VCPIP1, the fluoroquinolone compound CAS‐12290‐201, was successfully screened. This compound exhibits high selectivity and strong inhibitory effect on VCPIP1, with an enzymatic IC_50_ value of 70 nM. Additionally, cell experiments have shown that CAS‐12290‐201 has low toxicity and good target binding ability. This study demonstrates the feasibility of developing DUB inhibitors through structure‐guided methods. Specifically, the successful application of this virtual platform demonstrates that activity‐based protein mass spectrometry analysis and well‐designed chemical libraries can effectively screen and optimize DUB inhibitors. This method not only improves screening efficiency but also significantly enhances the specificity and efficacy of inhibitors, providing important references and tools for future drugdevelopment.^455^

In addition to developing inhibitors that directly interact with catalytic site domains, a novel strategy for creating heterobifunctional chimeric molecules targeting DUBs has recently been proposed, known as DUBTAC technology. This technology aims to form chimeric molecules that target specific proteins for degradation in a ubiquitin‐dependent manner, thereby improving selectivity and intracellular efficiency. For instance, a recently developed covalent small molecule ligand for OTUB1, acrylamide EN523, can specifically target the noncatalytic allosteric Cys C23 on OTUB1 and effectively bind to OTUB1 within cells. This binding method does not inhibit the deubiquitination activity of OTUB1; instead, it interacts with OTUB1 through a novel mechanism, highlighting the potential role and application prospects of DUBTAC technology in the future development of OTU‐targeted drugs.^468^ The advancement of this technology provides a new strategy for treating diseases such as cancer and is expected to play a significant role in the field of precision medicine.

### 6.3 Targeting other DUBs

Significant advancements in the development of DUB inhibitors have expanded the scope beyond the well‐studied USP and OTU families, leading to the discovery of potent and selective inhibitors targeting other DUB family members. This section highlights key compounds such as GK13S, a selective inhibitor of UCHL1, VLX1570 targeting USP14 and UCHL5, and the Rpn11 inhibitor capzimin. These inhibitors showcase high potency and specificity, serving as essential tools for biochemical research and potential therapeutic applications.

One notable example is the UCHL1‐targeted inhibitor GK13S. This 3‐carboxy‐N‐cyanopyrrolidine probe exhibits an IC_50_ value of 50 nM, demonstrating high potency. The cocrystal structure reveals that GK13S binds to UCHL1 in a hybrid conformation, partaking in both the apo and ubiquitin‐bound states. This inhibitor covalently attaches to the C90 residue via its cyanamide warhead and occupies the enzyme's catalytic cleft. Specificity is conferred by the positioning of the pyrrolidine moiety in a unique pocket exclusive to UCHL1's apo conformation. Additionally, although GK13S primarily targets UCHL1, it also shows binding affinity for PARK7 and its family member C21orf33. Remarkably, GK13S effectively inhibits UCHL1 activity in cellular contexts without compromising the viability of HEK293 and U‐87 MG cells.^456^

Furthermore, VLX1570, a dual inhibitor of USP14 and UCHL5, was developed from b‐AP15. Exhibiting greater drug‐like properties, VLX1570 has enhanced potency and solubility in aqueous solutions.^457^ Specifically, VLX1570 shows superior activity in hydrolysis assays using Ub‐AMC as a substrate, yielding an IC_50_ of 13.0 µM compared with b‐AP15's 16.8 µM.^458^ In Waldenstrom macroglobulinemia (WM) cells, targeting USP14 and UCHL5 with VLX1570 induces apoptosis and leads to the accumulation of high molecular weight polyubiquitinated protein conjugates.^459^ Additionally, in vivo studies report a significant reduction in tumor burden and extended survival in WM‐xenografted mice following a 21‐day VLX1570 treatment regimen. Although VLX1570 is the first DUB inhibitor to enter clinical trials, the study was terminated due to dose‐limiting toxicities observed at higher doses.^460^

Another significant discovery is iBAP, a selective inhibitor of BAP1, with an IC_50_ value of 253 nM. This inhibitor exhibits high specificity for BAP1 over other DUBs. Research shows that leukemia cells harboring truncated ASXL1 gain‐of‐function mutations, such as K562 cells and THP1 cells, are particularly sensitive to iBAP treatment. RNA‐seq analysis indicates that iBAP downregulates leukemia‐associated genes including HMGN5, STAT5A, HOXA11, TWIST1, and MBD2. In vivo studies corroborate iBAP's efficacy, showing delayed progression in leukemia and improved survival in mice models bearing ASXL1 mutations.^461^

Rpn11 inhibition has also made strides with the identification of 8‐thioquinoline (8TQ) and its optimized derivative, capzimin. 8TQ initially exhibited strong Rpn11 inhibition through chelating the Zn^2+^ ion at the enzyme's active site, with an IC_50_ of 2.5 µM. However, its lack of specificity prompted further optimization, resulting in capzimin, which shows potent inhibition of Rpn11 with an IC_50_ of 0.34 µM and high selectivity over other JAMMs. Capzimin effectively inhibits cell proliferation and induces apoptosis in various cancer cell lines, highlighting its potential as a therapeutic agent.

Targeting CSN5 with siRNA has been shown to halt cell cycle progression and induce apoptosis in HCC cells, making CSN5 a compelling therapeutic target. Utilizing a specialized high‐throughput screening platform, CSN5i‐3 emerged as a highly potent inhibitor. CSN5i‐3 inhibits CSN‐mediated CRL deneddylation with an exceptionally low IC_50_ of 0.0058 µM and exhibits favorable pharmacokinetic properties. Its selectivity is noteworthy, with minimal inhibition observed for other JAMMs such as AMSH‐LP (IC_50_ > 100 µM) and RPN11 (IC_50_ = 53 µM). In tests on 500 diverse cancer cell lines, CSN5i‐3 showed a range of inhibitory activities and significantly suppressed the growth of human xenografts in mouse models. These findings position CSN5i‐3 as a promising candidate for treating cancers driven by CSN5 overexpression.^463^

Recent progress in developing inhibitors for various DUBs beyond the USP and OTU families underscores significant advancements in this field. Compounds such as GK13S, VLX1570, iBAP, capzimin, and CSN5i‐3 demonstrate high potency and specificity, providing valuable tools for biochemical research and potential therapeutic applications. These inhibitors not only enhance our understanding of DUB‐related cellular mechanisms but also offer promising prospects for treating cancers and other diseases associated with dysregulated ubiquitination pathways. Continued research in this area is likely to yield even more potent and specific inhibitors with broad therapeutic potential.

Although significant progress has been achieved in developing inhibitors for the entire DUB family, substantial challenges persist in this area of research. A major obstacle is the identification of potent compounds that selectively target the catalytic pocket of DUBs. Furthermore, the dynamic alternation between active and inactive conformations of DUBs complicates the design of predictive biochemical assays and the development of drug‐like compounds. Additionally, the ubiquitin transfer mediated by reactive thiols in DUBs presents difficulties in the screening of inhibitors. These challenges have limited the advancement of DUB inhibitors, with only a few making preliminary progress in clinical trials for cancer treatment.

Mitoxantrone, approved by the US FDA as a USP11 inhibitor, is undergoing numerous phase I/II trials targeting conditions including neoplasms, acute myeloid leukemia, MS, and breast cancer.^469^ A notable 2014 study (Clinical Trial ID: NCT02043756) assessed its pharmacokinetics and safety in 20 solid tumor patients, with doses up to 18 mg/m^2^ shown to be effective and well‐tolerated. CSPC ZhongQi Pharmaceutical Technology is conducting a phase II trial (Clinical Trial ID: NCT03776279) to evaluate its efficacy in 106 patients with refractory T‐cell lymphomas. Additionally, the University of Pittsburgh initiated a study (Clinical Trial ID: NCT03839446) in 2019 to assess Mitoxantrone combined with etoposide and gemtuzumab ozogamicin for acute myeloid leukemia.

VLX1570 demonstrated promising antimyeloma effects at doses of 0.6 mg/kg or higher. However, its development faced setbacks due to severe respiratory complications at elevated doses, leading to the study's discontinuation, with questions remaining about the role of its formulation in these adverse effects.^460^

Pimozide is being evaluated in phase II trials at the University of Calgary for amyotrophic lateral sclerosis (ALS). The first trial (Clinical Trial ID: NCT02463825) focuses on 25 patients with neuromuscular junction dysfunction, while a second placebo‐controlled trial (Clinical Trial ID: NCT03272503) involves 100 participants to explore its potential in slowing ALS progression.

To advance DUB inhibitors toward clinical application, future research should pivot toward gaining deeper biological insights. While traditional methods involve screening known libraries and identifying hit compounds, a shift is needed toward confirming target engagement in cellular and in vivo models through biomarker studies, gene knockout phenotypes, and competitive binding assays. Chemical proteomics is indispensable in uncovering novel DUB functions and substrate interactions, capitalizing on the unique adaptability, dose responsiveness, and selectivity of small molecules. Beyond simple validation, attention should be given to exploring new biological roles of DUBs using quantitative proteomics, transcriptomics, and phenotypic analyses in various cell types.

## 7 DISCUSSIONS AND PERSPECTIVES

As our understanding of ubiquitin signaling advances, the critical role of DUBs in this process becomes increasingly apparent. In both health and disease, DUB families—including USP, OTU, UCH, MJD, and JAMM—exhibit distinct functions and mechanisms. The USP family, in particular, is broadly involved in various cellular processes, while other families such as OTU demonstrate specificity in chain preferences, significantly impacting tumor‐associated signaling pathways.^5^, ^470^, ^471^ Additionally, some DUBs have nonclassical functions; for example, OTUB1 influences ubiquitin‐conjugating enzyme E2 activity and A20 exhibits both deubiquitination and E3 ligase activities.^30^, ^472^ Understanding the roles of different DUB family members in various cellular processes is essential for elucidating their fundamental mechanisms in health and disease. This knowledge also paves the way for novel precision drug development. By identifying the specific pathogenesis associated with different DUBs, researchers can design small molecule inhibitors or activators tailored to precisely modulate deubiquitination activities. This approach has the potential to create highly selective therapeutic agents that restore normal cellular functions and impede disease progression. In conclusion, the diverse functions and unique regulatory mechanisms of DUBs in health and disease offer critical insights into their roles in human diseases and provide innovative strategies for developing targeted therapies. Future research should further investigate their comprehensive biological functions to uncover new regulatory mechanisms and potential therapeutic targets, thereby enhancing the ability to design effective treatment strategies.

The dysfunction of the DUBs plays a critical role in tumorigenesis and presents significant implications for cancer research and therapy. Abnormal activation of DUBs is frequently observed in various cancers, underscoring their pivotal role in tumor initiation, progression, and metastasis. These enzymes are integrated into key signaling pathways such as NF‐κB and PI3K/Akt/mTOR, essential for regulating cellular processes critical to cancer development. For instance, OTUB1 and OTULIN modulate the NF‐κB pathway through the deubiquitination of key molecules like p105/p100 and NEMO, thus intricately controlling cell survival and immune responses.^473^, ^474^ Overexpression of DUBs is associated with the onset and progression of several cancers, suggesting their role as potential tumor promoters. However, DUBs members act as tumor suppressors. For example, OTUB1 can inhibit breast cancer growth and invasiveness via nonclassical mechanisms, while OTUD6B can suppress liver cancer metastasis independently of its enzymatic activity.^256^, ^475^ The dual role of OTU members in promoting or suppressing tumorigenesis highlights their complex involvement in cancer biology and potential as biomarkers and therapeutic targets. Understanding their interactions with substrates and adaptation to TMEs can elucidate mechanisms behind their tumor‐promoting or suppressing functions, fostering new therapeutic strategies to disrupt pathological pathways or restore normal cellular functions. Despite these promising directions, several challenges persist. The complexity of the substrates involved and the specific TMEs contribute to the elusive nature of DUB regulation in cancer. Detailed studies focusing on the context‐specific roles of DUBs are necessary to unravel their precise functions and therapeutic potentials. Additionally, developing selective inhibitors or activators that target specific DUB members could offer more precise and effective cancer therapies. Integrating multiomics data to identify differential expression patterns and mutations in DUB genes across various cancers could improve their application as biomarkers and inform targeted treatment strategies. Overall, the dysfunction of DUBs in cancer provides profound insights into tumor biology and offers avenues for innovative therapeutic approaches. By leveraging the unique regulatory capabilities of DUBs, future research can significantly advance cancer treatment and improve patient outcomes.

DUBs are a diverse family of enzymes that play crucial roles in a broad spectrum of cellular processes by removing ubiquitin from target proteins, thereby modulating their degradation, activity, and localization. This deubiquitination function places DUBs at the center of several pathophysiological processes, making them compelling targets for therapeutic intervention across various diseases, including neurodegenerative disorders, cardiovascular diseases, inflammatory disorders, and developmental diseases. Continued research will likely emphasize a more profound understanding of individual DUB roles and mechanisms, paving the way for targeted therapies. As the functionality and regulation of DUBs are intricate and disease‐specific, future directions may involve precision medicine approaches, utilizing biomarkers to tailor DUB‐focused therapies that minimize side effects while maximizing therapeutic efficacy.

The development of small molecule inhibitors targeting DUBs faces significant challenges due to the highly conserved catalytic domains of each subfamily and complex regulatory mechanisms. These factors make achieving specificity and selectivity in inhibitors particularly difficult. Despite the identification of several inhibitors for OTUs like OTUB1, OTUB2, OTUD7B, and TRABID, challenges related to activity, selectivity, pharmacokinetics, and safety persist, leading to suboptimal drug performance.^447^, ^448^, ^452^, ^454^ However, the advent of artificial intelligence technologies such as AtomNet and AlphaFold offers promising avenues for drug design. These tools facilitate virtual screening and optimization of candidate drugs and enable precise prediction of protein structures, aiding in the design of small molecules that accurately target DUBs. Additionally, the therapeutic potential of DUB activators should not be overlooked. Some DUBs exhibit tumor suppressive functions, and developing small molecule activators that enhance the catalytic activity or stability of these tumor suppressive DUBs may provide a novel approach for cancer treatment. Future research should focus on elucidating the specific roles of individual DUB members in cancer progression and harnessing advanced computational and biochemical techniques to develop targeted therapies with improved efficacy and safety profiles. PROTACs represent a novel technology that induces selective protein degradation via the ubiquitin–proteasome system, offering a new strategy for drug development. Designing PROTACs for selective degradation of OTUs offers the possibility of achieving more effective and sustained inhibition of their carcinogenic activity, potentially overcoming compensatory mechanisms commonly found in traditional inhibitors. Building on this concept, DUBTAC technology has emerged, achieving higher selectivity and stability through covalent binding, thereby reducing off‐target effects. For example, the covalent small molecule ligand acrylamide EN523 of OTUB1 is the first developed DUBTAC and holds significant promise. The structural characteristics of OTUB1 make it an ideal target for DUBTAC technology.^468^ By covalently binding small molecule ligands, it does not affect the deubiquitination activity of OTUB1, demonstrating the potential role and application prospects of DUBTAC technology in the future development of OTU‐targeted drugs. In conclusion, while the development of small molecules targeting DUBs presents several challenges, the integration of AI technologies and novel approaches like PROTACs and DUBTACs provide promising strategies. Future research should aim to address issues of specificity, selectivity, and safety to harness the full potential of these therapeutic agents in clinical applications.

In summary, this review elucidates the structural determinants of substrate specificity and catalytic mechanisms of the DUBs family, emphasizing their functional diversity in various cellular processes. We detail the key roles of DUBs in regulating signaling cascades such as NF‐κB, PI3K/AKT/mTOR, and MAPK pathways, providing insights into their molecular mechanisms. Additionally, we explore the dual role of DUBs members in tumorigenesis, functioning as both tumor inhibitors and oncogenes in different types of cancer. Importantly, we summarize current and emerging treatment strategies targeting the DUBs, including small molecule inhibitors, PROTACs, DUBTACs, and potential combination therapies. This review aims to deepen our understanding of the key roles of DUBs in cellular homeostasis and signaling, laying the foundation for novel diagnostic tools and therapeutic strategies. Future in‐depth research on DUB‐related mechanisms and targeted drug development holds significant potential for revealing new regulatory pathways and innovative treatments for various diseases.

## AUTHOR CONTRIBUTIONS

Yali Xian and Jing Ye conceived the manuscript's idea and structure, conducted the literature review, and drafted the initial manuscript. Along with Gu He, they discussed the manuscript's concepts and refined the review's main arguments and focus. Nan Zhang was primarily responsible for illustrating and drawing the figures included in the manuscript. Yu Tang and Cheng Peng collected relevant information from various sources, summarized the data, and compiled the tables that are integral to the manuscript's discussion and analysis. Wei Huang and Gu He provided valuable discussions that enhanced the depth and scope of the manuscript. They critically reviewed and revised the manuscript for important intellectual content, ensuring clarity, coherence, and scientific accuracy. All authors read and approved the final version of the manuscript.

## CONFLICT OF INTEREST STATEMENT

The authors declare that there are no conflict of interest.

## ETHICS STATEMENT

Not applicable.

## Data Availability

Not applicable.

## References

[1] Lee JM , Hammarén HM , Savitski MM , Baek SH . Control of protein stability by post‐translational modifications. Nat Commun. 2023;14(1):201. 36639369 10.1038/s41467-023-35795-8 PMC9839724

[2] Mennerich D , Kubaichuk K , Kietzmann T . DUBs, hypoxia, and cancer. Trends Cancer. 2019;5(10):632‐653. 31706510 10.1016/j.trecan.2019.08.005

[3] Sun T , Liu Z , Yang Q . The role of ubiquitination and deubiquitination in cancer metabolism. Mol Cancer. 2020;19(1):146. 33004065 10.1186/s12943-020-01262-x PMC7529510

[4] Harrigan JA , Jacq X , Martin NM , Jackson SP . Deubiquitylating enzymes and drug discovery: emerging opportunities. Nat Rev Drug Discovery. 2018;17(1):57‐78. 28959952 10.1038/nrd.2017.152 PMC7097658

[5] Dewson G , Eichhorn PJA , Komander D . Deubiquitinases in cancer. Nat Rev Cancer. 2023;23(12):842‐862. 37935888 10.1038/s41568-023-00633-y

[6] Lange SM , Armstrong LA , Kulathu Y . Deubiquitinases: from mechanisms to their inhibition by small molecules. Mol Cell. 2022;82(1):15‐29. 34813758 10.1016/j.molcel.2021.10.027

[7] Cheng J , Guo J , North BJ , et al. Functional analysis of deubiquitylating enzymes in tumorigenesis and development. Biochim Biophys Acta Rev Cancer. 2019;1872(2):188312. 31449841 10.1016/j.bbcan.2019.188312

[8] Fang YZ , Jiang L , He Q , Cao J , Yang B . Deubiquitination complex platform: a plausible mechanism for regulating the substrate specificity of deubiquitinating enzymes. Acta Pharm Sin B. 2023;13(7):2955‐2962. 37521861 10.1016/j.apsb.2023.02.019 PMC10372820

[9] Mevissen TE , Hospenthal MK , Geurink PP , et al. OTU deubiquitinases reveal mechanisms of linkage specificity and enable ubiquitin chain restriction analysis. Cell. 2013;154(1):169‐184. 23827681 10.1016/j.cell.2013.05.046 PMC3705208

[10] Wang Q , Liang S , Qian J , et al. OTUD1 promotes isoprenaline‐ and myocardial infarction‐induced heart failure by targeting PDE5A in cardiomyocytes. Biochim Biophys Acta Mol Basis Dis. 2024;1870(3):167018. 38185350 10.1016/j.bbadis.2024.167018

[11] Jia F , Li H , Jiao Q , et al. Deubiquitylase OTUD3 prevents Parkinson's disease through stabilizing iron regulatory protein 2. Cell Death Dis. 2022;13(4):418. 35490179 10.1038/s41419-022-04704-0 PMC9056525

[12] Hertens P , van Loo G . A20: a jack of all trades. Trends Cell Biol. 2024;34(5):360‐362. 38461099 10.1016/j.tcb.2024.02.008

[13] Liu L , Pang J , Qin D , et al. Deubiquitinase OTUD5 as a novel protector against 4‐HNE‐triggered ferroptosis in myocardial ischemia/reperfusion injury. Adv Sci (Weinh). 2023;10(28):e2301852. 37552043 10.1002/advs.202301852 PMC10558642

[14] Zhou N , Qi H , Liu J , et al. Deubiquitinase OTUD3 regulates metabolism homeostasis in response to nutritional stresses. Cell Metab. 2022;34(7):1023‐1041. 35675826 10.1016/j.cmet.2022.05.005

[15] Hu M , Li P , Li M , et al. Crystal structure of a UBP‐family deubiquitinating enzyme in isolation and in complex with ubiquitin aldehyde. Cell. 2002;111(7):1041‐1054. 12507430 10.1016/s0092-8674(02)01199-6

[16] Makarova KS , Aravind L , Koonin EV . A superfamily of archaeal, bacterial, and eukaryotic proteins homologous to animal transglutaminases. Protein Sci. 1999;8(8):1714‐1719. 10452618 10.1110/ps.8.8.1714 PMC2144420

[17] Komander D , Clague MJ , Urbé S . Breaking the chains: structure and function of the deubiquitinases. Nat Rev Mol Cell Biol. 2009;10(8):550‐563. 19626045 10.1038/nrm2731

[18] Luo J , Ruan X , Huang Z , et al. Structural basis for the dual catalytic activity of the Legionella pneumophila ovarian tumor (OTU) domain deubiquitinase LotA. J Biol Chem. 2022;298(10):102414. 36007613 10.1016/j.jbc.2022.102414 PMC9486567

[19] García‐Santisteban I , Peters GJ , Giovannetti E , Rodríguez JA . USP1 deubiquitinase: cellular functions, regulatory mechanisms and emerging potential as target in cancer therapy. Mol Cancer. 2013;12:91. 23937906 10.1186/1476-4598-12-91 PMC3750636

[20] Rougé L , Bainbridge TW , Kwok M , et al. Molecular understanding of USP7 substrate recognition and C‐terminal activation. Structure (London, England : 1993). 2016;24(8):1335‐1345. 27452404 10.1016/j.str.2016.05.020

[21] Molland K , Zhou Q , Mesecar AD . A 2.2 Å resolution structure of the USP7 catalytic domain in a new space group elaborates upon structural rearrangements resulting from ubiquitin binding. Acta Crystallogr F Struct Biol Commun. 2014;70:283‐287. Pt 3. 24598911 10.1107/S2053230X14002519 PMC3944686

[22] Faesen AC , Dirac AM , Shanmugham A , Ovaa H , Perrakis A , Sixma TK . Mechanism of USP7/HAUSP activation by its C‐terminal ubiquitin‐like domain and allosteric regulation by GMP‐synthetase. Mol Cell. 2011;44(1):147‐159. 21981925 10.1016/j.molcel.2011.06.034

[23] Shi Y , Chen X , Elsasser S , et al. Rpn1 provides adjacent receptor sites for substrate binding and deubiquitination by the proteasome. Science. 2016;351(6275):aad9421. 26912900 10.1126/science.aad9421 PMC4980823

[24] Gao H , Yin J , Ji C , et al. Targeting ubiquitin specific proteases (USPs) in cancer immunotherapy: from basic research to preclinical application. J Exp Clin Cancer Res. 2023;42(1):225. 37658402 10.1186/s13046-023-02805-y PMC10472646

[25] Makarova KS , Aravind L , Koonin EV . A novel superfamily of predicted cysteine proteases from eukaryotes, viruses and Chlamydia pneumoniae. Trends Biochem Sci. 2000;25(2):50‐52. 10664582 10.1016/s0968-0004(99)01530-3

[26] Reyes‐Turcu FE , Ventii KH , Wilkinson KD . Regulation and cellular roles of ubiquitin‐specific deubiquitinating enzymes. Annu Rev Biochem. 2009;78:363‐397. 19489724 10.1146/annurev.biochem.78.082307.091526 PMC2734102

[27] Craighead JM . Dunnigan's commentary. The role of chrysotile in the pathogenesis of mesothelioma. Am J Ind Med. 1988;14(2):241‐243. Response to Dr. 2849872 10.1002/ajim.4700140221

[28] Suresh HG , Pascoe N , Andrews B . The structure and function of deubiquitinases: lessons from budding yeast. Open Biol. 2020;10(10):200279. 33081638 10.1098/rsob.200279 PMC7653365

[29] Wiener R , Zhang X , Wang T , Wolberger C . The mechanism of OTUB1‐mediated inhibition of ubiquitination. Nature. 2012;483(7391):618‐622. 22367539 10.1038/nature10911 PMC3319311

[30] Juang YC , Landry MC , Sanches M , et al. OTUB1 co‐opts Lys48‐linked ubiquitin recognition to suppress E2 enzyme function. Mol Cell. 2012;45(3):384‐397. 22325355 10.1016/j.molcel.2012.01.011 PMC3306812

[31] Wu M , Sun L , Song T . OTUB1‐mediated inhibition of ubiquitination: a growing list of effectors, multiplex mechanisms, and versatile functions. Front Mol Biosci. 2023;10:1261273. 38264570 10.3389/fmolb.2023.1261273 PMC10803509

[32] Wertz IE , O'Rourke KM , Zhou H , et al. De‐ubiquitination and ubiquitin ligase domains of A20 downregulate NF‐kappaB signalling. Nature. 2004;430(7000):694‐699. 15258597 10.1038/nature02794

[33] Pan X , Wu S , Wei W , Chen Z , Wu Y , Gong K . Structural and functional basis of JAMM deubiquitinating enzymes in disease. Biomolecules. 2022;12(7):910. 35883466 10.3390/biom12070910 PMC9313428

[34] Zeng C , Zhao C , Ge F , et al. Machado‐Joseph deubiquitinases: from cellular functions to potential therapy targets. Front Pharmacol. 2020;11:1311. 32982735 10.3389/fphar.2020.01311 PMC7479174

[35] Xu Z , Zhang N , Shi L . Potential roles of UCH family deubiquitinases in tumorigenesis and chemical inhibitors developed against them. Am J Cancer Res. 2024;14(6):2666‐2694. 39005671 10.62347/OEGE2648 PMC11236784

[36] Louie BH , Kurzrock R . BAP1: not just a BRCA1‐associated protein. Cancer Treat Rev. 2020;90:102091. 32877777 10.1016/j.ctrv.2020.102091 PMC7655689

[37] Mevissen TET , Komander D . Mechanisms of deubiquitinase specificity and regulation. Annu Rev Biochem. 2017;86:159‐192. 28498721 10.1146/annurev-biochem-061516-044916

[38] Ren J , Yu P , Liu S , et al. Deubiquitylating enzymes in cancer and immunity. Adv Sci (Weinh). 2023;10(36):e2303807. 37888853 10.1002/advs.202303807 PMC10754134

[39] Ilter M , Schulze‐Niemand E , Naumann M , Stein M . Structural dynamics of Lys11‐selective deubiquitinylase cezanne‐1 during the catalytic cycle. J Chem Inf Model. 2023;63(7):2084‐2094. 36943332 10.1021/acs.jcim.2c01281 PMC10091412

[40] Mevissen TET , Kulathu Y , Mulder MPC , et al. Molecular basis of Lys11‐polyubiquitin specificity in the deubiquitinase Cezanne. Nature. 2016;538(7625):402‐405. 27732584 10.1038/nature19836 PMC5295632

[41] Fiil BK , Gyrd‐Hansen M . The Met1‐linked ubiquitin machinery in inflammation and infection. Cell Death Differ. 2021;28(2):557‐569. 33473179 10.1038/s41418-020-00702-x PMC7816137

[42] Licchesi JD , Mieszczanek J , Mevissen TE , et al. An ankyrin‐repeat ubiquitin‐binding domain determines TRABID's specificity for atypical ubiquitin chains. Nat Struct Mol Biol. 2011;19(1):62‐71. 22157957 10.1038/nsmb.2169 PMC5260945

[43] Elliott PR , Leske D , Wagstaff J , et al. Regulation of CYLD activity and specificity by phosphorylation and ubiquitin‐binding CAP‐Gly domains. Cell Rep. 2021;37(1):109777. 34610306 10.1016/j.celrep.2021.109777 PMC8511506

[44] Guan T , Li M , Song Y , et al. Phosphorylation of USP29 by CDK1 governs TWIST1 stability and oncogenic functions. Adv Sci (Weinh). 2023;10(11):e2205873. 36782089 10.1002/advs.202205873 PMC10104637

[45] Huang OW , Ma X , Yin J , et al. Phosphorylation‐dependent activity of the deubiquitinase DUBA. Nat Struct Mol Biol. 2012;19(2):171‐175. 22245969 10.1038/nsmb.2206

[46] Wu L , Zhou Z , Yu Y , et al. Phosphorylation‐dependent deubiquitinase OTUD3 regulates YY1 stability and promotes colorectal cancer progression. Cell Death Dis. 2024;15(2):137. 38351178 10.1038/s41419-024-06526-8 PMC10864350

[47] Zhao Y , Mudge MC , Soll JM , et al. OTUD4 is a phospho‐activated K63 deubiquitinase that regulates MyD88‐dependent signaling. Mol Cell. 2018;69(3):505‐516. 29395066 10.1016/j.molcel.2018.01.009 PMC6819006

[48] Bingol B , Tea JS , Phu L , et al. The mitochondrial deubiquitinase USP30 opposes parkin‐mediated mitophagy. Nature. 2014;510(7505):370‐375. 24896179 10.1038/nature13418

[49] Park J , Shin SC , Jin KS , et al. USP35 dimer prevents its degradation by E3 ligase CHIP through auto‐deubiquitinating activity. Cell Mol Life Sci. 2023;80(4):112. 37004621 10.1007/s00018-023-04740-9 PMC11073304

[50] Chen H , Chen X , Yang L , et al. TRIM54 alleviates inflammation and apoptosis by stabilizing YOD1 in rat tendon‐derived stem cells. J Biol Chem. 2024;300(1):105510. 38042492 10.1016/j.jbc.2023.105510 PMC10801318

[51] Zhang Z , Fang X , Wu X , et al. Acetylation‐dependent deubiquitinase OTUD3 controls MAVS activation in innate antiviral immunity. Mol Cell. 2020;79(2):304‐319. 32679077 10.1016/j.molcel.2020.06.020

[52] Kulathu Y , Garcia FJ , Mevissen TE , et al. Regulation of A20 and other OTU deubiquitinases by reversible oxidation. Nat Commun. 2013;4:1569. 23463012 10.1038/ncomms2567 PMC4176832

[53] Morgan MT , Haj‐Yahya M , Ringel AE , Bandi P , Brik A , Wolberger C . Structural basis for histone H2B deubiquitination by the SAGA DUB module. Science. 2016;351(6274):725‐728. 26912860 10.1126/science.aac5681 PMC4863942

[54] Totsukawa G , Kaneko Y , Uchiyama K , Toh H , Tamura K , Kondo H . VCIP135 deubiquitinase and its binding protein, WAC, in p97ATPase‐mediated membrane fusion. EMBO J. 2011;30(17):3581‐3593. 21811234 10.1038/emboj.2011.260 PMC3181489

[55] Zhang W , Qian W , Gu J , et al. Mutant p53 driven‐LINC00857, a protein scaffold between FOXM1 and deubiquitinase OTUB1, promotes the metastasis of pancreatic cancer. Cancer Lett. 2023;552:215976. 36272615 10.1016/j.canlet.2022.215976

[56] Elliott PR , Nielsen SV , Marco‐Casanova P , et al. Molecular basis and regulation of OTULIN‐LUBAC interaction. Mol Cell. 2014;54(3):335‐348. 24726323 10.1016/j.molcel.2014.03.018 PMC4017264

[57] Wiener R , DiBello AT , Lombardi PM , et al. E2 ubiquitin‐conjugating enzymes regulate the deubiquitinating activity of OTUB1. Nat Struct Mol Biol. 2013;20(9):1033‐1039. 23955022 10.1038/nsmb.2655 PMC3941643

[58] Song H , Zhao C , Yu Z , et al. UAF1 deubiquitinase complexes facilitate NLRP3 inflammasome activation by promoting NLRP3 expression. Nat Commun. 2020;11(1):6042. 33247121 10.1038/s41467-020-19939-8 PMC7695691

[59] Li H , Lim KS , Kim H , et al. Allosteric activation of ubiquitin‐specific proteases by β‐propeller proteins UAF1 and WDR20. Mol Cell. 2016;63(2):249‐260. 27373336 10.1016/j.molcel.2016.05.031 PMC4958508

[60] Ng VH , Spencer Z , Neitzel LR , et al. The USP46 complex deubiquitylates LRP6 to promote Wnt/β‐catenin signaling. Nat Commun. 2023;14(1):6173. 37798301 10.1038/s41467-023-41836-z PMC10556042

[61] Ye Y , Scheel H , Hofmann K , Komander D . Dissection of USP catalytic domains reveals five common insertion points. Mol Biosyst. 2009;5(12):1797‐1808. 19734957 10.1039/b907669g

[62] Liu F , Zhao Y , Pei Y , Lian F , Lin H . Role of the NF‐kB signalling pathway in heterotopic ossification: biological and therapeutic significance. Cell Commun Signal. 2024;22(1):159. 38439078 10.1186/s12964-024-01533-w PMC10910758

[63] Morgan D , Garg M , Tergaonkar V , Tan SY , Sethi G . Pharmacological significance of the non‐canonical NF‐κB pathway in tumorigenesis. Biochim Biophys Acta Rev Cancer. 2020;1874(2):188449. 33058996 10.1016/j.bbcan.2020.188449

[64] Balaji S , Ahmed M , Lorence E , Yan F , Nomie K , Wang M . NF‐κB signaling and its relevance to the treatment of mantle cell lymphoma. J Hematol Oncol. 2018;11(1):83. 29907126 10.1186/s13045-018-0621-5 PMC6002979

[65] Metzig M , Nickles D , Falschlehner C , et al. An RNAi screen identifies USP2 as a factor required for TNF‐α‐induced NF‐κB signaling. Int J Cancer. 2011;129(3):607‐618. 21480224 10.1002/ijc.26124

[66] Mialki RK , Zhao J , Wei J , Mallampalli DF , Zhao Y . Overexpression of USP14 protease reduces I‐κB protein levels and increases cytokine release in lung epithelial cells. J Biol Chem. 2013;288(22):15437‐15441. 23615914 10.1074/jbc.C112.446682 PMC3668705

[67] Liu N , Kong T , Chen X , et al. Ubiquitin‐specific protease 14 regulates LPS‐induced inflammation by increasing ERK1/2 phosphorylation and NF‐κB activation. Mol Cell Biochem. 2017;431(1‐2):87‐96. 28364380 10.1007/s11010-017-2978-0

[68] Yu JS , Huang T , Zhang Y , et al. Substrate‐specific recognition of IKKs mediated by USP16 facilitates autoimmune inflammation. Sci Adv. 2021;7(3):eabc4009. 33523871 10.1126/sciadv.abc4009 PMC7806237

[69] Schaeffer V , Akutsu M , Olma MH , Gomes LC , Kawasaki M , Dikic I . Binding of OTULIN to the PUB domain of HOIP controls NF‐κB signaling. Mol Cell. 2014;54(3):349‐361. 24726327 10.1016/j.molcel.2014.03.016

[70] Xiao N , Li H , Luo J , et al. Ubiquitin‐specific protease 4 (USP4) targets TRAF2 and TRAF6 for deubiquitination and inhibits TNFα‐induced cancer cell migration. Biochem J. 2012;441(3):979‐986. 22029577 10.1042/BJ20111358

[71] Schimmack G , Schorpp K , Kutzner K , et al. YOD1/TRAF6 association balances p62‐dependent IL‐1 signaling to NF‐κB. eLife. 2017;6:e22416. 28244869 10.7554/eLife.22416 PMC5340530

[72] Xie Z , Wu Y , Shen Y , et al. USP7 inhibits osteoclastogenesis via dual effects of attenuating TRAF6/TAK1 axis and stimulating STING signaling. Aging Dis. 2023;14(6):2267‐2283. 37199589 10.14336/AD.2023.0325-1 PMC10676781

[73] Sato Y , Goto E , Shibata Y , et al. Structures of CYLD USP with Met1‐ or Lys63‐linked diubiquitin reveal mechanisms for dual specificity. Nat Struct Mol Biol. 2015;22(3):222‐229. 25686088 10.1038/nsmb.2970

[74] Ea CK , Deng L , Xia ZP , Pineda G , Chen ZJ . Activation of IKK by TNFalpha requires site‐specific ubiquitination of RIP1 and polyubiquitin binding by NEMO. Mol Cell. 2006;22(2):245‐257. 16603398 10.1016/j.molcel.2006.03.026

[75] Lork M , Verhelst K , Beyaert RCYLD . A20 and OTULIN deubiquitinases in NF‐κB signaling and cell death: so similar, yet so different. Cell Death Differ. 2017;24(7):1172‐1183. 28362430 10.1038/cdd.2017.46 PMC5520167

[76] Trompouki E , Hatzivassiliou E , Tsichritzis T , Farmer H , Ashworth A , Mosialos G . CYLD is a deubiquitinating enzyme that negatively regulates NF‐kappaB activation by TNFR family members. Nature. 2003;424(6950):793‐796. 12917689 10.1038/nature01803

[77] Wang Z , Zhang Y , Shen Y , et al. Unlocking hepatocellular carcinoma aggression: sTAMBPL1‐mediated TRAF2 deubiquitination activates WNT/PI3K/NF‐kb signaling pathway. Biol Direct. 2024;19(1):18. 38419066 10.1186/s13062-024-00460-7 PMC10903047

[78] Spel L , Nieuwenhuis J , Haarsma R , et al. Nedd4‐binding protein 1 and TNFAIP3‐interacting protein 1 control MHC‐1 display in neuroblastoma. Cancer Res. 2018;78(23):6621‐6631. 30213788 10.1158/0008-5472.CAN-18-0545

[79] Hu H , Brittain GC , Chang JH , et al. OTUD7B controls non‐canonical NF‐κB activation through deubiquitination of TRAF3. Nature. 2013;494(7437):371‐374. 23334419 10.1038/nature11831 PMC3578967

[80] Zhou Q , Wang H , Schwartz DM , et al. Loss‐of‐function mutations in TNFAIP3 leading to A20 haploinsufficiency cause an early‐onset autoinflammatory disease. Nat Genet. 2016;48(1):67‐73. 26642243 10.1038/ng.3459 PMC4777523

[81] Heyninck K , De Valck D , Vanden Berghe W , et al. The zinc finger protein A20 inhibits TNF‐induced NF‐kappaB‐dependent gene expression by interfering with an RIP‐ or TRAF2‐mediated transactivation signal and directly binds to a novel NF‐kappaB‐inhibiting protein ABIN. J Cell Biol. 1999;145(7):1471‐1482. 10385526 10.1083/jcb.145.7.1471 PMC2133159

[82] Park HB , Baek KH . E3 ligases and deubiquitinating enzymes regulating the MAPK signaling pathway in cancers. Biochim Biophys Acta Rev Cancera. 2022;1877(3):188736. 10.1016/j.bbcan.2022.188736 35589008

[83] Li W , Cui K , Prochownik EV , Li Y . The deubiquitinase USP21 stabilizes MEK2 to promote tumor growth. Cell Death Dis. 2018;9(5):482. 29706623 10.1038/s41419-018-0523-z PMC5924753

[84] Zhang H , Han Y , Xiao W , et al. USP4 promotes the proliferation, migration, and invasion of esophageal squamous cell carcinoma by targeting TAK1. Cell Death Dis. 2023;14(11):730. 37949874 10.1038/s41419-023-06259-0 PMC10638297

[85] Kumari N , Jaynes PW , Saei A , Iyengar PV , Richard JLC , Eichhorn PJA . The roles of ubiquitin modifying enzymes in neoplastic disease. Biochim Biophys Acta Rev Cancera. 2017;1868(2):456‐483. 10.1016/j.bbcan.2017.09.002 28923280

[86] Lei S , He Z , Chen T , et al. Long noncoding RNA 00976 promotes pancreatic cancer progression through OTUD7B by sponging miR‐137 involving EGFR/MAPK pathway. J Exp Clin Cancer Res. 2019;38(1):470. 31747939 10.1186/s13046-019-1388-4 PMC6868788

[87] Chen Y , Qiang Y , Fan J , et al. Aggresome formation promotes ASK1/JNK signaling activation and stemness maintenance in ovarian cancer. Nat Commun. 2024;15(1):1321. 38351029 10.1038/s41467-024-45698-x PMC10864366

[88] Lou B , Ma G , Yu X , et al. Deubiquitinase OTUD5 promotes hepatitis B virus replication by removing K48‐linked ubiquitination of HBV core/precore and upregulates HNF4ɑ expressions by inhibiting the ERK1/2/mitogen‐activated protein kinase pathway. Cell Mol Life Sci. 2023;80(11):336. 37897511 10.1007/s00018-023-04995-2 PMC10613150

[89] Xuan NT , Trung DM , Minh NN , et al. Regulation of p38MAPK‐mediated dendritic cell functions by the deubiquitylase otubain 1. Hla. 2019;93(6):462‐470. 30908891 10.1111/tan.13534

[90] Sun H , Ou B , Zhao S , et al. USP11 promotes growth and metastasis of colorectal cancer via PPP1CA‐mediated activation of ERK/MAPK signaling pathway. EBioMedicine. 2019;48:236‐247. 31521612 10.1016/j.ebiom.2019.08.061 PMC6838424

[91] Yu L , Wei J , Liu P . Attacking the PI3K/Akt/mTOR signaling pathway for targeted therapeutic treatment in human cancer. Semin Cancer Biol. 2022;85:69‐94. 34175443 10.1016/j.semcancer.2021.06.019

[92] Alzahrani AS . PI3K/Akt/mTOR inhibitors in cancer: at the bench and bedside. Semin Cancer Biol. 2019;59:125‐132. 31323288 10.1016/j.semcancer.2019.07.009

[93] Sun H , Meng Y , Yao L , et al. Ubiquitin‐specific protease 22 controls melanoma metastasis and vulnerability to ferroptosis through targeting SIRT1/PTEN/PI3K signaling. MedComm. 2024;5(8):e684. 39135915 10.1002/mco2.684 PMC11318338

[94] Zhang J , Zhang P , Wei Y , et al. Deubiquitylation and stabilization of PTEN by USP13. Nat Cell Biol. 2013;15(12):1486‐1494. 24270891 10.1038/ncb2874 PMC3951854

[95] Sacco JJ , Yau TY , Darling S , et al. The deubiquitylase Ataxin‐3 restricts PTEN transcription in lung cancer cells. Oncogene. 2014;33(33):4265‐4272. 24292675 10.1038/onc.2013.512 PMC4351423

[96] Yang WL , Jin G , Li CF , et al. Cycles of ubiquitination and deubiquitination critically regulate growth factor‐mediated activation of Akt signaling. Sci Signal. 2013;6(257):ra3. 23300340 10.1126/scisignal.2003197 PMC3862898

[97] Fan G , Wang F , Chen Y , et al. The deubiquitinase OTUD1 noncanonically suppresses Akt activation through its N‐terminal intrinsically disordered region. Cell Rep. 2023;42(1):111916. 36640312 10.1016/j.celrep.2022.111916

[98] Zhang J , Zha Y , Jiao Y , Li Y , Zhang S . Protective role of cezanne in doxorubicin‐induced cardiotoxicity by inhibiting autophagy, apoptosis and oxidative stress. Toxicology. 2023;485:153426. 36639017 10.1016/j.tox.2023.153426

[99] Goldbraikh D , Neufeld D , Eid‐Mutlak Y , et al. USP1 deubiquitinates Akt to inhibit PI3K‐Akt‐FoxO signaling in muscle during prolonged starvation. EMBO Rep. 2020;21(4):e48791. 32133736 10.15252/embr.201948791 PMC7132338

[100] Cho JH , Kim K , Kim SA , et al. Deubiquitinase OTUD5 is a positive regulator of mTORC1 and mTORC2 signaling pathways. Cell Death Differ. 2021;28(3):900‐914. 33110214 10.1038/s41418-020-00649-z PMC7937674

[101] Hertel A , Alves LM , Dutz H , et al. USP32‐regulated LAMTOR1 ubiquitination impacts mTORC1 activation and autophagy induction. Cell Rep. 2022;41(10):111653. 36476874 10.1016/j.celrep.2022.111653

[102] Zhang Y , Wang X . Targeting the Wnt/β‐catenin signaling pathway in cancer. J Hematol Oncol. 2020;13(1):165. 33276800 10.1186/s13045-020-00990-3 PMC7716495

[103] Nusse R , Clevers H . Wnt/β‐catenin signaling, disease, and emerging therapeutic modalities. Cell. 2017;169(6):985‐999. 28575679 10.1016/j.cell.2017.05.016

[104] Rim EY , Clevers H , Nusse R . The Wnt pathway: from signaling mechanisms to synthetic modulators. Annu Rev Biochem. 2022;91:571‐598. 35303793 10.1146/annurev-biochem-040320-103615

[105] Ma X , Qi W , Pan H , Yang F , Deng J . Overexpression of USP5 contributes to tumorigenesis in non‐small cell lung cancer via the stabilization of β‐catenin protein. Am J Cancer Res. 2018;8(11):2284‐2295. 30555744 PMC6291653

[106] Yang B , Zhang S , Wang Z , et al. Deubiquitinase USP9X deubiquitinates β‐catenin and promotes high grade glioma cell growth. Oncotarget. 2016;7(48):79515‐79525. 27783990 10.18632/oncotarget.12819 PMC5346732

[107] MacDonald BT , Tamai K , He X . Wnt/beta‐catenin signaling: components, mechanisms, and diseases. Dev Cell. 2009;17(1):9‐26. 19619488 10.1016/j.devcel.2009.06.016 PMC2861485

[108] Chou CK , Chang YT , Korinek M , et al. The regulations of deubiquitinase USP15 and its pathophysiological mechanisms in diseases. Int J Mol Sci. 2017;18(3):483. 28245560 10.3390/ijms18030483 PMC5372499

[109] Wang W , Li M , Ponnusamy S , et al. ABL1‐dependent OTULIN phosphorylation promotes genotoxic Wnt/β‐catenin activation to enhance drug resistance in breast cancers. Nat Commun. 2020;11(1):3965. 32770022 10.1038/s41467-020-17770-9 PMC7414915

[110] Lee Y , Piao HL , Kim J . OTUD7B activates Wnt signaling pathway through the interaction with LEF1. Biomolecules. 2023;13(6). 10.3390/biom13061001 PMC10295924 37371581

[111] Nakamura BN , Glazier A , Kattah MG , et al. A20 regulates canonical wnt‐signaling through an interaction with RIPK4. PLoS One. 2018;13(5):e0195893. 29718933 10.1371/journal.pone.0195893 PMC5931457

[112] Li FL , Guan KL . The two sides of Hippo pathway in cancer. Semin Cancer Biol. 2022;85:33‐42. 34265423 10.1016/j.semcancer.2021.07.006

[113] Yan C , Yang H , Su P , et al. OTUB1 suppresses Hippo signaling via modulating YAP protein in gastric cancer. Oncogene. 2022;41(48):5186‐5198. 36271031 10.1038/s41388-022-02507-3 PMC9700521

[114] Zhang Z , Du J , Wang S , et al. OTUB2 promotes cancer metastasis via hippo‐independent activation of YAP and TAZ. Mol Cell. 2019;73(1):7‐21. 30472188 10.1016/j.molcel.2018.10.030

[115] Liu D , Li Q , Zang Y , et al. USP1 modulates hepatocellular carcinoma progression via the Hippo/TAZ axis. Cell Death Dis. 2023;14(4):264. 37041150 10.1038/s41419-023-05777-1 PMC10090121

[116] Tian Z , Xu C , He W , et al. The deubiquitinating enzyme USP19 facilitates hepatocellular carcinoma progression through stabilizing YAP. Cancer Lett. 2023;577:216439. 37832781 10.1016/j.canlet.2023.216439

[117] Kim Y , Kim W , Song Y , et al. Deubiquitinase YOD1 potentiates YAP/TAZ activities through enhancing ITCH stability. Proc Nat Acad Sci USA. 2017;114(18):4691‐4696. 28416659 10.1073/pnas.1620306114 PMC5422760

[118] Zhu C , Ji X , Zhang H , et al. Deubiquitylase USP9X suppresses tumorigenesis by stabilizing large tumor suppressor kinase 2 (LATS2) in the Hippo pathway. J Biol Chem. 2018;293(4):1178‐1191. 29183995 10.1074/jbc.RA117.000392 PMC5787797

[119] Li L , Liu T , Li Y , et al. The deubiquitinase USP9X promotes tumor cell survival and confers chemoresistance through YAP1 stabilization. Oncogene. 2018;37(18):2422‐2431. 29449692 10.1038/s41388-018-0134-2 PMC5940338

[120] Wang D , Zhang Y , Xu X , et al. YAP promotes the activation of NLRP3 inflammasome via blocking K27‐linked polyubiquitination of NLRP3. Nat Commun. 2021;12(1):2674. 33976226 10.1038/s41467-021-22987-3 PMC8113592

[121] Peng D , Fu M , Wang M , Wei Y , Wei X . Targeting TGF‐β signal transduction for fibrosis and cancer therapy. Mol Cancer. 2022;21(1):104. 35461253 10.1186/s12943-022-01569-x PMC9033932

[122] Shi X , Yang J , Deng S , et al. TGF‐β signaling in the tumor metabolic microenvironment and targeted therapies. J Hematol Oncol. 2022;15(1):135. 36115986 10.1186/s13045-022-01349-6 PMC9482317

[123] Liu S , González‐Prieto R , Zhang M , et al. Deubiquitinase activity profiling identifies UCHL1 as a candidate oncoprotein that promotes TGFβ‐induced breast cancer metastasis. Clin Cancer Res. 2020;26(6):1460‐1473. 31857432 10.1158/1078-0432.CCR-19-1373 PMC7611208

[124] Eichhorn PJ , Rodón L , Gonzàlez‐Juncà A , et al. USP15 stabilizes TGF‐β receptor I and promotes oncogenesis through the activation of TGF‐β signaling in glioblastoma. Nat Med. 2012;18(3):429‐435. 22344298 10.1038/nm.2619

[125] Huang Z , Shen S , Wang M , et al. Mouse endothelial OTUD1 promotes angiotensin II‐induced vascular remodeling by deubiquitinating SMAD3. EMBO Rep. 2023;24(3):e56135. 36579465 10.15252/embr.202256135 PMC9986815

[126] Zhang Z , Fan Y , Xie F , et al. Breast cancer metastasis suppressor OTUD1 deubiquitinates SMAD7. Nat Commun. 2017;8(1):2116. 29235476 10.1038/s41467-017-02029-7 PMC5727433

[127] Kit Leng Lui S, Iyengar PV , Jaynes P , et al. USP26 regulates TGF‐β signaling by deubiquitinating and stabilizing SMAD7. EMBO Rep. 2017;18(5):797‐808. 28381482 10.15252/embr.201643270 PMC5412796

[128] Wu Y , Yu X , Yi X , et al. Aberrant phosphorylation of SMAD4 Thr277‐mediated USP9x‐SMAD4 interaction by free fatty acids promotes breast cancer metastasis. Cancer Res. 2017;77(6):1383‐1394. 28115363 10.1158/0008-5472.CAN-16-2012 PMC5354968

[129] Dupont S , Mamidi A , Cordenonsi M , et al. FAM/USP9x, a deubiquitinating enzyme essential for TGFbeta signaling, controls Smad4 monoubiquitination. Cell. 2009;136(1):123‐135. 19135894 10.1016/j.cell.2008.10.051

[130] Bian S , Ni W , Zhou L , et al. Ubiquitin‐specific protease 1 facilitates hepatocellular carcinoma progression by modulating mitochondrial fission and metabolic reprogramming via cyclin‐dependent kinase 5 stabilization. Cell Death Differ. 2024;31(9):1202‐1218. 39009653 10.1038/s41418-024-01342-1 PMC11369097

[131] Raimondi M , Cesselli D , Di Loreto C , La Marra F , Schneider C , Demarchi F . USP1 (ubiquitin specific peptidase 1) targets ULK1 and regulates its cellular compartmentalization and autophagy. Autophagy. 2019;15(4):613‐630. 30335599 10.1080/15548627.2018.1535291 PMC6526860

[132] Ma L , Lin K , Chang G , et al. Aberrant activation of β‐catenin signaling drives glioma tumorigenesis via USP1‐mediated stabilization of EZH2. Cancer Res. 2019;79(1):72‐85. 30425057 10.1158/0008-5472.CAN-18-1304 PMC9646285

[133] Guervilly JH , Renaud E , Takata M , Rosselli F . USP1 deubiquitinase maintains phosphorylated CHK1 by limiting its DDB1‐dependent degradation. Hum Mol Genet. 2011;20(11):2171‐2181. 21389083 10.1093/hmg/ddr103 PMC3090195

[134] Nijman SM , Huang TT , Dirac AM , et al. The deubiquitinating enzyme USP1 regulates the Fanconi anemia pathway. Mol Cell. 2005;17(3):331‐339. 15694335 10.1016/j.molcel.2005.01.008

[135] Huang TT , Nijman SM , Mirchandani KD , et al. Regulation of monoubiquitinated PCNA by DUB autocleavage. Nat Cell Biol. 2006;8(4):339‐347. 16531995 10.1038/ncb1378

[136] Zhao Y , Wang X , Wang Q , et al. USP2a supports metastasis by tuning TGF‐β signaling. Cell Rep. 2018;22(9):2442‐2454. 29490279 10.1016/j.celrep.2018.02.007

[137] Kuang Z , Liu X , Zhang N , et al. USP2 promotes tumor immune evasion via deubiquitination and stabilization of PD‐L1. Cell Death Differ. 2023;30(10):2249‐2264. 37670038 10.1038/s41418-023-01219-9 PMC10589324

[138] Benassi B , Flavin R , Marchionni L , et al. MYC is activated by USP2a‐mediated modulation of microRNAs in prostate cancer. Cancer Discov. 2012;2(3):236‐247. 22585994 10.1158/2159-8290.CD-11-0219 PMC3523361

[139] Li T , Yan B , Ma Y , et al. Ubiquitin‐specific protease 4 promotes hepatocellular carcinoma progression via cyclophilin A stabilization and deubiquitination. Cell Death Dis. 2018;9(2):148. 29396555 10.1038/s41419-017-0182-5 PMC5833721

[140] Qiu C , Liu Y , Mei Y , et al. Correction for: ubiquitin‐specific protease 4 promotes metastasis of hepatocellular carcinoma by increasing TGF‐β signaling‐induced epithelial‐mesenchymal transition. Aging. 2019;11(10):3408‐3409. 31164493 10.18632/aging.102010 PMC6555464

[141] Yun SI , Kim HH , Yoon JH , et al. Ubiquitin specific protease 4 positively regulates the WNT/β‐catenin signaling in colorectal cancer. Mol Oncol. 2015;9(9):1834‐1851. 26189775 10.1016/j.molonc.2015.06.006 PMC5528720

[142] Wang S , Juan J , Zhang Z , et al. Inhibition of the deubiquitinase USP5 leads to c‐Maf protein degradation and myeloma cell apoptosis. Cell Death Dis. 2017;8(9):e3058. 28933784 10.1038/cddis.2017.450 PMC5636991

[143] Xue S , Wu W , Wang Z , et al. USP5 promotes metastasis in non‐small cell lung cancer by inducing epithelial‐mesenchymal transition via Wnt/β‐Catenin pathway. Front Pharmacol. 2020;11:668. 32477134 10.3389/fphar.2020.00668 PMC7236764

[144] Xu X , Huang A , Cui X , et al. Ubiquitin specific peptidase 5 regulates colorectal cancer cell growth by stabilizing Tu translation elongation factor. Theranostics. 2019;9(14):4208‐4220. 31281542 10.7150/thno.33803 PMC6592179

[145] Yan B , Guo J , Wang Z , et al. The ubiquitin‐specific protease 5 mediated deubiquitination of LSH links metabolic regulation of ferroptosis to hepatocellular carcinoma progression. MedComm. 2023;4(4):e337. 37492786 10.1002/mco2.337 PMC10363799

[146] Li J , Wang Y , Luo Y , et al. USP5‐Beclin 1 axis overrides p53‐dependent senescence and drives Kras‐induced tumorigenicity. Nat Commun. 2022;13(1):7799. 36528652 10.1038/s41467-022-35557-y PMC9759531

[147] Pan J , Qiao Y , Chen C , et al. USP5 facilitates non‐small cell lung cancer progression through stabilization of PD‐L1. Cell Death Dis. 2021;12(11):1051. 34741014 10.1038/s41419-021-04356-6 PMC8571306

[148] Huang W , Liu X , Zhang Y , et al. USP5 promotes breast cancer cell proliferation and metastasis by stabilizing HIF2α. J Cell Physiol. 2022;237(4):2211‐2219. 35102545 10.1002/jcp.30686

[149] He Y , Jiang S , Zhong Y , et al. USP7 promotes non‐small‐cell lung cancer cell glycolysis and survival by stabilizing and activating c‐Abl. Clin Transl Med. 2023;13(12):e1509. 38082439 10.1002/ctm2.1509 PMC10713873

[150] Cai JB , Shi GM , Dong ZR , et al. Ubiquitin‐specific protease 7 accelerates p14(ARF) degradation by deubiquitinating thyroid hormone receptor‐interacting protein 12 and promotes hepatocellular carcinoma progression. Hepatology (Baltimore, Md). 2015;61(5):1603‐1614. 10.1002/hep.27682 25557975

[151] Shao ZY , Yang WD , Qiu H , et al. The role of USP7‐YY1 interaction in promoting colorectal cancer growth and metastasis. Cell Death Dis. 2024;15(5):347. 38769122 10.1038/s41419-024-06740-4 PMC11106261

[152] Qi SM , Cheng G , Cheng XD , et al. Targeting USP7‐mediated deubiquitination of MDM2/MDMX‐p53 pathway for cancer therapy: are we there yet?. Front Cell Dev Biol. 2020;8:233. 32300595 10.3389/fcell.2020.00233 PMC7142254

[153] Wang Z , Kang W , Li O , et al. Abrogation of USP7 is an alternative strategy to downregulate PD‐L1 and sensitize gastric cancer cells to T cells killing. Acta Pharm Sin B. 2021;11(3):694‐707. 33777676 10.1016/j.apsb.2020.11.005 PMC7982505

[154] Dai X , Lu L , Deng S , et al. USP7 targeting modulates anti‐tumor immune response by reprogramming tumor‐associated macrophages in lung cancer. Theranostics. 2020;10(20):9332‐9347. 32802195 10.7150/thno.47137 PMC7415808

[155] Haq S , Sarodaya N , Karapurkar JK , et al. CYLD destabilizes NoxO1 protein by promoting ubiquitination and regulates prostate cancer progression. Cancer Lett. 2022;525:146‐157. 34742871 10.1016/j.canlet.2021.10.032

[156] Tauriello DV , Haegebarth A , Kuper I , et al. Loss of the tumor suppressor CYLD enhances Wnt/beta‐catenin signaling through K63‐linked ubiquitination of Dvl. Mol Cell. 2010;37(5):607‐619. 20227366 10.1016/j.molcel.2010.01.035

[157] Fernández‐Majada V , Welz PS , Ermolaeva MA , et al. The tumour suppressor CYLD regulates the p53 DNA damage response. Nat Commun. 2016;7:12508. 27561390 10.1038/ncomms12508 PMC5007442

[158] Miliani de Marval P , Lutfeali S , Jin JY , Leshin B , Selim MA , Zhang JY . CYLD inhibits tumorigenesis and metastasis by blocking JNK/AP1 signaling at multiple levels. Cancer Prev Res (Phila). 2011;4(6):851‐859. 21478324 10.1158/1940-6207.CAPR-10-0360 PMC3107906

[159] Sun Q , Zhang J , Li X , et al. The ubiquitin‐specific protease 8 antagonizes melatonin‐induced endocytic degradation of MT(1) receptor to promote lung adenocarcinoma growth. J Adv Res. 2022;41:1‐12. 36328739 10.1016/j.jare.2022.01.015 PMC9637587

[160] Tang J , Long G , Hu K , et al. Targeting USP8 inhibits O‐GlcNAcylation of SLC7A11 to promote ferroptosis of hepatocellular carcinoma via stabilization of OGT. Adv Sci (Weinh). 2023;10(33):e2302953. 37867237 10.1002/advs.202302953 PMC10667802

[161] Peng H , Yang F , Hu Q , et al. The ubiquitin‐specific protease USP8 directly deubiquitinates SQSTM1/p62 to suppress its autophagic activity. Autophagy. 2020;16(4):698‐708. 31241013 10.1080/15548627.2019.1635381 PMC7138243

[162] Potu H , Peterson LF , Kandarpa M , et al. Usp9x regulates Ets‐1 ubiquitination and stability to control NRAS expression and tumorigenicity in melanoma. Nat Commun. 2017;8:14449. 28198367 10.1038/ncomms14449 PMC5316860

[163] Schwickart M , Huang X , Lill JR , et al. Deubiquitinase USP9X stabilizes MCL1 and promotes tumour cell survival. Nature. 2010;463(7277):103‐107. 20023629 10.1038/nature08646

[164] Engel K , Rudelius M , Slawska J , et al. USP9X stabilizes XIAP to regulate mitotic cell death and chemoresistance in aggressive B‐cell lymphoma. EMBO Mol Med. 2016;8(8):851‐862. 27317434 10.15252/emmm.201506047 PMC4967940

[165] Khan OM , Carvalho J , Spencer‐Dene B , et al. The deubiquitinase USP9X regulates FBW7 stability and suppresses colorectal cancer. J Clin Invest. 2018;128(4):1326‐1337. 29346117 10.1172/JCI97325 PMC5873885

[166] McGarry E , Gaboriau D , Rainey MD , Restuccia U , Bachi A , Santocanale C . The deubiquitinase USP9X maintains DNA replication fork stability and DNA damage checkpoint responses by regulating CLASPIN during S‐Phase. Cancer Res. 2016;76(8):2384‐2393. 26921344 10.1158/0008-5472.CAN-15-2890

[167] Wang X , Xia S , Li H , et al. The deubiquitinase USP10 regulates KLF4 stability and suppresses lung tumorigenesis. Cell Death Differ. 2020;27(6):1747‐1764. 31748695 10.1038/s41418-019-0458-7 PMC7244734

[168] Dwane L , O'Connor AE , Das S , et al. A functional genomic screen identifies the deubiquitinase USP11 as a novel transcriptional regulator of ERα in breast cancer. Cancer Res. 2020;80(22):5076‐5088. 33004351 10.1158/0008-5472.CAN-20-0214

[169] Qiao L , Hu W , Li L , Chen X , Liu L , Wang J . USP11 promotes glycolysis by regulating HIF‐1α stability in hepatocellular carcinoma. J Cell Mol Med. 2024;28(2):e18017. 38229475 10.1111/jcmm.18017 PMC10826445

[170] Ting X , Xia L , Yang J , et al. USP11 acts as a histone deubiquitinase functioning in chromatin reorganization during DNA repair. Nucleic Acids Res. 2019;47(18):9721‐9740. 31504778 10.1093/nar/gkz726 PMC6765148

[171] Stucki M , Clapperton JA , Mohammad D , Yaffe MB , Smerdon SJ , Jackson SP . MDC1 directly binds phosphorylated histone H2AX to regulate cellular responses to DNA double‐strand breaks. Cell. 2005;123(7):1213‐1226. 16377563 10.1016/j.cell.2005.09.038

[172] Shah P , Qiang L , Yang S , Soltani K , He YY . Regulation of XPC deubiquitination by USP11 in repair of UV‐induced DNA damage. Oncotarget. 2017;8(57):96522‐96535. 29228550 10.18632/oncotarget.22105 PMC5722502

[173] Li H , Roy M , Liang L , et al. Deubiquitylase USP12 induces pro‐survival autophagy and bortezomib resistance in multiple myeloma by stabilizing HMGB1. Oncogene. 2022;41(9):1298‐1308. 34997217 10.1038/s41388-021-02167-9

[174] Burska UL , Harle VJ , Coffey K , et al. Deubiquitinating enzyme Usp12 is a novel co‐activator of the androgen receptor. J Biol Chem. 2013;288(45):32641‐32650. 24056413 10.1074/jbc.M113.485912 PMC3820899

[175] McClurg UL , Chit N , Azizyan M , et al. Molecular mechanism of the TP53‐MDM2‐AR‐AKT signalling network regulation by USP12. Oncogene. 2018;37(34):4679‐4691. 29755129 10.1038/s41388-018-0283-3

[176] Fang X , Zhou W , Wu Q , et al. Deubiquitinase USP13 maintains glioblastoma stem cells by antagonizing FBXL14‐mediated Myc ubiquitination. J Exp Med. 2017;214(1):245‐267. 27923907 10.1084/jem.20151673 PMC5206492

[177] Zhang S , Zhang M , Jing Y , et al. Deubiquitinase USP13 dictates MCL1 stability and sensitivity to BH3 mimetic inhibitors. Nat Commun. 2018;9(1):215. 29335437 10.1038/s41467-017-02693-9 PMC5768685

[178] Li Y , Luo K , Yin Y , et al. USP13 regulates the RAP80‐BRCA1 complex dependent DNA damage response. Nat Commun. 2017;8:15752. 28569838 10.1038/ncomms15752 PMC5461494

[179] Han C , Yang L , Choi HH , et al. Amplification of USP13 drives ovarian cancer metabolism. Nat Commun. 2016;7:13525. 27892457 10.1038/ncomms13525 PMC5133706

[180] Jung H , Kim BG , Han WH , et al. Deubiquitination of Dishevelled by Usp14 is required for Wnt signaling. Oncogenesis. 2013;2(8):e64. 23958854 10.1038/oncsis.2013.28 PMC3759127

[181] Zhang B , Li M , Huang P , Guan XY , Zhu YH . Overexpression of ubiquitin specific peptidase 14 predicts unfavorable prognosis in esophageal squamous cell carcinoma. Thorac Cancer. 2017;8(4):344‐349. 28509417 10.1111/1759-7714.12453 PMC5494452

[182] Song C , Ma R , Yang X , Pang S . The deubiquitinating enzyme USP14 regulates leukemic chemotherapy drugs‐induced cell apoptosis by suppressing ubiquitination of aurora kinase B. Cell Physiol Biochem. 2017;42(3):965‐973. 28662510 10.1159/000478679

[183] Liao Y , Xia X , Liu N , et al. Growth arrest and apoptosis induction in androgen receptor‐positive human breast cancer cells by inhibition of USP14‐mediated androgen receptor deubiquitination. Oncogene. 2018;37(14):1896‐1910. 29353883 10.1038/s41388-017-0069-z PMC5886989

[184] Zhang X , Geng L , Tang Y , et al. Ubiquitin‐specific protease 14 targets PFKL‐mediated glycolysis to promote the proliferation and migration of oral squamous cell carcinoma. J Transl Med. 2024;22(1):193. 38388430 10.1186/s12967-024-04943-z PMC10885370

[185] Zhou L , Jiang H , Du J , et al. USP15 inhibits multiple myeloma cell apoptosis through activating a feedback loop with the transcription factor NF‐κBp65. Exp Mol Med. 2018;50(11):1‐12. 10.1038/s12276-018-0180-4 PMC6244212 30459344

[186] Zou Q , Jin J , Hu H , et al. USP15 stabilizes MDM2 to mediate cancer‐cell survival and inhibit antitumor T cell responses. Nat Immunol. 2014;15(6):562‐570. 24777531 10.1038/ni.2885 PMC4032322

[187] Peng Y , Liao Q , Tan W , et al. The deubiquitylating enzyme USP15 regulates homologous recombination repair and cancer cell response to PARP inhibitors. Nat Commun. 2019;10(1):1224. 30874560 10.1038/s41467-019-09232-8 PMC6420636

[188] Fielding AB , Concannon M , Darling S , et al. The deubiquitylase USP15 regulates topoisomerase II alpha to maintain genome integrity. Oncogene. 2018;37(17):2326‐2342. 29429988 10.1038/s41388-017-0092-0 PMC5916918

[189] Villeneuve NF , Tian W , Wu T , et al. USP15 negatively regulates Nrf2 through deubiquitination of Keap1. Mol Cell. 2013;51(1):68‐79. 23727018 10.1016/j.molcel.2013.04.022 PMC3732832

[190] Liu WT , Huang KY , Lu MC , et al. TGF‐β upregulates the translation of USP15 via the PI3K/AKT pathway to promote p53 stability. Oncogene. 2017;36(19):2715‐2723. 27893708 10.1038/onc.2016.424 PMC5442427

[191] Fukushima T , Yoshihara H , Furuta H , et al. USP15 attenuates IGF‐I signaling by antagonizing Nedd4‐induced IRS‐2 ubiquitination. Biochem Biophys Res Commun. 2017;484(3):522‐528. 28126338 10.1016/j.bbrc.2017.01.101

[192] Pereg Y , Liu BY , O'Rourke KM , et al. Ubiquitin hydrolase Dub3 promotes oncogenic transformation by stabilizing Cdc25A. Nat Cell Biol. 2010;12(4):400‐406. 20228808 10.1038/ncb2041

[193] Hernández‐Pérez S , Cabrera E , Salido E , et al. DUB3 and USP7 de‐ubiquitinating enzymes control replication inhibitor Geminin: molecular characterization and associations with breast cancer. Oncogene. 2017;36(33):4802‐4809. 28288134 10.1038/onc.2017.21

[194] Lin Y , Wang Y , Shi Q , et al. Stabilization of the transcription factors slug and twist by the deubiquitinase dub3 is a key requirement for tumor metastasis. Oncotarget. 2017;8(43):75127‐75140. 29088851 10.18632/oncotarget.20561 PMC5650406

[195] Wu Y , Wang Y , Lin Y , et al. Dub3 inhibition suppresses breast cancer invasion and metastasis by promoting Snail1 degradation. Nat Commun. 2017;8:14228. 28198361 10.1038/ncomms14228 PMC5316870

[196] Mehić M , de Sa VK , Hebestreit S , Heldin CH , Heldin P . The deubiquitinating enzymes USP4 and USP17 target hyaluronan synthase 2 and differentially affect its function. Oncogenesis. 2017;6(6):e348. 28604766 10.1038/oncsis.2017.45 PMC5519194

[197] Song C , Liu W , Li J . USP17 is upregulated in osteosarcoma and promotes cell proliferation, metastasis, and epithelial‐mesenchymal transition through stabilizing SMAD4. Tumour Biol. 2017;39(7):1010428317717138. 28670958 10.1177/1010428317717138

[198] Jin X , Yan Y , Wang D , et al. DUB3 promotes BET inhibitor resistance and cancer progression by deubiquitinating BRD4. Mol Cell. 2018;71(4):592‐605. 30057199 10.1016/j.molcel.2018.06.036 PMC6086352

[199] Song C , Peng J , Wei Y , et al. USP18 promotes tumor metastasis in esophageal squamous cell carcinomas via deubiquitinating ZEB1. Exp Cell Res. 2021;409(1):112884. 34743935 10.1016/j.yexcr.2021.112884

[200] Guo Y , Dolinko AV , Chinyengetere F , et al. Blockade of the ubiquitin protease UBP43 destabilizes transcription factor PML/RARα and inhibits the growth of acute promyelocytic leukemia. Cancer Res. 2010;70(23):9875‐9885. 20935222 10.1158/0008-5472.CAN-10-1100 PMC2999664

[201] Cai J , Liu T , Jiang X , Guo C , Liu A , Xiao X . Downregulation of USP18 inhibits growth and induces apoptosis in hepatitis B virus‐related hepatocellular carcinoma cells by suppressing BCL2L1. Exp Cell Res. 2017;358(2):315‐322. 28709980 10.1016/j.yexcr.2017.07.006

[202] Mustachio LM , Lu Y , Tafe LJ , et al. Deubiquitinase USP18 loss mislocalizes and destabilizes KRAS in lung cancer. Mol Cancer Res. 2017;15(7):905‐914. 28242811 10.1158/1541-7786.MCR-16-0369 PMC5635999

[203] Wu C , Luo K , Zhao F , et al. USP20 positively regulates tumorigenesis and chemoresistance through β‐catenin stabilization. Cell Death Differ. 2018;25(10):1855‐1869. 29867130 10.1038/s41418-018-0138-z PMC6180113

[204] Li W , Shen M , Jiang YZ , et al. Deubiquitinase USP20 promotes breast cancer metastasis by stabilizing SNAI2. Genes Dev. 2020;34(19‐20):1310‐1315. 32943575 10.1101/gad.339804.120 PMC7528704

[205] Wang C , Yang C , Ji J , et al. Deubiquitinating enzyme USP20 is a positive regulator of Claspin and suppresses the malignant characteristics of gastric cancer cells. Int J Oncol. 2017;50(4):1136‐1146. 28350092 10.3892/ijo.2017.3904 PMC5363881

[206] Lin Z , Tan C , Qiu Q , et al. Ubiquitin‐specific protease 22 is a deubiquitinase of CCNB1. Cell Discov. 2015;1:15028. 27030811 10.1038/celldisc.2015.28 PMC4809424

[207] Gennaro VJ , Stanek TJ , Peck AR , et al. Control of CCND1 ubiquitylation by the catalytic SAGA subunit USP22 is essential for cell cycle progression through G1 in cancer cells. Proc Nat Acad Sci USA. 2018;115(40):E9298‐e9307. 30224477 10.1073/pnas.1807704115 PMC6176615

[208] Kim D , Hong A , Park HI , et al. Deubiquitinating enzyme USP22 positively regulates c‐Myc stability and tumorigenic activity in mammalian and breast cancer cells. J Cell Physiol. 2017;232(12):3664‐3676. 28160502 10.1002/jcp.25841

[209] Atanassov BS , Dent SY . USP22 regulates cell proliferation by deubiquitinating the transcriptional regulator FBP1. EMBO Rep. 2011;12(9):924‐930. 21779003 10.1038/embor.2011.140 PMC3166460

[210] Li C , Irrazabal T , So CC , et al. The H2B deubiquitinase Usp22 promotes antibody class switch recombination by facilitating non‐homologous end joining. Nat Commun. 2018;9(1):1006. 29520062 10.1038/s41467-018-03455-x PMC5843634

[211] Zhang H , Han B , Lu H , et al. USP22 promotes resistance to EGFR‐TKIs by preventing ubiquitination‐mediated EGFR degradation in EGFR‐mutant lung adenocarcinoma. Cancer Lett. 2018;433:186‐198. 29981430 10.1016/j.canlet.2018.07.002

[212] Zhou A , Lin K , Zhang S , et al. Nuclear GSK3β promotes tumorigenesis by phosphorylating KDM1A and inducing its deubiquitylation by USP22. Nat Cell Biol. 2016;18(9):954‐966. 27501329 10.1038/ncb3396 PMC5026327

[213] Wang Y , Sun Q , Mu N , et al. The deubiquitinase USP22 regulates PD‐L1 degradation in human cancer cells. Cell Commun Signal. 2020;18(1):112. 32665011 10.1186/s12964-020-00612-y PMC7362500

[214] Ning Z , Guo X , Liu X , et al. USP22 regulates lipidome accumulation by stabilizing PPARγ in hepatocellular carcinoma. Nat Commun. 2022;13(1):2187. 35449157 10.1038/s41467-022-29846-9 PMC9023467

[215] Melo‐Cardenas J , Xu Y , Wei J , et al. USP22 deficiency leads to myeloid leukemia upon oncogenic Kras activation through a PU.1‐dependent mechanism. Blood. 2018;132(4):423‐434. 29844011 10.1182/blood-2017-10-811760 PMC6071563

[216] Nelson JK , Thin MZ , Evan T , et al. USP25 promotes pathological HIF‐1‐driven metabolic reprogramming and is a potential therapeutic target in pancreatic cancer. Nat Commun. 2022;13(1):2070. 35440539 10.1038/s41467-022-29684-9 PMC9018856

[217] Dirac AM , Bernards R . The deubiquitinating enzyme USP26 is a regulator of androgen receptor signaling. Mol Cancer Res. 2010;8(6):844‐854. 20501646 10.1158/1541-7786.MCR-09-0424

[218] Li L , Zhou H , Zhu R , Liu Z . USP26 promotes esophageal squamous cell carcinoma metastasis through stabilizing Snail. Cancer Lett. 2019;448:52‐60. 30763716 10.1016/j.canlet.2019.02.007

[219] Dong L , Yu L , Bai C , et al. USP27‐mediated Cyclin E stabilization drives cell cycle progression and hepatocellular tumorigenesis. Oncogene. 2018;37(20):2702‐2713. 29497124 10.1038/s41388-018-0137-z PMC5955865

[220] Diefenbacher ME , Popov N , Blake SM , et al. The deubiquitinase USP28 controls intestinal homeostasis and promotes colorectal cancer. J Clin Invest. 2014;124(8):3407‐3418. 24960159 10.1172/JCI73733 PMC4109555

[221] Diefenbacher ME , Chakraborty A , Blake SM , et al. Usp28 counteracts Fbw7 in intestinal homeostasis and cancer. Cancer Res. 2015;75(7):1181‐1186. 25716680 10.1158/0008-5472.CAN-14-1726 PMC4384988

[222] Haq S , Das S , Kim DH , et al. The stability and oncogenic function of LIN28A are regulated by USP28. Biochim Biophys Acta Mol Basis Dis. 2019;1865(3):599‐610. 30543854 10.1016/j.bbadis.2018.12.006

[223] Wu Y , Wang Y , Yang XH , et al. The deubiquitinase USP28 stabilizes LSD1 and confers stem‐cell‐like traits to breast cancer cells. Cell Rep. 2013;5(1):224‐236. 24075993 10.1016/j.celrep.2013.08.030 PMC4004762

[224] Fong CS , Mazo G , Das T , et al. 53BP1 and USP28 mediate p53‐dependent cell cycle arrest in response to centrosome loss and prolonged mitosis. eLife. 2016;5:e16270. 27371829 10.7554/eLife.16270 PMC4946878

[225] Wu Y , Zhang Y , Wang D , et al. USP29 enhances chemotherapy‐induced stemness in non‐small cell lung cancer via stabilizing Snail1 in response to oxidative stress. Cell Death Dis. 2020;11(9):796. 32968046 10.1038/s41419-020-03008-5 PMC7511960

[226] Martín Y , Cabrera E , Amoedo H , Hernández‐Pérez S , Domínguez‐Kelly R , Freire R . USP29 controls the stability of checkpoint adaptor Claspin by deubiquitination. Oncogene. 2015;34(8):1058‐1063. 24632611 10.1038/onc.2014.38

[227] Liu J , Chung HJ , Vogt M , et al. JTV1 co‐activates FBP to induce USP29 transcription and stabilize p53 in response to oxidative stress. EMBO J. 2011;30(5):846‐858. 21285945 10.1038/emboj.2011.11 PMC3049210

[228] Gu L , Zhu Y , Lin X , et al. Amplification of glyceronephosphate O‐acyltransferase and recruitment of USP30 stabilize DRP1 to promote hepatocarcinogenesis. Cancer Res. 2018;78(20):5808‐5819. 30143522 10.1158/0008-5472.CAN-18-0340

[229] Liang JR , Martinez A , Lane JD , Mayor U , Clague MJ . Urbé S. USP30 deubiquitylates mitochondrial Parkin substrates and restricts apoptotic cell death. EMBO Rep. 2015;16(5):618‐627. 25739811 10.15252/embr.201439820 PMC4428036

[230] Wang L , Wang J , Ma X , et al. USP35 promotes HCC development by stabilizing ABHD17C and activating the PI3K/AKT signaling pathway. Cell Death Discov. 2023;9(1):421. 37993419 10.1038/s41420-023-01714-5 PMC10665393

[231] Tang Z , Jiang W , Mao M , Zhao J , Chen J , Cheng N . Deubiquitinase USP35 modulates ferroptosis in lung cancer via targeting ferroportin. Clin Transl Med. 2021;11(4):e390. 33931967 10.1002/ctm2.390 PMC8087931

[232] Liu C , Wang L , Chen W , et al. USP35 activated by miR let‐7a inhibits cell proliferation and NF‐κB activation through stabilization of ABIN‐2. Oncotarget. 2015;6(29):27891‐22906. 26348204 10.18632/oncotarget.4451 PMC4695033

[233] Sun XX , He X , Yin L , Komada M , Sears RC , Dai MS . The nucleolar ubiquitin‐specific protease USP36 deubiquitinates and stabilizes c‐Myc. Proc Nat Acad Sci USA. 2015;112(12):3734‐3739. 25775507 10.1073/pnas.1411713112 PMC4378440

[234] Fraile JM , Campos‐Iglesias D , Rodríguez F , et al. Loss of the deubiquitinase USP36 destabilizes the RNA helicase DHX33 and causes preimplantation lethality in mice. J Biol Chem. 2018;293(6):2183‐2194. 29273634 10.1074/jbc.M117.788430 PMC5808777

[235] Taillebourg E , Gregoire I , Viargues P , et al. The deubiquitinating enzyme USP36 controls selective autophagy activation by ubiquitinated proteins. Autophagy. 2012;8(5):767‐779. 22622177 10.4161/auto.19381

[236] Zhuang T , Zhang S , Liu D , et al. USP36 promotes tumorigenesis and tamoxifen resistance in breast cancer by deubiquitinating and stabilizing ERα. Exp Clin Cancer Res. 2024;43(1):249. 10.1186/s13046-024-03160-2 PMC11365244 39215346

[237] Pan J , Deng Q , Jiang C , et al. USP37 directly deubiquitinates and stabilizes c‐Myc in lung cancer. Oncogene. 2015;34(30):3957‐3967. 25284584 10.1038/onc.2014.327

[238] Yang WC , Shih HM . The deubiquitinating enzyme USP37 regulates the oncogenic fusion protein PLZF/RARA stability. Oncogene. 2013;32(43):5167‐5175. 23208507 10.1038/onc.2012.537

[239] Qin T , Li B , Feng X , et al. Abnormally elevated USP37 expression in breast cancer stem cells regulates stemness, epithelial‐mesenchymal transition and cisplatin sensitivity. Exp Clin Cancer Res. 2018;37(1):287. 10.1186/s13046-018-0934-9 PMC6258492 30482232

[240] Wu L , Zhao N , Zhou Z , et al. PLAGL2 promotes the proliferation and migration of gastric cancer cells via USP37‐mediated deubiquitination of Snail1. Theranostics. 2021;11(2):700‐714. 33391500 10.7150/thno.47800 PMC7738862

[241] Kim JO , Kim SR , Lim KH , et al. Deubiquitinating enzyme USP37 regulating oncogenic function of 14‐3‐3γ. Oncotarget. 2015;6(34):36551‐36576. 26427597 10.18632/oncotarget.5336 PMC4742195

[242] Das CM , Taylor P , Gireud M , et al. The deubiquitylase USP37 links REST to the control of p27 stability and cell proliferation. Oncogene. 2016;35(47):6153‐6154. 27425592 10.1038/onc.2016.141

[243] Zhang T , Su F , Wang B , et al. Ubiquitin specific peptidase 38 epigenetically regulates KLF transcription factor 5 to augment malignant progression of lung adenocarcinoma. Oncogene. 2024;43(16):1190‐1202. 38409551 10.1038/s41388-024-02985-7

[244] Liu W , Zhang Q , Fang Y , Wang Y . The deubiquitinase USP38 affects cellular functions through interacting with LSD1. Biol Res. 2018;51(1):53. 30497519 10.1186/s40659-018-0201-8 PMC6263071

[245] Li X , Yuan J , Song C , et al. Deubiquitinase USP39 and E3 ligase TRIM26 balance the level of ZEB1 ubiquitination and thereby determine the progression of hepatocellular carcinoma. Cell Death Differ. 2021;28(8):2315‐2332. 33649471 10.1038/s41418-021-00754-7 PMC8329202

[246] Wang W , Lei Y , Zhang G , et al. USP39 stabilizes β‐catenin by deubiquitination and suppressing E3 ligase TRIM26 pre‐mRNA maturation to promote HCC progression. Cell Death Dis. 2023;14(1):63. 36707504 10.1038/s41419-023-05593-7 PMC9883245

[247] Li S , Wang D , Zhao J , Weathington NM , Shang D , Zhao Y . The deubiquitinating enzyme USP48 stabilizes TRAF2 and reduces E‐cadherin‐mediated adherens junctions. FASEB J. 2018;32(1):230‐242. 28874458 10.1096/fj.201700415RR PMC5731130

[248] Zhou A , Lin K , Zhang S , et al. Gli1‐induced deubiquitinase USP48 aids glioblastoma tumorigenesis by stabilizing Gli1. EMBO Rep. 2017;18(8):1318‐1330. 28623188 10.15252/embr.201643124 PMC5538423

[249] Baietti MF , Simicek M , Abbasi Asbagh L , et al. OTUB1 triggers lung cancer development by inhibiting RAS monoubiquitination. EMBO Mol Med. 2016;8(3):288‐303. 26881969 10.15252/emmm.201505972 PMC4772950

[250] Zhou H , Liu Y , Zhu R , et al. OTUB1 promotes esophageal squamous cell carcinoma metastasis through modulating Snail stability. Oncogene. 2018;37(25):3356‐3368. 29559747 10.1038/s41388-018-0224-1

[251] Ye D , Wang S , Wang X , Lin Y , Huang Y , Chi P . Overexpression of OTU domain‐containing ubiquitin aldehyde‐binding protein 1 exacerbates colorectal cancer malignancy by inhibiting protein degradation of β‐Catenin via ubiquitin‐proteasome pathway. Bioengineered. 2022;13(4):9106‐9116. 35354355 10.1080/21655979.2022.2057897 PMC9161894

[252] Iglesias‐Gato D , Chuan YC , Jiang N , et al. OTUB1 de‐ubiquitinating enzyme promotes prostate cancer cell invasion in vitro and tumorigenesis in vivo. Mol Cancer. 2015;14(1):8. 25623341 10.1186/s12943-014-0280-2 PMC4320819

[253] Wang J , Liu Y , Wu D , et al. OTUB1 targets CHK1 for deubiquitination and stabilization to facilitate lung cancer progression and radioresistance. Int J Radiat Oncol Biol Phys. 2024;119(4):1222‐1233. 38266782 10.1016/j.ijrobp.2024.01.202

[254] Xu Y , Xu M , Tong J , et al. Targeting the Otub1/c‐Maf axis for the treatment of multiple myeloma. Blood. 2021;137(11):1478‐1490. 32842143 10.1182/blood.2020005199

[255] Liu T , Jiang L , Tavana O , Gu W . The deubiquitylase OTUB1 mediates ferroptosis via stabilization of SLC7A11. Cancer Res. 2019;79(8):1913‐1924. 30709928 10.1158/0008-5472.CAN-18-3037 PMC6467774

[256] Zhao Y , Ruan J , Li Z , et al. OTUB1 inhibits breast cancer by non‐canonically stabilizing CCN6. Clin Transl Med. 2023;13(8):e1385. 37608493 10.1002/ctm2.1385 PMC10444971

[257] Lee SG , Woo SM , Seo SU , et al. Non‐canonical deubiquitination of OTUB1 induces IFNγ‐mediated cell cycle arrest via regulation of p27 stability. Oncogene. 2024;43(24):1852‐1860. 38664499 10.1038/s41388-024-03042-z PMC11164677

[258] Seo SU , Woo SM , Kim S , et al. Inhibition of cathepsin K sensitizes oxaliplatin‐induced apoptotic cell death by Bax upregulation through OTUB1‐mediated p53 stabilization in vitro and in vivo. Oncogene. 2022;41(4):550‐559. 34785775 10.1038/s41388-021-02088-7 PMC8782718

[259] Li J , Cheng D , Zhu M , et al. OTUB2 stabilizes U2AF2 to promote the Warburg effect and tumorigenesis via the AKT/mTOR signaling pathway in non‐small cell lung cancer. Theranostics. 2019;9(1):179‐195. 30662561 10.7150/thno.29545 PMC6332791

[260] Hu G , Yang J , Zhang H , Huang Z , Yang H . OTUB2 promotes proliferation and migration of hepatocellular carcinoma cells by PJA1 deubiquitylation. Cell Mol Bioeng. 2022;15(3):281‐292. 35611163 10.1007/s12195-022-00720-4 PMC9124278

[261] Gu ZL , Huang J , Zhen LL . Knockdown of otubain 2 inhibits liver cancer cell growth by suppressing NF‐κB signaling. Kaohsiung J Med Sci. 2020;36(6):399‐404. 32003539 10.1002/kjm2.12187 PMC11896491

[262] Liu L , Cheng H , Ji M , et al. OTUB2 regulates YAP1/TAZ to promotes the progression of esophageal squamous cell carcinoma. Biol Proced Online. 2022;24(1):10. 35850645 10.1186/s12575-022-00169-9 PMC9290284

[263] Wan Q , Chen Q , Cai D , Zhao Y , Wu X . OTUB2 promotes homologous recombination repair through stimulating Rad51 expression in endometrial cancer. Cell Transplant. 2020;29:963689720931433. 32830515 10.1177/0963689720931433 PMC7563931

[264] Yu S , Zang W , Qiu Y , Liao L , Zheng X . Deubiquitinase OTUB2 exacerbates the progression of colorectal cancer by promoting PKM2 activity and glycolysis. Oncogene. 2022;41(1):46‐56. 34671086 10.1038/s41388-021-02071-2

[265] Chang W , Luo Q , Wu X , et al. OTUB2 exerts tumor‐suppressive roles via STAT1‐mediated CALML3 activation and increased phosphatidylserine synthesis. Cell Rep. 2022;41(4):111561. 36288705 10.1016/j.celrep.2022.111561

[266] Kato K , Nakajima K , Ui A , Muto‐Terao Y , Ogiwara H , Nakada S . Fine‐tuning of DNA damage‐dependent ubiquitination by OTUB2 supports the DNA repair pathway choice. Mol Cell. 2014;53(4):617‐630. 24560272 10.1016/j.molcel.2014.01.030

[267] Nan Y , Wu X , Luo Q , et al. OTUB2 silencing promotes ovarian cancer via mitochondrial metabolic reprogramming and can be synthetically targeted by CA9 inhibition. Proc Nat Acad Sci USA. 2024;121(19):e2315348121. 38701117 10.1073/pnas.2315348121 PMC11087800

[268] Lin H , Han Y , Sang Y , et al. OTUD1 enhances gastric cancer aggressiveness by deubiquitinating EBV‐encoded protein BALF1 to stabilize the apoptosis inhibitor Bcl‐2. Biochim Biophys Acta Mol Basis Dis. 2024;1870(5):167132. 38565386 10.1016/j.bbadis.2024.167132

[269] Oikawa D , Gi M , Kosako H , et al. OTUD1 deubiquitinase regulates NF‐κB‐ and KEAP1‐mediated inflammatory responses and reactive oxygen species‐associated cell death pathways. Cell Death Dis. 2022;13(8):694. 35941131 10.1038/s41419-022-05145-5 PMC9360000

[270] Grattarola M , Cucci MA , Roetto A , Dianzani C , Barrera G , Pizzimenti S . Post‐translational down‐regulation of Nrf2 and YAP proteins, by targeting deubiquitinases, reduces growth and chemoresistance in pancreatic cancer cells. Free Radical Biol Med. 2021;174:202‐210. 34364982 10.1016/j.freeradbiomed.2021.08.006

[271] Li JJ , Wang JH , Tian T , et al. The liver microenvironment orchestrates FGL1‐mediated immune escape and progression of metastatic colorectal cancer. Nat Commun. 2023;14(1):6690. 37872170 10.1038/s41467-023-42332-0 PMC10593839

[272] Ma X , Wang L , Shi G , Sun S . The deubiquitinase OTUD1 inhibits non‐small cell lung cancer progression by deubiquitinating and stabilizing KLF4. Thoracic Cancer. 2022;13(5):761‐770. 35098684 10.1111/1759-7714.14320 PMC8888149

[273] Zhang Q , Li J , Chen Z , et al. VE‐822 upregulates the deubiquitinase OTUD1 to stabilize FHL1 to inhibit the progression of lung adenocarcinoma. Cell Oncol (Dordr). 2023;46(4):1001‐1014. 36929488 10.1007/s13402-023-00793-x PMC12974673

[274] Luo Q , Wu X , Zhao P , et al. OTUD1 activates caspase‐independent and caspase‐dependent apoptosis by promoting AIF nuclear translocation and MCL1 degradation. Adv Sci (Weinh). 2021;8(8):2002874. 33898171 10.1002/advs.202002874 PMC8061361

[275] Wu L , Lin Y , Feng J , et al. The deubiquitinating enzyme OTUD1 antagonizes BH3‐mimetic inhibitor induced cell death through regulating the stability of the MCL1 protein. Cancer Cell Int. 2019;19:222. 31467488 10.1186/s12935-019-0936-5 PMC6712616

[276] Liu W , Yan B , Yu H , et al. OTUD1 stabilizes PTEN to inhibit the PI3K/AKT and TNF‐alpha/NF‐kappaB signaling pathways and sensitize ccRCC to TKIs. Int J Biol Sci. 2022;18(4):1401‐1414. 35280681 10.7150/ijbs.68980 PMC8898358

[277] Zhou T , Wu Y , Qian D , et al. OTUD1 chemosensitizes triple‐negative breast cancer to doxorubicin by modulating P16 expression. Pathol Res Pract. 2023;247:154571. 37257246 10.1016/j.prp.2023.154571

[278] Liu H , Zhong L , Lu Y , et al. Deubiquitylase OTUD1 confers Erlotinib sensitivity in non‐small cell lung cancer through inhibition of nuclear translocation of YAP1. Cell Death Discov. 2022;8(1):403. 36182943 10.1038/s41420-022-01119-w PMC9526728

[279] Song J , Liu T , Yin Y , et al. The deubiquitinase OTUD1 enhances iron transport and potentiates host antitumor immunity. EMBO Rep. 2021;22(2):e51162. 33393230 10.15252/embr.202051162 PMC7857436

[280] Woo SM , Seo SU , Min KJ , Kwon TK . Melatonin induces apoptotic cell death through Bim stabilization by Sp1‐mediated OTUD1 upregulation. J Pineal Res. 2022;72(1):e12781. 34826170 10.1111/jpi.12781

[281] Han Z , Jia Q , Zhang J , et al. Deubiquitylase YOD1 regulates CDK1 stability and drives triple‐negative breast cancer tumorigenesis. J Exp Clin Cancer Res. 2023;42(1):228. 37667382 10.1186/s13046-023-02781-3 PMC10478497

[282] Park SS , Baek KH . Synergistic effect of YOD1 and USP21 on the Hippo signaling pathway. Cancer Cell Int. 2023;23(1):209. 37743467 10.1186/s12935-023-03078-3 PMC10518088

[283] Pu J , Xu Z , Nian J , et al. M2 macrophage‐derived extracellular vesicles facilitate CD8+T cell exhaustion in hepatocellular carcinoma via the miR‐21‐5p/YOD1/YAP/β‐catenin pathway. Cell Death Discov. 2021;7(1):182. 34282135 10.1038/s41420-021-00556-3 PMC8289864

[284] Wu Y , Duan Y , Han W , et al. Deubiquitinase YOD1 suppresses tumor progression by stabilizing E3 ligase TRIM33 in head and neck squamous cell carcinoma. Cell Death Dis. 2023;14(8):517. 37573347 10.1038/s41419-023-06035-0 PMC10423255

[285] Ci M , Zhao G , Li C , et al. OTUD4 promotes the progression of glioblastoma by deubiquitinating CDK1 and activating MAPK signaling pathway. Cell Death Dis. 2024;15(3):179. 38429268 10.1038/s41419-024-06569-x PMC10907623

[286] Gao Y , Tang J , Ma X , et al. OTUD4 regulates metastasis and chemoresistance in melanoma by stabilizing Snail1. J Cell Physiol. 2023;238(11):2546‐2555. 37642406 10.1002/jcp.31104

[287] Cui X , Shang X , Xie J , et al. Cooperation between IRTKS and deubiquitinase OTUD4 enhances the SETDB1‐mediated H3K9 trimethylation that promotes tumor metastasis via suppressing E‐cadherin expression. Cancer Lett. 2023;575:216404. 37739210 10.1016/j.canlet.2023.216404

[288] Li Z , Tian Y , Zong H , et al. Deubiquitinating enzyme OTUD4 stabilizes RBM47 to induce ATF3 transcription: a novel mechanism underlying the restrained malignant properties of ccRCC cells. Apoptosis. 2024;29(7‐8):1051‐1069. 38553613 10.1007/s10495-024-01953-6

[289] Di M , Miao J , Pan Q , et al. OTUD4‐mediated GSDME deubiquitination enhances radiosensitivity in nasopharyngeal carcinoma by inducing pyroptosis. J Exp Clin Cancer Res. 2022;41(1):328. 36411454 10.1186/s13046-022-02533-9 PMC9677691

[290] Marchese E , Demehri S . Posttranslational protein modifications as gatekeepers of cancer immunogenicity. J Clin Invest. 2024;134(10):e180914. 38747288 10.1172/JCI180914 PMC11093601

[291] Wu Z , Qiu M , Guo Y , et al. OTU deubiquitinase 4 is silenced and radiosensitizes non‐small cell lung cancer cells via inhibiting DNA repair. Cancer Cell Int. 2019;19:99. 31011293 10.1186/s12935-019-0816-z PMC6466656

[292] Zhang Y , Fan Y , Jing X , et al. OTUD5‐mediated deubiquitination of YAP in macrophage promotes M2 phenotype polarization and favors triple‐negative breast cancer progression. Cancer Lett. 2021;504:104‐115. 33587979 10.1016/j.canlet.2021.02.003

[293] Yang Y , Jia S , Zhu N , et al. OTUD5 promotes the growth of hepatocellular carcinoma by deubiquitinating and stabilizing SLC38A1. Biol Direct. 2024;19(1):31. 38658981 10.1186/s13062-024-00475-0 PMC11041014

[294] Hou T , Dan W , Liu T , et al. Deubiquitinase OTUD5 modulates mTORC1 signaling to promote bladder cancer progression. Cell Death Dis. 2022;13(9):778. 36085200 10.1038/s41419-022-05128-6 PMC9463452

[295] Li X , Lu B , Zhang L , Yang J , Cheng Y , Yan D . Mechanism of OTUD5 in non‐small cell lung cancer cell proliferation, invasion, and migration. Bosn J Basic Med Sci. 2022;22(6):901‐911. 35765958 10.17305/bjbms.2022.7206 PMC9589305

[296] Li F , Sun Q , Liu K , et al. OTUD5 cooperates with TRIM25 in transcriptional regulation and tumor progression via deubiquitination activity. Nat Commun. 2020;11(1):4184. 32826889 10.1038/s41467-020-17926-7 PMC7442798

[297] de Vivo A , Sanchez A , Yegres J , Kim J , Emly S , Kee Y . The OTUD5‐UBR5 complex regulates FACT‐mediated transcription at damaged chromatin. Nucleic Acids Res. 2019;47(2):729‐746. 30508113 10.1093/nar/gky1219 PMC6344881

[298] Guo Y , Jiang F , Kong L , et al. OTUD5 promotes innate antiviral and antitumor immunity through deubiquitinating and stabilizing STING. Cell Mol Immunol. 2021;18(8):1945‐1955. 32879469 10.1038/s41423-020-00531-5 PMC8322343

[299] Tang J , Wu Z , Tian Z , Chen W , Wu G . OTUD7B stabilizes estrogen receptor α and promotes breast cancer cell proliferation. Cell Death Dis. 2021;12(6):534. 34035221 10.1038/s41419-021-03785-7 PMC8149656

[300] Lin DD , Shen Y , Qiao S , et al. Upregulation of OTUD7B (Cezanne) promotes tumor progression via AKT/VEGF pathway in lung squamous carcinoma and adenocarcinoma. Front Oncol. 2019;9:862. 31572671 10.3389/fonc.2019.00862 PMC6749047

[301] Chen S , Cai K , Zheng D , et al. RHBDL2 promotes the proliferation, migration, and invasion of pancreatic cancer by stabilizing the N1ICD via the OTUD7B and activating the Notch signaling pathway. Cell Death Dis. 2022;13(11):945. 36351890 10.1038/s41419-022-05379-3 PMC9646733

[302] Bonacci T , Suzuki A , Grant GD , et al. Cezanne/OTUD7B is a cell cycle‐regulated deubiquitinase that antagonizes the degradation of APC/C substrates. EMBO J. 2018;37(16):e98701. 29973362 10.15252/embj.201798701 PMC6092620

[303] Wang Y , Li J , Zheng H , et al. Cezanne promoted autophagy through PIK3C3 stabilization and PIK3C2A transcription in lung adenocarcinoma. Cell Death Discov. 2023;9(1):302. 37596251 10.1038/s41420-023-01599-4 PMC10439204

[304] Sun C , Bai J , Sun J , et al. OTU deubiquitinase 7B facilitates the hyperthermia‐induced inhibition of lung cancer progression through enhancing Smac‐mediated mitochondrial dysfunction. Environ Toxicol. 2024;39(4):1989‐2005. 38088504 10.1002/tox.24080

[305] Lee E , Ouzounova M , Piranlioglu R , et al. The pleiotropic effects of TNFα in breast cancer subtypes is regulated by TNFAIP3/A20. Oncogene. 2019;38(4):469‐482. 30166590 10.1038/s41388-018-0472-0 PMC6602794

[306] Lee JH , Jung SM , Yang KM , et al. A20 promotes metastasis of aggressive basal‐like breast cancers through multi‐monoubiquitylation of Snail1. Nat Cell Biol. 2017;19(10):1260‐1273. 28892081 10.1038/ncb3609

[307] Ma J , Wang H , Guo S , et al. A20 promotes melanoma progression via the activation of Akt pathway. Cell Death Dis. 2020;11(9):794. 32968045 10.1038/s41419-020-03001-y PMC7511359

[308] Feng Y , Zhang Y , Cai Y , et al. A20 targets PFKL and glycolysis to inhibit the progression of hepatocellular carcinoma. Cell Death Dis. 2020;11(2):89. 32015333 10.1038/s41419-020-2278-6 PMC6997366

[309] Luo M , Wang X , Wu S , et al. A20 promotes colorectal cancer immune evasion by upregulating STC1 expression to block “eat‐me” signal. Signal Transduct Target Ther. 2023;8(1):312. 37607946 10.1038/s41392-023-01545-x PMC10444827

[310] Chen H , Hu L , Luo Z , et al. A20 suppresses hepatocellular carcinoma proliferation and metastasis through inhibition of Twist1 expression. Mol Cancer. 2015;14:186. 26538215 10.1186/s12943-015-0454-6 PMC4634191

[311] Zhou X , An D , Liu X , Jiang M , Yuan C , Hu J . TNFα induces tolerant production of CXC chemokines in colorectal cancer HCT116 cells via A20 inhibition of ERK signaling. Int Immunopharmacol. 2018;54:296‐302. 29175508 10.1016/j.intimp.2017.11.027

[312] Wang X , Xiao Y , Dong Y , et al. A20 interacts with mTORC2 to inhibit the mTORC2/Akt/Rac1 signaling axis in hepatocellular carcinoma cells. Cancer Gene Ther. 2023;30(3):424‐436. 36411371 10.1038/s41417-022-00562-2

[313] Damgaard RB , Jolin HE , Allison MED , et al. OTULIN protects the liver against cell death, inflammation, fibrosis, and cancer. Cell Death Differ. 2020;27(5):1457‐1474. 32231246 10.1038/s41418-020-0532-1 PMC7206033

[314] Verboom L , Martens A , Priem D , et al. OTULIN prevents liver inflammation and hepatocellular carcinoma by inhibiting FADD‐ and RIPK1 kinase‐mediated hepatocyte apoptosis. Cell Rep. 2020;30(7):2237‐2247. 32075762 10.1016/j.celrep.2020.01.028

[315] Yan Q , Shi S , Ge Y , Wan S , Li M , Li M . UCHL1 alleviates apoptosis in chondrocytes via upregulation of HIF‑1α‑mediated mitophagy. Int J Mol Med. 2023;52(4):99. 37681473 10.3892/ijmm.2023.5302 PMC10555477

[316] Brinkmann K , Zigrino P , Witt A , et al. Ubiquitin C‐terminal hydrolase‐L1 potentiates cancer chemosensitivity by stabilizing NOXA. Cell Rep. 2013;3(3):881‐891. 23499448 10.1016/j.celrep.2013.02.014

[317] Li L , Tao Q , Jin H , et al. The tumor suppressor UCHL1 forms a complex with p53/MDM2/ARF to promote p53 signaling and is frequently silenced in nasopharyngeal carcinoma. Clin Cancer Res. 2010;16(11):2949‐2958. 20395212 10.1158/1078-0432.CCR-09-3178

[318] Liao C , Beveridge R , Hudson JJR , et al. UCHL3 regulates topoisomerase‐induced chromosomal break repair by controlling TDP1 proteostasis. Cell Rep. 2018;23(11):3352‐3365. 29898404 10.1016/j.celrep.2018.05.033 PMC6019701

[319] Ouyang L , Yan B , Liu Y , et al. The deubiquitylase UCHL3 maintains cancer stem‐like properties by stabilizing the aryl hydrocarbon receptor. Signal Transduct Target Ther. 2020;5(1):78. 32546741 10.1038/s41392-020-0181-3 PMC7297794

[320] Qin J , Zhou Z , Chen W , et al. BAP1 promotes breast cancer cell proliferation and metastasis by deubiquitinating KLF5. Nat Commun. 2015;6:8471. 26419610 10.1038/ncomms9471 PMC4598844

[321] Peng J , Ma J , Li W , et al. Stabilization of MCRS1 by BAP1 prevents chromosome instability in renal cell carcinoma. Cancer Lett. 2015;369(1):167‐174. 26300492 10.1016/j.canlet.2015.08.013

[322] Sahtoe DD , van Dijk WJ , Ekkebus R , Ovaa H , Sixma TK . BAP1/ASXL1 recruitment and activation for H2A deubiquitination. Nat Commun. 2016;7:10292. 26739236 10.1038/ncomms10292 PMC4729829

[323] Zarrizi R , Menard JA , Belting M , Massoumi R . Deubiquitination of γ‐tubulin by BAP1 prevents chromosome instability in breast cancer cells. Cancer Res. 2014;74(22):6499‐6508. 25228651 10.1158/0008-5472.CAN-14-0221

[324] Xie Z , Lin H , Huang Y , et al. BAP1‐mediated MAFF deubiquitylation regulates tumor growth and is associated with adverse outcomes in colorectal cancer. Eur J Cancer. 2024;210:114278. 39151323 10.1016/j.ejca.2024.114278

[325] Wang B , Ma A , Zhang L , et al. POH1 deubiquitylates and stabilizes E2F1 to promote tumour formation. Nat Commun. 2015;6:8704. 26510456 10.1038/ncomms9704 PMC4846323

[326] Seo D , Jung SM , Park JS , et al. The deubiquitinating enzyme PSMD14 facilitates tumor growth and chemoresistance through stabilizing the ALK2 receptor in the initiation of BMP6 signaling pathway. EBioMedicine. 2019;49:55‐71. 31685442 10.1016/j.ebiom.2019.10.039 PMC7113187

[327] Kakarougkas A , Ismail A , Katsuki Y , Freire R , Shibata A , Jeggo PA . Co‐operation of BRCA1 and POH1 relieves the barriers posed by 53BP1 and RAP80 to resection. Nucleic Acids Res. 2013;41(22):10298‐10311. 24013561 10.1093/nar/gkt802 PMC3905848

[328] Harbeck N , Gnant M . Breast cancer. Lancet (London, England). 2017;389(10074):1134‐1150. 27865536 10.1016/S0140-6736(16)31891-8

[329] Liu T , Yu J , Deng M , et al. CDK4/6‐dependent activation of DUB3 regulates cancer metastasis through SNAIL1. Nat Commun. 2017;8:13923. 28067227 10.1038/ncomms13923 PMC5228031

[330] Tang J , Luo Y , Long G , Zhou L . MINDY1 promotes breast cancer cell proliferation by stabilizing estrogen receptor α. Cell Death Dis. 2021;12(10):937. 34645792 10.1038/s41419-021-04244-z PMC8514509

[331] Hirsch FR , Scagliotti GV , Mulshine JL , et al. Lung cancer: current therapies and new targeted treatments. Lancet (London, England). 2017;389(10066):299‐311. 27574741 10.1016/S0140-6736(16)30958-8

[332] Zhang P , Li C , Li H , et al. Ubiquitin ligase CHIP regulates OTUD3 stability and suppresses tumour metastasis in lung cancer. Cell Death Differ. 2020;27(11):3177‐3195. 32483383 10.1038/s41418-020-0571-7 PMC7560627

[333] Vogel A , Meyer T , Sapisochin G , Salem R , Saborowski A . Hepatocellular carcinoma. Lancet (London, England). 2022;400(10360):1345‐1362. 36084663 10.1016/S0140-6736(22)01200-4

[334] Wang JH , Zhong XP , Zhang YF , et al. Cezanne predicts progression and adjuvant TACE response in hepatocellular carcinoma. Cell Death Dis. 2017;8(9):e3043. 28880268 10.1038/cddis.2017.428 PMC5636974

[335] Xu Z , Pei L , Wang L , Zhang F , Hu X , Gui Y . Snail1‐dependent transcriptional repression of Cezanne2 in hepatocellular carcinoma. Oncogene. 2014;33(22):2836‐2845. 23792447 10.1038/onc.2013.243

[336] Dekker E , Tanis PJ , Vleugels JLA , Kasi PM , Wallace MB . Colorectal cancer. Lancet (London, England). 2019;394(10207):1467‐1480. 31631858 10.1016/S0140-6736(19)32319-0

[337] Zhang Q , Zhang ZY , Du H , et al. DUB3 deubiquitinates and stabilizes NRF2 in chemotherapy resistance of colorectal cancer. Cell Death Differ. 2019;26(11):2300‐2313. 30778200 10.1038/s41418-019-0303-z PMC6889501

[338] Liao C , Wang Q , An J , et al. Partial EMT in squamous cell carcinoma: a snapshot. Int J Biol Sci. 2021;17(12):3036‐3047. 34421348 10.7150/ijbs.61566 PMC8375241

[339] Wang WP , Shi D , Yun D , et al. Role of deubiquitinase JOSD2 in the pathogenesis of esophageal squamous cell carcinoma. World J Gastroenterol. 2024;30(6):565‐578. 38463028 10.3748/wjg.v30.i6.565 PMC10921146

[340] Li Y , Li R , Qin H , He H , Li S . OTUB1's role in promoting OSCC development by stabilizing RACK1 involves cell proliferation, migration, invasion, and tumor‐associated macrophage M1 polarization. Cell Signalling. 2023;110:110835. 37532135 10.1016/j.cellsig.2023.110835

[341] Jin S , Tsunematsu T , Horiguchi T , et al. Involvement of the OTUB1‐YAP1 axis in driving malignant behaviors of head and neck squamous cell carcinoma. Cancer Med. 2023;12(24):22156‐22169. 37986681 10.1002/cam4.6735 PMC10757095

[342] Yu J , Yuan S , Song J , Yu S . USP39 interacts with SIRT7 to promote cervical squamous cell carcinoma by modulating autophagy and oxidative stress via FOXM1. J Transl Med. 2023;21(1):807. 37957720 10.1186/s12967-023-04623-4 PMC10641974

[343] Peng Y , Liu J , Wang Z , et al. Prostate‐specific oncogene OTUD6A promotes prostatic tumorigenesis via deubiquitinating and stabilizing c‐Myc. Cell Death Differ. 2022;29(9):1730‐1743. 35217790 10.1038/s41418-022-00960-x PMC9433443

[344] Wang YQ , Zhang QY , Weng WW , et al. Upregulation of the non‐coding RNA OTUB1‐isoform 2 contributes to gastric cancer cell proliferation and invasion and predicts poor gastric cancer prognosis. Int J Biol Sci. 2016;12(5):545‐557. 27019636 10.7150/ijbs.13540 PMC4807415

[345] Trivigno D , Essmann F , Huber SM , Rudner J . Deubiquitinase USP9x confers radioresistance through stabilization of Mcl‐1. Neoplasia (New York, NY). 2012;14(10):893‐904. 10.1593/neo.12598 PMC3479835 23097624

[346] Pu Q , Lv YR , Dong K , Geng WW , Gao HD . Tumor suppressor OTUD3 induces growth inhibition and apoptosis by directly deubiquitinating and stabilizing p53 in invasive breast carcinoma cells. BMC Cancer. 2020;20(1):583. 32571254 10.1186/s12885-020-07069-9 PMC7310228

[347] Tian S , Jin S , Wu Y , et al. High‐throughput screening of functional deubiquitinating enzymes in autophagy. Autophagy. 2021;17(6):1367‐1378. 32453962 10.1080/15548627.2020.1761652 PMC8205047

[348] Basu AK . DNA damage, mutagenesis and cancer. Int J Mol Sci. 2018;19(4):970. 29570697 10.3390/ijms19040970 PMC5979367

[349] Castella M , Jacquemont C , Thompson EL , et al. FANCI regulates recruitment of the FA core complex at sites of DNA damage independently of FANCD2. PLos Genet. 2015;11(10):e1005563. 26430909 10.1371/journal.pgen.1005563 PMC4592014

[350] Williams SA , Maecker HL , French DM , et al. USP1 deubiquitinates ID proteins to preserve a mesenchymal stem cell program in osteosarcoma. Cell. 2011;146(6):918‐930. 21925315 10.1016/j.cell.2011.07.040

[351] Wu Q , Huang Y , Gu L , Chang Z , Li GM . OTUB1 stabilizes mismatch repair protein MSH2 by blocking ubiquitination. J Biol Chem. 2021;296:100466. 33640455 10.1016/j.jbc.2021.100466 PMC8042173

[352] Yang C , Zang W , Tang Z , et al. A20/TNFAIP3 regulates the DNA damage response and mediates tumor cell resistance to DNA‐damaging therapy. Cancer Res. 2018;78(4):1069‐1082. 29233925 10.1158/0008-5472.CAN-17-2143

[353] Zhong X , He X , Wang Y , et al. Warburg effect in colorectal cancer: the emerging roles in tumor microenvironment and therapeutic implications. J Hematol Oncol. 2022;15(1):160. 36319992 10.1186/s13045-022-01358-5 PMC9628128

[354] Han X , Ren C , Lu C , Qiao P , Yang T , Yu Z . Deubiquitination of MYC by OTUB1 contributes to HK2 mediated glycolysis and breast tumorigenesis. Cell Death Differ. 2022;29(9):1864‐1873. 35296795 10.1038/s41418-022-00971-8 PMC9433372

[355] Liu J , Chen Z , Li Y , Zhao W , Wu J , Zhang Z . PD‐1/PD‐L1 checkpoint inhibitors in tumor immunotherapy. Front Pharmacol. 2021;12:731798. 34539412 10.3389/fphar.2021.731798 PMC8440961

[356] Breitenecker K , Homolya M , Luca AC , et al. Down‐regulation of A20 promotes immune escape of lung adenocarcinomas. Sci Transl Med. 2021;13(601):eabc3911. 34233950 10.1126/scitranslmed.abc3911 PMC7611502

[357] Guo W , Ma J , Guo S , et al. A20 regulates the therapeutic effect of anti‐PD‐1 immunotherapy in melanoma. J Immunother Cancer. 2020;8(2):e001866. 33298620 10.1136/jitc-2020-001866 PMC7733187

[358] Li J , Yuan S , Norgard RJ , et al. Tumor cell‐intrinsic USP22 suppresses antitumor immunity in pancreatic cancer. Cancer Immunol Res. 2020;8(3):282‐291. 31871120 10.1158/2326-6066.CIR-19-0661 PMC7173406

[359] Shi D , Wu X , Jian Y , et al. USP14 promotes tryptophan metabolism and immune suppression by stabilizing IDO1 in colorectal cancer. Nat Commun. 2022;13(1):5644. 36163134 10.1038/s41467-022-33285-x PMC9513055

[360] Hong B , Li H , Lu Y , et al. USP18 is crucial for IFN‐γ‐mediated inhibition of B16 melanoma tumorigenesis and antitumor immunity. Mol Cancer. 2014;13:132. 24884733 10.1186/1476-4598-13-132 PMC4057584

[361] Zhou X , Yu J , Cheng X , et al. The deubiquitinase Otub1 controls the activation of CD8(+) T cells and NK cells by regulating IL‐15‐mediated priming. Nat Immunol. 2019;20(7):879‐889. 31182807 10.1038/s41590-019-0405-2 PMC6588407

[362] Zhao G , Song D , Wu J , et al. Identification of OTUD6B as a new biomarker for prognosis and immunotherapy by pan‐cancer analysis. Front Immunol. 2022;13:955091. 36052059 10.3389/fimmu.2022.955091 PMC9425067

[363] Amer‐Sarsour F , Kordonsky A , Berdichevsky Y , Prag G , Ashkenazi A . Deubiquitylating enzymes in neuronal health and disease. Cell Death Dis. 2021;12(1):120. 33483467 10.1038/s41419-020-03361-5 PMC7822931

[364] Hwang JT , Lee A , Kho C . Ubiquitin and ubiquitin‐like proteins in cancer, neurodegenerative disorders, and heart diseases. Int J Mol Sci. 2022;23(9):5053. 35563444 10.3390/ijms23095053 PMC9105348

[365] Dugger BN , Dickson DW . Pathology of neurodegenerative diseases. Cold Spring Harb Perspect Biol. 2017;9(7):a028035. 28062563 10.1101/cshperspect.a028035 PMC5495060

[366] Wu S , Lin T , Xu Y . Polymorphic USP8 allele promotes Parkinson's disease by inducing the accumulation of α‐synuclein through deubiquitination. Cell Mol Life Sci. 2023;80(12):363. 37981592 10.1007/s00018-023-05006-0 PMC11072815

[367] Aron R , Pellegrini P , Green EW , et al. Deubiquitinase Usp12 functions noncatalytically to induce autophagy and confer neuroprotection in models of Huntington's disease. Nat Commun. 2018;9(1):3191. 30266909 10.1038/s41467-018-05653-z PMC6162324

[368] Vaden JH , Watson JA , Howard AD , Chen PC , Wilson JA , Wilson SM . Distinct effects of ubiquitin overexpression on NMJ structure and motor performance in mice expressing catalytically inactive USP14. Front Mol Neurosci. 2015;8:11. 25954152 10.3389/fnmol.2015.00011 PMC4407586

[369] Kiprowska MJ , Stepanova A , Todaro DR , et al. Neurotoxic mechanisms by which the USP14 inhibitor IU1 depletes ubiquitinated proteins and Tau in rat cerebral cortical neurons: relevance to Alzheimer's disease. Biochim Biophys Acta Mol Basis Dis. 2017;1863(6):1157‐1170. 28372990 10.1016/j.bbadis.2017.03.017 PMC5549686

[370] Jin YN , Chen PC , Watson JA , et al. Usp14 deficiency increases tau phosphorylation without altering tau degradation or causing tau‐dependent deficits. PLoS One. 2012;7(10):e47884. 23144711 10.1371/journal.pone.0047884 PMC3483306

[371] Lee JH , Shin SK , Jiang Y , et al. Facilitated Tau degradation by USP14 aptamers via enhanced proteasome activity. Sci Rep. 2015;5:10757. 26041011 10.1038/srep10757 PMC4455164

[372] Jarome TJ , Kwapis JL , Hallengren JJ , Wilson SM , Helmstetter FJ . The ubiquitin‐specific protease 14 (USP14) is a critical regulator of long‐term memory formation. Learn Mem (Cold Spring Harbor, NY). 2013;21(1):9‐13. 10.1101/lm.032771.113 PMC3867711 24344179

[373] Min JW , Lü L , Freeling JL , Martin DS , Wang H . USP14 inhibitor attenuates cerebral ischemia/reperfusion‐induced neuronal injury in mice. J Neurochem. 2017;140(5):826‐833. 28029679 10.1111/jnc.13941 PMC5527549

[374] Fang TZ , Sun Y , Pearce AC , et al. Knockout or inhibition of USP30 protects dopaminergic neurons in a Parkinson's disease mouse model. Nat Commun. 2023;14(1):7295. 37957154 10.1038/s41467-023-42876-1 PMC10643470

[375] Leotti VB , de Vries JJ , Oliveira CM , et al. CAG repeat size influences the progression rate of spinocerebellar ataxia type 3. Ann Neurol. 2021;89(1):66‐73. 32978817 10.1002/ana.25919

[376] Nobre RJ , Lobo DD , Henriques C , et al. miRNA‐mediated knockdown of ATXN3 alleviates molecular disease hallmarks in a mouse model for spinocerebellar ataxia type 3. Nucleic Acid Ther. 2022;32(3):194‐205. 34878314 10.1089/nat.2021.0020 PMC9221165

[377] Martier R , Sogorb‐Gonzalez M , Stricker‐Shaver J , et al. Development of an AAV‐based microRNA gene therapy to treat Machado‐Joseph disease. Mol Ther Methods Clin Dev. 2019;15:343‐358. 31828177 10.1016/j.omtm.2019.10.008 PMC6889651

[378] He L , Wang S , Peng L , et al. CRISPR/Cas9 mediated gene correction ameliorates abnormal phenotypes in spinocerebellar ataxia type 3 patient‐derived induced pluripotent stem cells. Transl Psychiatry. 2021;11(1):479. 34535635 10.1038/s41398-021-01605-2 PMC8448778

[379] Cartier AE , Djakovic SN , Salehi A , Wilson SM , Masliah E , Patrick GN . Regulation of synaptic structure by ubiquitin C‐terminal hydrolase L1. J Neurosci. 2009;29(24):7857‐7868. 19535597 10.1523/JNEUROSCI.1817-09.2009 PMC2748938

[380] Mi Z , Graham SH . Role of UCHL1 in the pathogenesis of neurodegenerative diseases and brain injury. Ageing Res Rev. 2023;86:101856. 36681249 10.1016/j.arr.2023.101856 PMC9992267

[381] Liu Y , Fallon L , Lashuel HA , Liu Z , Lansbury PT . The UCH‐L1 gene encodes two opposing enzymatic activities that affect alpha‐synuclein degradation and Parkinson's disease susceptibility. Cell. 2002;111(2):209‐218. 12408865 10.1016/s0092-8674(02)01012-7

[382] Guo YY , Lu Y , Zheng Y , et al. Ubiquitin C‐terminal hydrolase L1 (UCH‐L1) promotes hippocampus‐dependent memory via its deubiquitinating effect on TrkB. J Neurosci. 2017;37(25):5978‐5995. 28500221 10.1523/JNEUROSCI.3148-16.2017 PMC6596500

[383] Zhan X , Yang Y , Li Q , He F . The role of deubiquitinases in cardiac disease. Expert Rev Mol Med. 2024;26:e3. 38525836 10.1017/erm.2024.2 PMC11062144

[384] Xing J , Li P , Hong J , et al. Overexpression of ubiquitin‐specific protease 2 (USP2) in the heart suppressed pressure overload‐induced cardiac remodeling. Mediators Inflamm. 2020;2020:4121750. 32963492 10.1155/2020/4121750 PMC7492881

[385] Klaeske K , Dix M , Adams V , et al. Differential regulation of myocardial E3 ligases and deubiquitinases in ischemic heart failure. Life (Basel, Switzerland). 2021;11(12):1430. 34947961 10.3390/life11121430 PMC8708923

[386] He B , Zhao YC , Gao LC , et al. Ubiquitin‐specific protease 4 is an endogenous negative regulator of pathological cardiac hypertrophy. Hypertension (Dallas, Tex : 1979). 2016;67(6):1237‐1248. 27045030 10.1161/HYPERTENSIONAHA.116.07392

[387] Jiang YN , Yang SX , Guan X , et al. Loss of USP22 alleviates cardiac hypertrophy induced by pressure overload through HiF1‐α‐TAK1 signaling pathway. Biochim Biophys Acta Mol Basis Dis. 2023;1869(8):166813. 37488049 10.1016/j.bbadis.2023.166813

[388] Liu N , Chai R , Liu B , et al. Ubiquitin‐specific protease 14 regulates cardiac hypertrophy progression by increasing GSK‐3β phosphorylation. Biochem Biophys Res Commun. 2016;478(3):1236‐1241. 27545607 10.1016/j.bbrc.2016.08.100

[389] Ye B , Zhou H , Chen Y , et al. USP25 ameliorates pathological cardiac hypertrophy by stabilizing SERCA2a in cardiomyocytes. Circ Res. 2023;132(4):465‐480. 36722348 10.1161/CIRCRESAHA.122.321849

[390] Zhang DH , Zhang JL , Huang Z , et al. Deubiquitinase ubiquitin‐specific protease 10 deficiency regulates Sirt6 signaling and exacerbates cardiac hypertrophy. J Am Heart Assoc. 2020;9(22):e017751. 33170082 10.1161/JAHA.120.017751 PMC7763723

[391] Hu Y , Ma Z , Chen Z , Chen B . USP47 promotes apoptosis in rat myocardial cells after ischemia/reperfusion injury via NF‐κB activation. Biotechnol Appl Biochem. 2021;68(4):841‐848. 32761659 10.1002/bab.2000

[392] Zhang W , Zhang Y , Zhang H , Zhao Q , Liu Z , Xu Y . USP49 inhibits ischemia‐reperfusion‐induced cell viability suppression and apoptosis in human AC16 cardiomyocytes through DUSP1‐JNK1/2 signaling. J Cell Physiol. 2019;234(5):6529‐6538. 30246457 10.1002/jcp.27390

[393] Tang LJ , Zhou YJ , Xiong XM , et al. Ubiquitin‐specific protease 7 promotes ferroptosis via activation of the p53/TfR1 pathway in the rat hearts after ischemia/reperfusion. Free Radical Biol Med. 2021;162:339‐352. 33157209 10.1016/j.freeradbiomed.2020.10.307

[394] Zhang Y , Hailati J , Ma X , Midilibieke H , Liu Z . Ubiquitin‐specific protease 11 aggravates ischemia‐reperfusion‐induced cardiomyocyte pyroptosis and injury by promoting TRAF3 deubiquitination. Balkan Med J. 2023;40(3):205‐214. 37000116 10.4274/balkanmedj.galenos.2023.2022-12-15 PMC10175892

[395] Wu Z , Li W , Wang S , Zheng Z . Role of deubiquitinase USP47 in cardiac function alleviation and anti‐inflammatory immunity after myocardial infarction by regulating NLRP3 inflammasome‐mediated pyroptotic signal pathways. Int Immunopharmacol. 2024;136:112346. 38850785 10.1016/j.intimp.2024.112346

[396] Sun Y , Lu F , Yu X , et al. Exogenous H(2)S promoted USP8 sulfhydration to regulate mitophagy in the hearts of db/db Mice. Aging Dis. 2020;11(2):269‐285. 32257541 10.14336/AD.2019.0524 PMC7069468

[397] Lu L , Ma J , Liu Y , et al. FSTL1‐USP10‐Notch1 signaling axis protects against cardiac dysfunction through inhibition of myocardial fibrosis in diabetic mice. Front Cell Dev Biol. 2021;9:757068. 34957094 10.3389/fcell.2021.757068 PMC8695978

[398] Parihar N , Bhatt LK . Deubiquitylating enzymes: potential target in autoimmune diseases. Inflammopharmacology. 2021;29(6):1683‐1699. 34792672 10.1007/s10787-021-00890-z

[399] Huang J , Fu X , Chen X , Li Z , Huang Y , Liang C . Promising therapeutic targets for treatment of rheumatoid arthritis. Front Immunol. 2021;12:686155. 34305919 10.3389/fimmu.2021.686155 PMC8299711

[400] Matmati M , Jacques P , Maelfait J , et al. A20 (TNFAIP3) deficiency in myeloid cells triggers erosive polyarthritis resembling rheumatoid arthritis. Nat Genet. 2011;43(9):908‐912. 21841782 10.1038/ng.874

[401] Zhang LM , Zhou JJ , Luo CL . CYLD suppression enhances the pro‐inflammatory effects and hyperproliferation of rheumatoid arthritis fibroblast‐like synoviocytes by enhancing NF‐κB activation. Arthritis Res Ther. 2018;20(1):219. 30285829 10.1186/s13075-018-1722-9 PMC6169018

[402] Yang J , Xu P , Han L , et al. Cutting edge: ubiquitin‐specific protease 4 promotes Th17 cell function under inflammation by deubiquitinating and stabilizing RORγt. J Immunol. 2015;194(9):4094‐4097. 25821221 10.4049/jimmunol.1401451

[403] Kiriakidou M , Ching CL . Systemic lupus erythematosus. Ann Intern Med. 2020;172(11):Itc81‐itc96. 32479157 10.7326/AITC202006020

[404] Yu Y , Su Z , Wang Z , Xu H . USP7 is associated with greater disease activity in systemic lupus erythematosus via stabilization of the IFNα receptor. Mol Med Rep. 2017;16(2):2274‐2280. 28656291 10.3892/mmr.2017.6819

[405] Musone SL , Taylor KE , Lu TT , et al. Multiple polymorphisms in the TNFAIP3 region are independently associated with systemic lupus erythematosus. Nat Genet. 2008;40(9):1062‐1064. 19165919 10.1038/ng.202 PMC3897246

[406] De A , Dainichi T , Rathinam CV , Ghosh S . The deubiquitinase activity of A20 is dispensable for NF‐κB signaling. EMBO Rep. 2014;15(7):775‐783. 24878851 10.15252/embr.201338305 PMC4196981

[407] Odqvist L , Jevnikar Z , Riise R , et al. Genetic variations in A20 DUB domain provide a genetic link to citrullination and neutrophil extracellular traps in systemic lupus erythematosus. Ann Rheum Dis. 2019;78(10):1363‐1370. 31300459 10.1136/annrheumdis-2019-215434 PMC6788882

[408] Lee EG , Boone DL , Chai S , et al. Failure to regulate TNF‐induced NF‐kappaB and cell death responses in A20‐deficient mice. Science. 2000;289(5488):2350‐2354. 11009421 10.1126/science.289.5488.2350 PMC3582399

[409] Boehncke WH , Schön MP . Psoriasis. Lancet (London, England). 2015;386(9997):983‐994. 26025581 10.1016/S0140-6736(14)61909-7

[410] Fan YH , Yu Y , Mao RF , et al. USP4 targets TAK1 to downregulate TNFα‐induced NF‐κB activation. Cell Death Differ. 2011;18(10):1547‐1560. 21331078 10.1038/cdd.2011.11 PMC3136563

[411] Marcus R . What is multiple sclerosis?. JAMA. 2022;328(20):2078. 36413229 10.1001/jama.2022.14236

[412] Ten Bosch GJA , Bolk J , Laman JD . Multiple sclerosis is linked to MAPK(ERK) overactivity in microglia. J Mol Med. 2021;99(8):1033‐1042. 33948692 10.1007/s00109-021-02080-4 PMC8313465

[413] Hu H , Wang H , Xiao Y , et al. Otud7b facilitates T cell activation and inflammatory responses by regulating Zap70 ubiquitination. J Exp Med. 2016;213(3):399‐414. 26903241 10.1084/jem.20151426 PMC4813674

[414] Liu X , Li H , Zhong B , et al. USP18 inhibits NF‐κB and NFAT activation during Th17 differentiation by deubiquitinating the TAK1‐TAB1 complex. J Exp Med. 2013;210(8):1575‐1590. 23825189 10.1084/jem.20122327 PMC3727316

[415] Tacer KF , Potts PR . Cellular and disease functions of the Prader‐Willi syndrome gene MAGEL2. Biochem J. 2017;474(13):2177‐2190. 28626083 10.1042/BCJ20160616 PMC5594744

[416] Hao YH , Fountain MD , Fon Tacer K , et al. USP7 acts as a molecular rheostat to promote WASH‐dependent endosomal protein recycling and is mutated in a human neurodevelopmental disorder. Mol Cell. 2015;59(6):956‐969. 26365382 10.1016/j.molcel.2015.07.033 PMC4575888

[417] Homan CC , Kumar R , Nguyen LS , et al. Mutations in USP9X are associated with X‐linked intellectual disability and disrupt neuronal cell migration and growth. Am J Hum Genet. 2014;94(3):470‐478. 24607389 10.1016/j.ajhg.2014.02.004 PMC3951929

[418] Reijnders MR , Zachariadis V , Latour B , et al. De novo loss‐of‐function mutations in USP9X cause a female‐specific recognizable syndrome with developmental delay and congenital malformations. Am J Hum Genet. 2016;98(2):373‐381. 26833328 10.1016/j.ajhg.2015.12.015 PMC4746365

[419] Johnson BV , Kumar R , Oishi S , et al. Partial loss of USP9X function leads to a male neurodevelopmental and behavioral disorder converging on transforming growth factor β signaling. Biol Psychiatry. 2020;87(2):100‐112. 31443933 10.1016/j.biopsych.2019.05.028 PMC6925349

[420] Yoon S , Parnell E , Penzes P . TGF‐β‐induced phosphorylation of Usp9X stabilizes Ankyrin‐G and regulates dendritic spine development and maintenance. Cell Rep. 2020;31(8):107685. 32460012 10.1016/j.celrep.2020.107685 PMC7324065

[421] Lowther C , Costain G , Stavropoulos DJ , et al. Delineating the 15q13.3 microdeletion phenotype: a case series and comprehensive review of the literature. Genet Med. 2015;17(2):149‐157. 25077648 10.1038/gim.2014.83 PMC4464824

[422] Ben‐Shachar S , Lanpher B , German JR , et al. Microdeletion 15q13.3: a locus with incomplete penetrance for autism, mental retardation, and psychiatric disorders. J Med Genet. 2009;46(6):382‐388. 19289393 10.1136/jmg.2008.064378 PMC2776649

[423] Uddin M , Unda BK , Kwan V , et al. OTUD7A regulates neurodevelopmental phenotypes in the 15q13.3 microdeletion syndrome. Am J Hum Genet. 2018;102(2):278‐295. 29395074 10.1016/j.ajhg.2018.01.006 PMC5985537

[424] Yin J , Chen W , Chao ES , et al. Otud7a knockout mice recapitulate many neurological features of 15q13.3 microdeletion syndrome. Am J Hum Genet. 2018;102(2):296‐308. 29395075 10.1016/j.ajhg.2018.01.005 PMC5985472

[425] Garret P , Ebstein F , Delplancq G , et al. Report of the first patient with a homozygous OTUD7A variant responsible for epileptic encephalopathy and related proteasome dysfunction. Clin Genet. 2020;97(4):567‐575. 31997314 10.1111/cge.13709

[426] Santiago‐Sim T , Burrage LC , Ebstein F , et al. Biallelic variants in OTUD6B cause an intellectual disability syndrome associated with seizures and dysmorphic features. Am J Hum Genet. 2017;100(4):676‐688. 28343629 10.1016/j.ajhg.2017.03.001 PMC5384096

[427] Basar MA , Beck DB , Werner A . Deubiquitylases in developmental ubiquitin signaling and congenital diseases. Cell Death Differ. 2021;28(2):538‐556. 33335288 10.1038/s41418-020-00697-5 PMC7862630

[428] Beck DB , Basar MA , Asmar AJ , et al. Linkage‐specific deubiquitylation by OTUD5 defines an embryonic pathway intolerant to genomic variation. Sci Adv. 2021;7(4):eabe2116. 33523931 10.1126/sciadv.abe2116 PMC7817106

[429] Li H , Liu BJ , Xu J , et al. Design, synthesis, and biological evaluation of pyrido[2,3‐d]pyrimidin‐7(8H)‐one derivatives as potent USP1 inhibitors. Eur J Med Chem. 2024;275:116568. 38889606 10.1016/j.ejmech.2024.116568

[430] Sun Y , Sha B , Huang W , et al. ML323, a USP1 inhibitor triggers cell cycle arrest, apoptosis and autophagy in esophageal squamous cell carcinoma cells. Apoptosis. 2022;27(7‐8):545‐560. 35654870 10.1007/s10495-022-01736-x

[431] Chen J , Dexheimer TS , Ai Y , et al. Selective and cell‐active inhibitors of the USP1/UAF1 deubiquitinase complex reverse cisplatin resistance in non‐small cell lung cancer cells. Chem Biol. 2011;18(11):1390‐1400. 22118673 10.1016/j.chembiol.2011.08.014 PMC3344384

[432] Cheng YJ , Zhuang Z , Miao YL , et al. Identification of YCH2823 as a novel USP7 inhibitor for cancer therapy. Biochem Pharmacol. 2024;222:116071. 38387527 10.1016/j.bcp.2024.116071

[433] Zhuang Z , Miao YL , Song SS , et al. Discovery of pyrrolo[2,3‐d]pyrimidin‐4‐one derivative YCH3124 as a potent USP7 inhibitor for cancer therapy. Eur J Med Chem. 2024;277:116752. 39133975 10.1016/j.ejmech.2024.116752

[434] Xu X , Wang M , Xu H , et al. Design, synthesis and biological evaluation of 2‐aminopyridine derivatives as USP7 inhibitors. Bioorg Chem. 2022;129:106128. 36113266 10.1016/j.bioorg.2022.106128

[435] Murgai A , Sosič I , Gobec M , et al. Targeting the deubiquitinase USP7 for degradation with PROTACs. Chem Commun (Camb). 2022;58(63):8858‐8861. 35852517 10.1039/d2cc02094g PMC9710854

[436] Lu Y , Gao J , Wang P , et al. Discovery of potent small molecule ubiquitin‐specific protease 10 inhibitors with anti‐hepatocellular carcinoma activity through regulating YAP expression. Eur J Med Chem. 2024;272:116468. 38718626 10.1016/j.ejmech.2024.116468

[437] Burkhart RA , Peng Y , Norris ZA , et al. Mitoxantrone targets human ubiquitin‐specific peptidase 11 (USP11) and is a potent inhibitor of pancreatic cancer cell survival. Mol Cancer Res. 2013;11(8):901‐911. 23696131 10.1158/1541-7786.MCR-12-0699

[438] Kona SV , Kalivendi SV . The USP10/13 inhibitor, spautin‐1, attenuates the progression of glioblastoma by independently regulating RAF‐ERK mediated glycolysis and SKP2. Biochim Biophys Acta Mol Basis Dis. 2024;1870(7):167291. 38857836 10.1016/j.bbadis.2024.167291

[439] Göricke F , Vu V , Smith L , et al. Discovery and characterization of BAY‐805, a potent and selective inhibitor of ubiquitin‐specific protease USP21. J Med Chem. 2023;66(5):3431‐3447. 36802665 10.1021/acs.jmedchem.2c01933 PMC10009755

[440] Tak J , Nguyen TK , Lee K , Kim SG , Ahn HC . Utilizing machine learning to identify nifuroxazide as an inhibitor of ubiquitin‐specific protease 21 in a drug repositioning strategy. Biomed Pharmacother. 2024;174:116459. 38518599 10.1016/j.biopha.2024.116459

[441] Roy A , Sharma S , Paul I , Ray S . Molecular hybridization assisted multi‐technique approach for designing USP21 inhibitors to halt catalytic triad‐mediated nucleophilic attack and suppress pancreatic ductal adenocarcinoma progression: a molecular dynamics study. Comput Biol Med. 2024;182:109096. 39270458 10.1016/j.compbiomed.2024.109096

[442] Lu W , Chu P , Tang A , Si L , Fang D . The secoiridoid glycoside Gentiopicroside is a USP22 inhibitor with potent antitumor immunotherapeutic activity. Biomed Pharmacother. 2024;177:116974. 38968798 10.1016/j.biopha.2024.116974 PMC12772646

[443] Peng J , Jiang K , Sun X , et al. Identification of a class of potent USP25/28 inhibitors with broad‐spectrum anti‐cancer activity. Signal Transduct Target Ther. 2022;7(1):393. 36481755 10.1038/s41392-022-01209-2 PMC9731946

[444] Zhou D , Xu Z , Huang Y , et al. Structure‐based discovery of potent USP28 inhibitors derived from Vismodegib. Eur J Med Chem. 2023;254:115369. 37075624 10.1016/j.ejmech.2023.115369

[445] Shin SC , Park J , Kim KH , et al. Structural and functional characterization of USP47 reveals a hot spot for inhibitor design. Commun Biol. 2023;6(1):970. 37740002 10.1038/s42003-023-05345-5 PMC10516900

[446] Böhm K , Schulze‐Niemand E , Kähne T , et al. Synthesis and structure‐activity relationships of USP48 deubiquitinylase inhibitors. Arch Pharm. 2023;356(7):e2200661. 10.1002/ardp.202200661 37196427

[447] Tan L , Shan H , Han C , et al. Discovery of potent OTUB1/USP8 dual inhibitors targeting proteostasis in non‐small‐cell lung cancer. J Med Chem. 2022;65(20):13645‐13659. 36221183 10.1021/acs.jmedchem.2c00408

[448] Ren W , Xu Z , Chang Y , et al. Pharmaceutical targeting of OTUB2 sensitizes tumors to cytotoxic T cells via degradation of PD‐L1. Nat Commun. 2024;15(1):9. 38167274 10.1038/s41467-023-44466-7 PMC10761827

[449] Zhang Y , Du T , Liu N , et al. Discovery of an OTUD3 inhibitor for the treatment of non‐small cell lung cancer. Cell Death Dis. 2023;14(6):378. 37369659 10.1038/s41419-023-05900-2 PMC10300026

[450] Du T , Gu Q , Zhang Y , et al. Rolapitant treats lung cancer by targeting deubiquitinase OTUD3. CCS. 2024;22(1):195. 38539203 10.1186/s12964-024-01519-8 PMC10967203

[451] Su S , Chen J , Jiang Y , et al. SPOP and OTUD7A control EWS‐FLI1 protein stability to govern ewing sarcoma growth. Adv Sci (Weinh). 2021;8(14):e2004846. 34060252 10.1002/advs.202004846 PMC8292909

[452] Chen J , Bolhuis DL , Laggner C , et al. AtomNet‐aided OTUD7B inhibitor discovery and validation. Cancers. 2023;15(2):517. 36672466 10.3390/cancers15020517 PMC9856706

[453] Shi T , Bao J , Wang NX , Zheng J , Wu D . Identification of small molecule TRABID deubiquitinase inhibitors by computation‐based virtual screen. BMC Chem Biol. 2012;12:4. 22584113 10.1186/1472-6769-12-4 PMC3475094

[454] Zhang P , Xiao Z , Wang S , et al. ZRANB1 is an EZH2 deubiquitinase and a potential therapeutic target in breast cancer. Cell Rep. 2018;23(3):823‐837. 29669287 10.1016/j.celrep.2018.03.078 PMC5933875

[455] Chan WC , Liu X , Magin RS , et al. Accelerating inhibitor discovery for deubiquitinating enzymes. Nat Commun. 2023;14(1):686. 36754960 10.1038/s41467-023-36246-0 PMC9908924

[456] Grethe C , Schmidt M , Kipka GM , et al. Structural basis for specific inhibition of the deubiquitinase UCHL1. Nat Commun. 2022;13(1):5950. 36216817 10.1038/s41467-022-33559-4 PMC9549030

[457] Wang X , D'Arcy P , Caulfield TR , et al. Synthesis and evaluation of derivatives of the proteasome deubiquitinase inhibitor b‐AP15. Chem Biol Drug Des. 2015;86(5):1036‐1048. 25854145 10.1111/cbdd.12571 PMC4846425

[458] Wang X , Mazurkiewicz M , Hillert EK , et al. The proteasome deubiquitinase inhibitor VLX1570 shows selectivity for ubiquitin‐specific protease‐14 and induces apoptosis of multiple myeloma cells. Sci Rep. 2016;6:26979. 27264969 10.1038/srep26979 PMC4893612

[459] Paulus A , Akhtar S , Caulfield TR , et al. Coinhibition of the deubiquitinating enzymes, USP14 and UCHL5, with VLX1570 is lethal to ibrutinib‐ or bortezomib‐resistant Waldenstrom macroglobulinemia tumor cells. Blood Cancer J. 2016;6(11):e492. 27813535 10.1038/bcj.2016.93 PMC5148058

[460] Rowinsky EK , Paner A , Berdeja JG , et al. Phase 1 study of the protein deubiquitinase inhibitor VLX1570 in patients with relapsed and/or refractory multiple myeloma. Invest New Drugs. 2020;38(5):1448‐1453. 32125598 10.1007/s10637-020-00915-4 PMC7497669

[461] Wang L , Birch NW , Zhao Z , et al. Epigenetic targeted therapy of stabilized BAP1 in ASXL1 gain‐of‐function mutated leukemia. Nat Cancer. 2021;2(5):515‐526. 35122023 10.1038/s43018-021-00199-4

[462] Li J , Yakushi T , Parlati F , et al. Capzimin is a potent and specific inhibitor of proteasome isopeptidase Rpn11. Nat Chem Biol. 2017;13(5):486‐493. 28244987 10.1038/nchembio.2326 PMC5570473

[463] Schlierf A , Altmann E , Quancard J , et al. Targeted inhibition of the COP9 signalosome for treatment of cancer. Nat Commun. 2016;7:13166. 27774986 10.1038/ncomms13166 PMC5078989

[464] Chen S , Liu Y , Zhou H . Advances in the development ubiquitin‐specific peptidase (USP) inhibitors. Int J Mol Sci. 2021;22(9):4546. 33925279 10.3390/ijms22094546 PMC8123678

[465] Cruz L , Soares P , Correia M . Ubiquitin‐specific proteases: players in cancer cellular processes. Pharmaceuticals (Basel, Switzerland). 2021;14(9):848. 34577547 10.3390/ph14090848 PMC8469789

[466] Zhou L , Qin B , Yassine DM , et al. Structure and function of the highly homologous deubiquitinases ubiquitin specific peptidase 25 and 28: insights into their pathophysiological and therapeutic roles. Biochem Pharmacol. 2023;213:115624. 37245535 10.1016/j.bcp.2023.115624

[467] Patzke JV , Sauer F , Nair RK , et al. Structural basis for the bi‐specificity of USP25 and USP28 inhibitors. EMBO Rep. 2024;25(7):2950‐2973. 38816515 10.1038/s44319-024-00167-w PMC11239673

[468] Henning NJ , Boike L , Spradlin JN , et al. Deubiquitinase‐targeting chimeras for targeted protein stabilization. Nat Chem Biol. 2022;18(4):412‐421. 35210618 10.1038/s41589-022-00971-2 PMC10125259

[469] Advani AS , Cooper B , Visconte V , et al. A phase I/II trial of MEC (mitoxantrone, etoposide, cytarabine) in combination with ixazomib for relapsed refractory acute myeloid leukemia. Clin Cancer Res. 2019;25(14):4231‐4237. 30992301 10.1158/1078-0432.CCR-18-3886 PMC6635077

[470] Zhu Q , Fu Y , Li L , Liu CH , Zhang L . The functions and regulation of Otubains in protein homeostasis and diseases. Ageing Res Rev. 2021;67:101303. 33609777 10.1016/j.arr.2021.101303

[471] Fiil BK , Gyrd‐Hansen M . OTULIN deficiency causes auto‐inflammatory syndrome. Cell Res. 2016;26(11):1176‐1177. 27686184 10.1038/cr.2016.113 PMC5099863

[472] Shi Y , Wang X , Wang J , Wang X , Zhou H , Zhang L . The dual roles of A20 in cancer. Cancer Lett. 2021;511:26‐35. 33933552 10.1016/j.canlet.2021.04.017

[473] Li Y , Yang JY , Xie X , et al. Preventing abnormal NF‐κB activation and autoimmunity by Otub1‐mediated p100 stabilization. Cell Res. 2019;29(6):474‐485. 31086255 10.1038/s41422-019-0174-3 PMC6796864

[474] Li M , Li L , Asemota S , et al. Reciprocal interplay between OTULIN‐LUBAC determines genotoxic and inflammatory NF‐κB signal responses. Proc Nat Acad Sci USA. 2022;119(33):e2123097119. 35939695 10.1073/pnas.2123097119 PMC9388121

[475] Liu X , Zhang X , Peng Z , et al. Deubiquitylase OTUD6B governs pVHL stability in an enzyme‐independent manner and suppresses hepatocellular carcinoma metastasis. Adv Sci (Weinh). 2020;7(8):1902040. 32328410 10.1002/advs.201902040 PMC7175249

